# Discovery of New Quinolone-Based Diarylamides as Potent B-RAF^V600E^/C-RAF Kinase Inhibitors Endowed with Promising In Vitro Anticancer Activity

**DOI:** 10.3390/ijms24043216

**Published:** 2023-02-06

**Authors:** Hyun Ji Kim, Jung Woo Park, Sangjae Seo, Kwang-Hwi Cho, Mohammed M. Alanazi, Eun-Kyoung Bang, Gyochang Keum, Ashraf K. El-Damasy

**Affiliations:** 1 Center for Brain Technology, Brain Science Institute, Korea Institute of Science and Technology (KIST), Seoul 02792, Republic of Korea; 2 Supercomputing Application Center, Division of National Supercomputing, Korea Institute of Science and Technology Information, Daejeon 34141, Republic of Korea; 3 School of Systems Biomedical Science, Soongsil University, Seoul 06978, Republic of Korea; 4 Department of Pharmaceutical Chemistry, College of Pharmacy, King Saud University, Riyadh 11421, Saudi Arabia; 5 Division of Bio-Medical Science & Technology, KIST School, Korea University of Science and Technology (UST), Seoul 02792, Republic of Korea

**Keywords:** quinolines, diarylamides, B-RAF^V600E^, B-RAF^V600K^, C-RAF kinase, anticancer activity

## Abstract

The emergence of cancer resistance to targeted therapy represents a significant challenge in cancer treatment. Therefore, identifying new anticancer candidates, particularly those addressing oncogenic mutants, is an urgent medical demand. A campaign of structural modifications has been conducted to further optimize our previously reported 2-anilinoquinoline-diarylamides conjugate VII as a B-RAFV600E/C-RAF inhibitor. Considering the incorporation of a methylene bridge between the terminal phenyl and cyclic diamine, focused quinoline-based arylamides have been tailored, synthesized, and biologically evaluated. Among them, the 5/6-hydroxyquinolines **17b** and **18a** stood out as the most potent members, with IC_50_ values of 0.128 µM, 0.114 µM against B-RAF^V600E^, and 0.0653 µM, 0.0676 µM against C-RAF. Most importantly, **17b** elicited remarkable inhibitory potency against the clinically resistant B-RAF^V600K^ mutant with an IC_50_ value of 0.0616 µM. The putative binding mode of **17b** and **18a** were studied by molecular docking and molecular dynamics (MD). Moreover, the antiproliferative activity of all target compounds has been examined over a panel of NCI-60 human cancer cell lines. In agreement with cell-free assays, the designed compounds exerted superior anticancer impact over the lead quinoline **VII** against all cell lines at a 10 µM dose. Notably, both **17b** and **18b** showed highly potent antiproliferative activity against melanoma cell lines with growth percent under −90% (SK-MEL-29, SK-MEL-5, and UACC-62) at a single dose, while **17b** maintained potency with GI_50_ values of 1.60–1.89 µM against melanoma cell lines. Taken together, **17b**, a promising B-RAF^V600E/V600K^ and C-RAF kinase inhibitor, may serve as a valuable candidate in the arsenal of anticancer chemotherapeutics.

## 1. Introduction

Cancer is a primary life-threatening disease, coming to the fore as the second leading cause of global mortality with more than 600,000 estimated deaths in the United States according to the Centers for Disease Control and Prevention (CDC) in 2021 [1], and ranked sixth worldwide according to the World Health Organization (WHO) in 2019 [2]. Protein kinases and their relevant signaling pathways are essential in regulating cellular proliferation, differentiation, and apoptosis. However, the dysregulation of protein kinase activity due to genetic changes, such as translocations and mutations, along with overexpression, is implicated in the pathogenesis of various types of neoplasms. In this line, identifying new chemical entities targeting protein kinases constitutes a promising therapeutic approach for cancer treatment [3,4].

The RAF family kinases are the key components of the mitogen-activated protein kinase (MAPK) pathway, which encompasses the RAS-RAF-MEK-ERK cascade [5]. RAF has three isomers: A-, B-, and C-RAF. Among them, B-RAF is known as the primary contributor to phosphorylating the downstream MEK kinase. It has been reported that mutations of B-RAF were observed in about 30% of all cancer types. It accounts for ~70% of malignant melanomas, 36–53% of thyroid carcinomas, 30% of ovarian tumors, ~3% of lung cancers, 5–22% of colon cancers, and 1–4% of various other cancer types [6,7,8,9]. B-RAF^V600E^ is the most clinically observed mutant in melanoma, followed by the B-RAF^V600K^ mutant form, which exists in 10–30% of melanoma cases [10]. Dysregulation of either B-RAF^V600E^ or B-RAF^V600K^ promotes excessive cancer cell proliferation, and the role of B-RAF^V600K^ in inhibiting the apoptosis pathway has been reported [11]. C-RAF plays an important role in the phosphorylation of its *N*-terminal, providing RAF dimerization necessary for pathway progression [12]. However, A-RAF exhibits the weakest influence among the other RAF isoforms toward MEK activation [13,14]. Therefore, medicinal chemistry research on RAF kinase inhibitors is focused on designing new molecules that can inhibit such oncogenic proteins, particularly the mutant forms [15,16,17].

**Figure 1:**
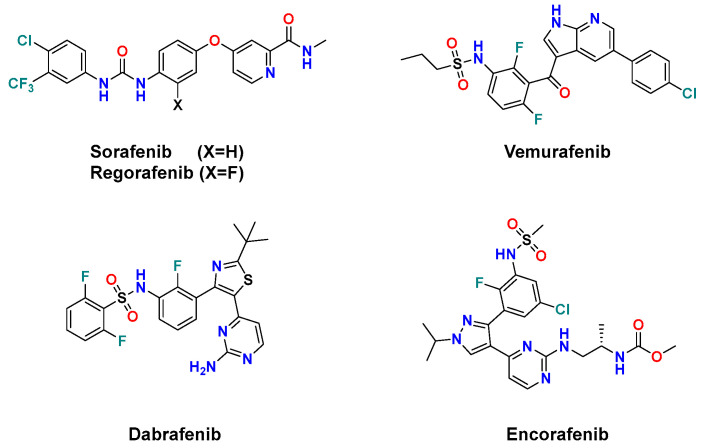
FDA-approved anticancer B-RAF/C-RAF inhibitors.

To date, five drugs have been approved by FDA as RAF inhibitors for cancer treatment. (Figure 1) Sorafenib, a diarylurea containing picolinamide [18], and its fluoro congener regorafenib [19] were approved in 2005 and 2013, respectively, as multi-kinase inhibitors targeting VEGFR, PDGFR, B-RAF, RET, and c-KIT [20]. However, they exhibited weak antiproliferative activity in melanoma cells harboring B-RAF^V600E^ mutation [21]. Such a drawback was overcome by the development of the 7-azaindole derivative vemurafenib [22] and the thiazole derivative dabrafenib [23]. These difluorophenyl sulfonamide drugs showed remarkably improved selectivity against the oncogenic B-RAF^V600E^ than its wild type by 100- and 500-fold, respectively [23,24]. However, vemurafenib and dabrafenib trigger a paradoxical activation of the MAPK pathway upon B-RAF inhibition, which results in specific adverse side effects and the emergence of resistance in many patients [14,23]. Therefore, they are clinically used with MEK inhibitors to achieve a more sustained inhibition of the MAPK pathway [24]. Most recently, the diarylpyrazole sulfonamide encorafenib was approved by the FDA in 2018 in combination with the MEK inhibitor binimetinib for the treatment of B-RAF^V600E/K^-mutant melanoma [25,26]. Encorafenib elicited better potency and had a longer residence time than vemurafenib and dabrafenib against B-RAF^V600E^ cells [21,27].

**Figure 2:**
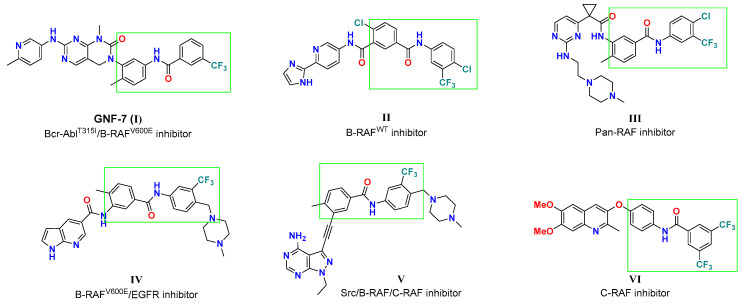
Representative examples of various diarylamides as RAF kinase inhibitors.

Among the plethora of small molecules targeting RAF kinase, diarylamides represent one of the substantial structural features (Figure 2) because of their favorable impact on kinase binding affinity and cellular potency. GNF-7 was reported as a potent Abl^T315I^/B-RAF^V600E^ inhibitor, with IC_50_ values of 61 and 6.1 nM, respectively [28,29]. The diarylamide tethered pyridine **II** was designed as a sorafenib analog, and moderately suppressed B-RAF activity (IC_50_ = 0.531 µM) [30]. The pyrimidine aminoethylpiperazine III was discovered as a pan-RAF inhibitor with two digits nanomolar IC_50_ values [31]. On the other hand, compounds **IV** and **V**, with 4-((4-methylpiperazin-1-yl)methyl)-3-(trifluoromethyl)pheny moiety, were identified as multiple kinase targeting agents. Compound IV was reported by Cheng et al. [32] as a dual B-RAF^V600E^/EGFR inhibitor with IC_50_ values of 51 and 8 nM, respectively. At the same time, the pyrazolopyrimidine **V** elicited significant inhibitory potency against Src, B-RAF^WT/V600E^ and C-RAF with IC_50_ values of 0.9, 15, 92, and 27 nM, respectively [33]. The diarylamides quinoline ether **VI** was reported to possess moderate selective potency toward C-RAF (77% inhibition at 10 µM) [34]. Structural analysis of those diarylamides discloses their similarity, as all feature *meta*-CF_3_ group at the terminal phenyl group which contribute to the inhibition activity of cancer-relevant RAF kinases.

**Figure 3:**
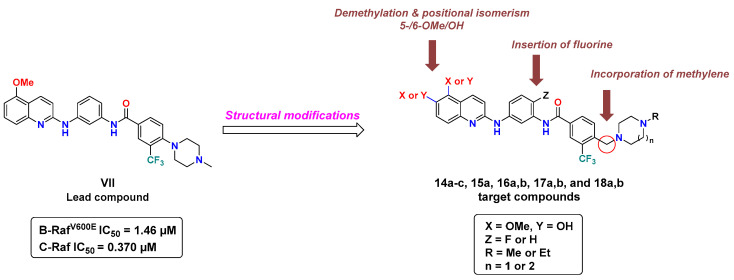
Structural modifications of lead compound **VII** for the discovery of novel RAF kinase inhibitors.

In a previous report, our group identified compound **VII** (Figure 3), diarylamides aminoquinoline, as a B-RAF^V600E^/C-RAF inhibitor with promising anti-proliferative activity against NCI-60 cancer cell lines, including melanoma [35]. However, **VII** exerted moderate enzymatic potency against B-RAF^V600E^ and C-RAF, with IC_50_ values of 1.46 and 0.370 µM, respectively. In the course of our ongoing endeavors to discover novel potent oncogenic kinase inhibitors [35,36,37,38], in the current investigation we planned to pursue a concise structure–activity relationship (SAR) analysis based on **VII** as a lead compound to improve its inhibitory potency against B-RAF^V600E^/C-RAF.

As revealed from the docking study of compound **VII** with B-RAF^V600E^, the 5-OMe group of quinoline engages with the kinase hinge region by hydrogen bonding, the terminal phenyl substituted with *N*-methyl piperazine and trifluoromethyl moieties share multiple hydrophobic interactions with DFG motif. In addition, the amide linker participates in a hydrogen bonding interaction with catalytic Glu501 [35]. Considering the role of these structural features in binding interactions, in this study we conducted a campaign of structural modifications to scout their impact on the activity. These molecular alterations of **VII** included three main aspects addressing the three essential binding regions in the catalytic kinase domain of RAF (Figure 3): (1) the introduction of a methylene bridge between the terminal phenyl ring and the cyclic diamine (*N*-methyl/*N*-ethylpiperazine, or *N*-methyl homopiperazine) that extends to occupy the allosteric pocket, encouraged by the potency of the abovementioned compounds (**IV** and **V**), (2) the introduction of fluorine in the central phenyl ring at the *ortho*-position of amide and the *para*-position of amine (similar to regorafenib), (3) exploring the role of methoxy and its corresponding hydroxyl group (hinge binding motifs) at both the 5- and 6-position of quinoline. We hypothesized that such structural changes would influence the hydrophobic-, halogen-, and hydrogen-bonding interactions and therefore make a difference in kinase inhibitory potency and cellular activity.

## 2. Results

### 2.1. Chemistry

**Scheme 1:**
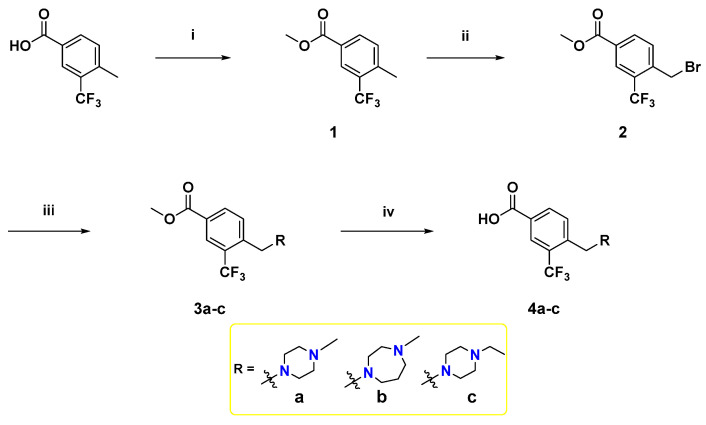
Reagents and reaction conditions: (**i**) methanol, H_2_SO_4_ (*cat.*), reflux, 3 h, 90%; (**ii**) NBS, AIBN, acetonitrile, reflux, overnight, 70%; (**iii**) appropriate *N*-alkyl cyclic diamine, K_2_CO_3_, DCM, rt, 2 h, 64%–89%; (**iv**) NaOH, methanol, 60 °C, 2 h, acid work-up, 76–100%.

To synthesize the target compounds, a set of three *meta*-trifluoromethylbenzoic acid derivatives have been synthesized as illustrated in Scheme 1. Fischer esterification of the commercially available 4-methyl-3-(trifluoromethyl)benzoic acid with methanol using a catalytic amount of H_2_SO_4_ afforded the benzoate ester **1**. Next, the treatment of **1** with *N*-bromosuccinimide (NBS) and azobisisobutyronitrile (AIBN) in acetonitrile at reflux temperature afforded the corresponding bromobenzyl derivative **2**, which then underwent S_N_2 reaction with the appropriate cyclic diamines in dichloromethane (DCM) using K_2_CO_3_ as a base [39] to yield **3a**–**c**. Lastly, base-catalyzed ester hydrolysis of **3a**–**c** was performed to furnish the desired 3,4-disubstitutedbenzoic acid derivatives **4a**–**c** [40].

**Scheme 2:**
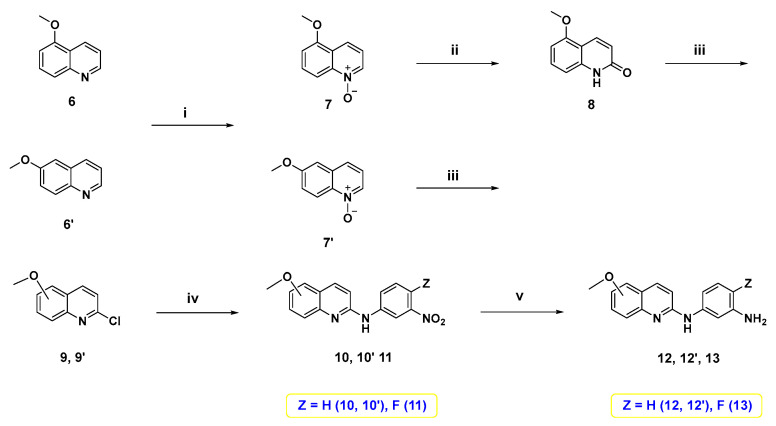
Reagents and reaction conditions: (**i**) m-CPBA, DCM, 0 °C to rt, 3–6 h; (**ii**) *p*-TsCl, 10% aq. K_2_CO_3_, DCM, rt, 18 h, 65%; (**iii**) POCl_3_, reflux, 1 h, 41–86%; (**iv**) 3-nitroaniline or 4-fluoro-3-nitroaniline, neat, 160 °C, 3 h; (**v**) Pd/C, H_2_, methanol, rt, 12 h, 69–81%.

On the other hand, 5- or 6-methoxy quinoline-based diamine intermediates **12**, **12′**, and **13** were synthesized as shown in Scheme 2. Starting from 5-methoxyquinoline (**6**), which was synthesized following our previous study [41], or its corresponding 6-methoxy isomer **6′**, *N*-oxide intermediates **7** and **7′** were synthesized through oxidation with *meta*-chloroperoxybenzoic acid (*m*-CPBA). Treatment of *N*-oxide **7** with *p*-tosyl chloride and K_2_CO_3_ in DCM afforded the lactam form of quinoline **8**. Treatment of **7′** and **8** with POCl_3_ furnished the 2-chloroquinoline derivatives **9** and **9′**. Nucleophilic aromatic substitution of **9** and **9′** with *meta*-nitroaniline derivative produced the corresponding nitroanilines **10**, **10′**, and **11**. Reduction of these nitro derivatives using palladium on carbon catalyst under hydrogen atmosphere [42] yielded 5- or 6-methoxy quinoline-based diamine intermediates **12**, **12′**, and **13**.

**Scheme 3:**
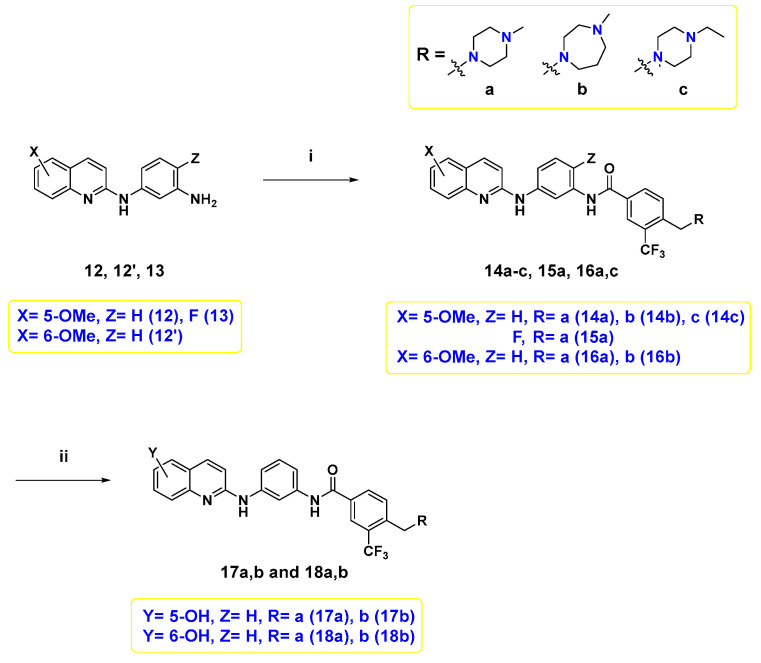
Reagents and reaction conditions: (**i**) **4a**–**c**, HATU, DIPEA, DMF, rt, overnight, 9.4–44%; (**ii**) 1.0 M BBr_3_ in DCM, 0 °C to rt, overnight, 4.5–46%.

Finally, the target compounds were prepared following Scheme 3. The first six target compounds **14a**–**c**, **15a**, **16a,** and **16b** were obtained by an amide coupling reaction between diamine **12**, **12′**, and **13**, and the proper benzoic acid derivative **4a**–**c** with HATU and *N*,*N*-diisopropylethylamine (DIPEA) in *N*,*N*-dimethylformamide (DMF) under inert atmosphere. Demethylation of **14a**,**b**, and **16a**,**b** was conducted using BBr_3_ to afford their corresponding hydroxyl counterparts, **17a**,**b**, and **18a**,**b**.

### 2.2. In Vitro Kinase Inhibition Assay

The inhibitory effects of all synthesized compounds against B-RAF^V600E^ and C-RAF kinases were investigated using the HotSpot^TM^ kinase assay, at Reaction Biology Corporation (RBC), with GW5074 (structure is shown in Supplementary Materials) as a positive control, and the results are listed in Table 1.

As disclosed from the biochemical assay results, all tested compounds showed promising activities with sub-micromolar IC_50_ values, which is about 2–10-fold superior to the lead compound **VII** (Table 1). Comparing the IC_50_ of **VII** (B-RAF^V600E^; 1.46 µM, C-RAF; IC_50_ = 0.370 µM) and **14a** (B-RAF^V600E^; IC_50_ = 0.498 µM, C-RAF; IC_50_ = 0.151 µM), it was found that the incorporation of a methylene bridge between the terminal phenyl and piperazine resulted in improved inhibitory potency against both kinases. The same conclusion applies upon inspecting the activity of **14c** and **14b** (**14b**, B-RAF^V600E^; IC_50_ = 0.355 µM, C-RAF; IC_50_ = 0.192 µM; **14c**, B-RAF^V600E^; IC_50_ = 0.619 µM, C-RAF; IC_50_ = 0.182 µM), which further emphasized the favorable contribution of the methylene moiety in strengthening the binding interactions with DFG motif of B-RAF^V600E^ and C-RAF kinases. Regarding **15a**, in which the hydrogen at the *ortho*-position to the amide of the central phenyl ring was substituted with fluorine from **14a**, inhibition was slightly increased against B-RAF^V600E^ (**14a, 15a**: B-RAF^V600E^; IC_50_ = 0.498, 0.314 µM, respectively), which may stem from the enhanced halogen interaction of fluorine with B-RAF^V600E^.

**Figure 4:**
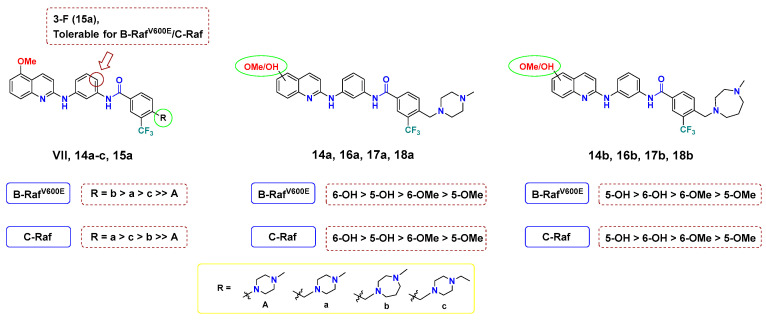
SAR analysis of final compounds **14a**–**c**, **15a**, **16a**,**b**, **17a**,**b** and **18a**,**b** for B-RAF^V600E^/C-RAF.

Meanwhile, when the connection of the methoxy groups was shifted from 5- to the 6-position of quinoline, there was slight tendency to improve the activity. For example, the 6-hydoxyquinoline **16a** (B-RAF^V600E^; IC_50_ = 0.193 µM, C-RAF; IC_50_ = 0.103 µM) exerted better inhibitory potency than its corresponding 6-methyoxy congener **14a** (B-RAF^V600E^; IC_50_ = 0.498 µM, C-RAF; IC_50_ = 0.151 µM). This finding might be attributed to the ability of the -OH group to form a tight hydrogen bond interaction with the hinge region of the kinase backbone. Particularly, the substitution of a hydroxyl group at the 5-position of quinoline achieved considerably improved potency towards both B-RAF^V600E^ and C-RAF by 2.8–3.6- and 1.6–2.9-fold, respectively. (**14a**, **17a**: B-RAF^V600E^; IC_50_ = 0.498, 0.137 µM, C-RAF; IC_50_ = 0.151, 0.0950 µM.) Grafting the hydroxyl group of **17a** with *N*-methylpiperazine (B-RAF^V600E^; IC_50_ = 0.137 µM, C-RAF; IC_50_ = 0.0950 µM) to 6-position **18a** was favorable for activity (B-RAF^V600E^; IC_50_ = 0.114 µM, C-RAF; IC_50_ = 0.0653 µM); however, this observation was reversed in the case of *N*-methylhomopiperazine derivatives **17b** and **18b**. At last, for the change in the R moiety, it was more favorable for the activity to install *N*-methylhomopiperazine in the case of 5-OMe or 5-OH, and *N*-methylpiperazine in the case of 6-OMe or 6-OH, but the difference was marginal. A summary of SAR is illustrated in Figure 4.

Taken together, it is thought that a change in the interaction with B-RAF^V600E^ and C-RAF kinases, caused by additional methylene and a hydroxyl group, greatly influenced activity. Accordingly, two highly potent B-RAF^V600E^ and C-RAF inhibitors, **17b** and **18a,** have been discovered. To gain insight into the inhibitory mode of this new array of quinolines, **18a** was tested at 1 µM concentration of ATP to compare it with its IC_50_ at 10 µM (Table 2). As shown in Table 2, **18a** showed IC_50_ values of 0.0843 and 0.0293 µM against B-RAF^V600E^ and C-RAF, respectively, at 1 µM ATP. This biochemical outcome points out that the IC_50_ values vary depending on the ATP concentration, which indicates that **18a** acts as a competitive ATP kinase inhibitor. Moreover, the inhibitory activity of **17b** and **18a** was tested against the clinically resistant B-RAF^V600K^**,** for which many RAF inhibitors are inactive. Interestingly, both **17b** and **18a** elicited promising inhibitory potency against B-RAF^V600K^, with IC_50_ values of 61.6 and < 0.5 nM, respectively.

### 2.3. In Vitro Screening of the Anticancer Activities

#### 2.3.1. Single Dose Testing against NCI-60 Cell Line Panel

Encouraged by the promising results in the cell-free assay, the antiproliferative activity of all final compounds was evaluated by the National Cancer Institute (NCI) in a single dose of 10 µM over a library of 60 human cancer cell lines representing nine cancer types; leukemia, non-small cell lung (NSCL), colon, central nervous system (CNS), melanoma, ovarian, renal, prostate, and breast cancer.

**Figure 5:**
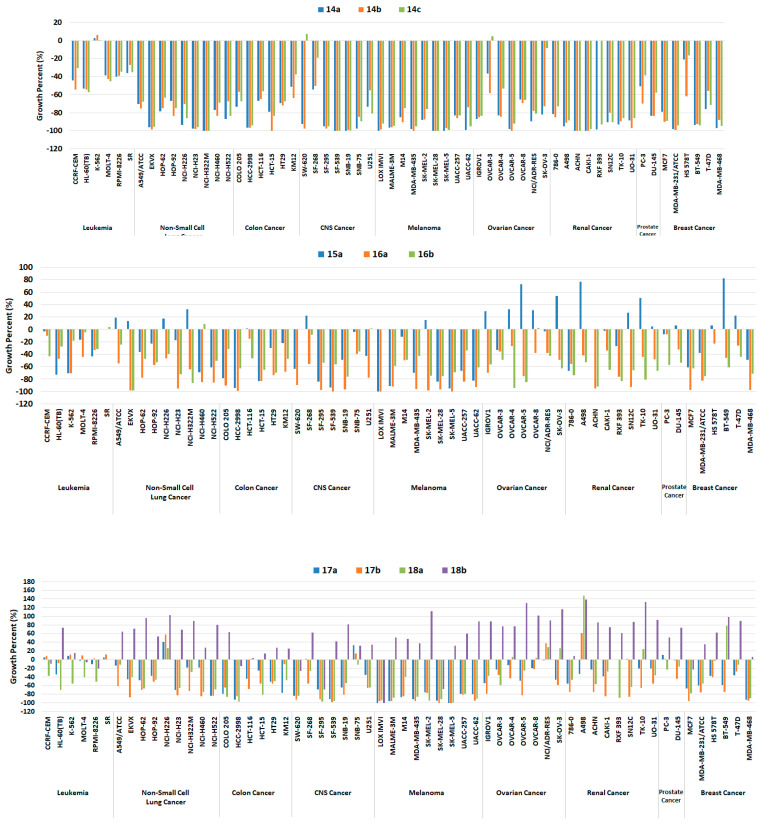
Growth percentage of the NCI-60 human tumor cell lines upon treatment with 10 μM of compounds **14a**–**c** (upper panel), **15a**, **16a**, and **16b** (middle panel), **17a**, **17b**, **18a**, and **18b** (lower panel).

Generally, all target compounds, except **18b**, showed significantly improved antiproliferative effects in almost all cancer cell lines (Figure 5). Compared with the lead compound **Vll**, **14a**–**c** with methylene between the bridge of the terminal phenyl ring and the R groups were endowed with broad growth inhibitory activity. In particular, the antiproliferative activity against melanoma, which is mostly driven by RAF mutations, was greatly improved as indicated by −100 up to −70 growth percent (minus growth percent indicates cell lethal effect). Such cellular outcomes were in agreement with the findings observed in the biochemical kinase assay (Table 1). Furthermore, **14a**–**c** exhibited cell growth percents below −90 in some cell lines belonging to NSCL, colon, CNS, ovarian, renal, and breast cancer, and even caused more sound lethal effects towards some cell lines with growth percent −100, including NCI-H322M (NSCL cancer), HCT-15 (colon cancer), SF-539, SNB-19 (CNS cancer), LOX IMVI, MDA-MB-435, SK-MEL-28, SK-MEL-5 (melanoma), OVCAR-5 (ovarian cancer), ACHN and CAKI-1 (renal cancer).

As for **15a**, overall antiproliferative activity was relatively decreased compared to **14a**–**c**; in particular, although the inhibition ability against B-RAF^V600E^ of **15a** was slightly greater, it did not inhibit the growth of melanoma cells, except LOX IMVI and UACC-62 in a better fashion. Additionally, both 6-methoxyquinoline-based diarylamides **16a** and **16b** showed considerable worthy growth inhibitory ability over all cell lines. In particular, **16a** was slightly better than **16b** against all melanoma cells, which is the same trend observed with the kinase assay findings.

Meanwhile, the growth percent of compounds **17a**, **17b**, and **18a**, in which the methoxy group was replaced with the hydroxyl group, was moderately decreased for most cells; however, it maintained at a high level for melanoma cell lines (Figure 5). They were lethal to some melanoma cells against LOX IMVI, SK-MEL-28, and SK-MEL-5 with growth percent −100, and against MALME-3M, MDA-MB-435, SK-MEL-2, and UACC-62 cell lines, the growth percent values were less than -90. On the other hand, unlike other related compounds, the growth percentage of **18b** was greatly reduced for all cells, and although it had a lethal effect against the LOX IMVI cell line, it lost the antiproliferative ability to other melanoma cell lines greatly. This finding may be attributed to the decreased RAF inhibitory potency of **18b**.

#### 2.3.2. Five-Dose Testing against NCI-60 Cell Line Panel

To deeply investigate the antiproliferative activity of final compounds, the further assay was processed in a five-dose testing mode, using sorafenib as a reference compound (Table 3 and Table 4). In a similar fashion to that observed in the single dose assay, compounds **14a**–**c** demonstrated distinct antiproliferative ability while showing excellent GI_50_ spanning the range of 1.20–11.8 µM for all cancer cell lines. Towards melanoma cell lines, except SK-MEL-2, GI_50_ values of **14a**–**c** were less than 2.0 µM concentration, while over the majority of the other cell lines, including leukemia, NSCL, colon, CNS, renal, and breast cancer, **14a**–**c** showed a similar level of GI_50_.

Contrary to the single-dose mode, the antiproliferative property of **15a** significantly reduced GI_50_ values of 3.61–18.8 µM for all cells and 11.7–16.3 µM for melanoma cells, respectively, despite showing slightly better inhibition of B-RAF^V600E^ when compared with **14a**. We speculate that **15a** may be promising at the molecular level, yet has a poor growth inhibitory potency at the cellular level, which may be due to low solubility/or permeability endowed by fluorine. This discrepancy seems to be observed in the case of **16a** as well. Comparing the results of **14a** and **16a**, the antiproliferative ability of **16a** decreased considerably against all cancer cell lines, except 12-cell lines (leukemia; K-562, SR, NSCL; HOP-92, NCI-H226, colon; HCT-15, HT29, CNS; SNB-75, melanoma; LOX IMVI, ovarian; SK-OV-3, breast; MCF7, BT-549, MDA-MB-468), only by the change of the methoxy group substitution position, from 5 to 6, of quinoline. Specifically, for melanoma cells, antiproliferative activity potency was maintained for only LOX IMVI cells (GI_50_ = 1.55 µM), but not for the other cells. Likewise, **16a** showed better kinase inhibition activity compared to **14a** for B-RAF^V600E^ and C-RAF (2.6-fold and 1.5-fold, respectively); we expected that the result of **16a** will also have a similar reason as **15a**.

Referring to the hydroxyquinolines, the antiproliferative potency of **17a**,**b** tended to exert lower potency as indicated by GI_50_ values over 2.0 µM against all leukemia cell lines, except MOLT-4 (GI_50_ = 1.86 µM). These observations were similar against NSCL cancer cell lines, and in particular, for NCI-H226, the cellular potency was decreased by about 3-fold at least (**14a**, **17a**; GI_50_ = 4.63, 19.2 µM, respectively). For colon and CNS cancer cells, the moderate GI_50_ values were maintained at a maximum of 3.81 µM (**17a**; SF-268 of CNS) or less. Fortunately, against melanoma cells, the potent antiproliferative activity was maintained. In particular, against SK-MEL-2, **17a** and **17b** elicited slightly better antiproliferative ability (**14a**, **17a**: GI_50_ = 3.52, 1.92 µM, respectively), although against the M14 cell line, the GI_50_ of **17a** was decreased slightly (**14a**, **17a**: GI_50_ = 1.76, 2.21 µM, respectively). For the rest of the cancer cell lines, a similar trend of GI_50_ values was noticed. However, the results of representative compounds **18a** and **18b** with the 6-OH group turned out to be disappointing. These compounds generally showed reduced antiproliferative activity compared to **17a** and **17b**, and a noticeably modest effect against NSCL and colon cell lines (GI_50_ against NSCL and colon: 3.39–17.8 µM and 2.09–18.5 µM, respectively.). Additionally, for CNS cancer cell lines, antiproliferative activity was decreased by at least 2.7-fold; however, **18a** showed significant potency with a submicromolar GI_50_ value of 0.656 µM against SNB-75. While the GI_50_ values of **18a** against the LOX IMVI, MALME-3M, and SK-MEL-28 cell lines remained at a similar level to **17a**, maintaining GI_50_ 1.59–1.79 µM, the GI_50_ values were increased by 1.37–9.11-fold against other melanoma cell lines. Strangely, the cellular potency of **18b** was somehow modest, with a GI_50_ range 10.4–17.2 µM, and both **18a** and **18b** also exhibited less efficacy in inhibiting cell growth against the rest of the cancer cell lines. Based on the findings of the kinase assay, **18b** could be expected to have a reduced antiproliferative ability against melanoma cells, but although **18a** was the most potent against B-RAF^V600E^, its antiproliferative ability against melanoma cells was shown to be insufficient. Therefore, the 6-OMe group of quinoline may be influential at kinase inhibition but is associated with a decrease in antiproliferative activity in the cells. Based on these findings, we identified **17b** as the most potent member showing excellent inhibitory ability even on cancer cells, and its broad antiproliferative spectrum offered the possibility that it can target other molecular targets in the cellular context.

While comparing with sorafenib, the first FDA-approved RAF inhibitor, **17b** was more potent than sorafenib in some cell lines. Against colon cancer, **17b** exerted slightly better potency than sorafenib, except KM12 cells, with GI_50_ values less than 2.0 μM. A similar finding was also noticed for melanoma cells. Except for SK-MEL-5 cells, **17b** had better GI_50_ values of less than 2.0 μM over all cell lines. Interestingly, **17b** elicited superior antiproliferative activity than sorafenib against all renal cancer cell lines, except A498 cells. Since sorafenib is clinically used for the treatment of renal cell carcinoma, these findings suggested hope that **17b** may be a promising candidate for renal cancer. On the other hand, the antiproliferative potency of **17b** was reduced against ovarian cancer cell lines, except IGROV1, OVCAR-3, and OVCAR-8. At the same time, its anticancer activity was comparable to sorafenib over the remaining tested cell lines.

### 2.4. Molecular Docking Study

**Figure 6:**
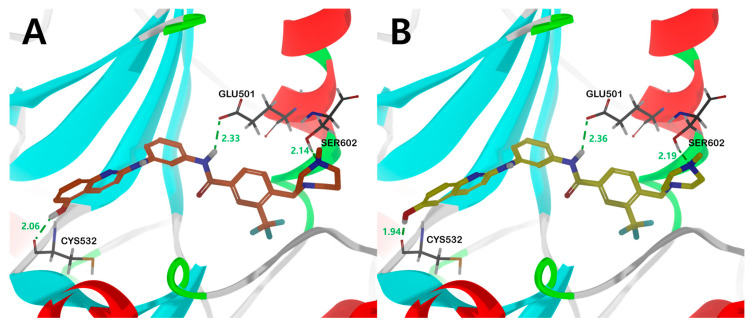
The binding mode of compound (**A**) **17b** and (**B**) **18a** in the catalytic kinase domain of B-RAF^V600E^ model. For clarity purposes, only key residues having interactions were shown. Compounds **17b** and **18a** are shown in orange and yellow colored stick models, respectively. Fundamental hydrogen bonding interactions are shown in green dashes. The number near the dashes indicates the bonding distance in units of Å.

**Figure 7:**
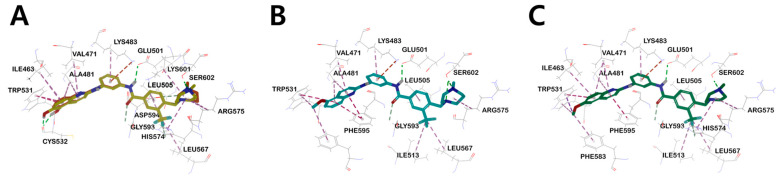
(**A**) Three-dimensional structural overlay of compounds **17b** (orange) and **18a** (yellow) inside B-RAF^V600E^ protein. The binding mode of compound (**B**) **14b** (sky blue), (**C**) **16a** (green) in the B-RAF^V600E^ protein. Inhibitors are shown in the stick model, and surrounding residues are shown in the line models. Various interactions are shown in dashes.

In order to rationalize the observed RAF kinase inhibitory results from a 3D structural perspective, **14b**, **16a**, **17b**, and **18a** were docked in the catalytic kinase domains of B-RAF^V600E^ (PDB code: 1UWJ) [44] and C-RAF homology models. Molecular docking studies were performed by inserting quinoline-based diarylamide compounds into the sorafenib binding sites of B-RAF^V600E^ and C-RAF homology models. Both B-RAF^V600E^ and C-RAF homologous proteins bound well and showed similar binding modes (Figure 6 and Figure 7, Figures S1–S5).

Compounds **17b** and **18a**, with sound kinase effects, were calculated to have a nearly similar binding mode to the B-RAF^V600E^ protein. The three significant hydrogen bonds are (1) between the hydrogens of the amine moiety in the central amide bond and the side-chain carboxylate of the catalyst Glu501, (2) between the hydroxyl hydrogens of the quinoline moiety and the amino carbonyl oxygens of Cys532 in the hinge region, and (3) between the hydroxyl group of Ser602 and the nitrogen of *N*-methylpiperazine or *N*-methyl-1,4-diazepane (Figure 6).

Interestingly, in the case of **14b** and **16a,** the methoxy derivatives of **17b** and **18a**, no major hydrogen bond with the Cys532 residue was found. Through this, it was suggested that the substituent providing hydrogen that can form a hydrogen bond with Cys532 plays an essential role in kinase efficacy (Figure 6).

Similar to the results of docking simulations in previous studies, these quinoline moieties consistently occupy the ATP adenine binding site of B-RAF^V600E^ through π–alkyl interactions with surrounding hydrophobic moieties in all compounds. The aromatic residue of quinoline π–π interacts with Trp531 of the hinge region and quinoline π–alkyl interacts with the side-chain alkyl group of Val471, Ile463, and Ala481 of the P-loop in B-RAF^V600E^. The central phenyl ring of the inhibitors interacts with the aliphatic side chain of Lys483. The carbon–hydrogen bonds were formed between the hydrogen of the carboxyl group in the central amide bond and the hydrogen of Cαin Gly593. The trifluoromethyl phenyl ring shared π–alkyl interactions with the side-chain alkyl group of Leu505. For heterocyclic ring sizes, such as *N*-methylpiperazine and *N*-methyl-1,4-diazepane, no major binding mode that could affect the efficacy with B-RAF was found, and the alkyl interaction between the heterocyclic ring and Arg575 was common. Therefore, through docking studies, it can be suggested that the hydroxyl group at the 5- and 6-positions of quinoline has a greater effect on RAF kinase efficacy than the effect of the bulky heterocyclic ring. The mode of binding between C-RAF kinase and inhibitors **17b** and **18a** is mainly similar to that of B-RAF^V600E^. However, among the three primary hydrogen bonds, only two hydrogen bonds between the hydrogen of the amine residue in the central amide bond and the side chain carboxylate of the catalyst Glu393, and between the hydroxyl hydrogen of the quinoline residue and the amino carbonyl oxygen of Cys424 were shown. Interestingly, the distances of major hydrogen bonds were generally shorter in C-RAF than in B-RAF^V600E^, suggesting a tighter bond between inhibitor and kinase in C-RAF than in B-RAF^V600E^ (Figure S3).

### 2.5. Molecular Dynamics

**Figure 8:**
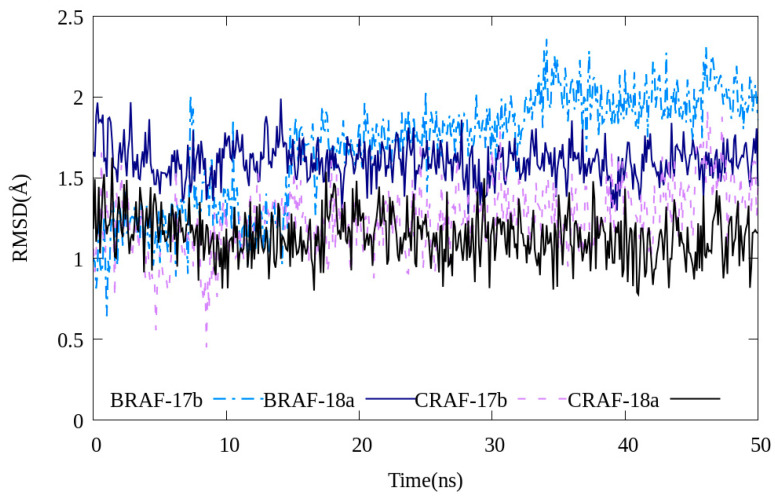
RMSD values of compounds **17b** and **18a** during 50 ns of molecular dynamics simulations.

Molecular simulations were performed to investigate the stability of **17b** and **18a** binding to C-RAF and B-RAF^V600E^, respectively. Both compounds interacted with each protein maintaining relative stability (RMSD value < 2.5 Å, Figure 8). Even when the MD simulation execution time was increased to 120 ns, the fluctuation values of the RMSD values of the two compounds **17b** and **18a** were not large (Figure S6). Through this, it can be seen that the two compounds and the B-RAF^V600E^ protein reached a relatively stable interaction state.

In addition, the bonding distances of the three major hydrogen-bonding interactions between ligands and proteins presented in molecular docking studies (Figure 6) were monitored by MD simulations (Figures S7 and S8). Among them, the Cys532 and Glu501 residues showed very small distance changes, confirming that these two hydrogen bonds are the main interaction sites of ligand–protein bonding. In the case of the Ser602 residue, the distance change was calculated to be large compared to the other two residues. This is interpreted as a less stable bond than the other two hydrogen bonds because the Ser602 residue was modeled as a missing residue using the GalaxyFill algorithm and used for calculation and is also located in a ring with relatively many tertiary structural changes.

Table 5 lists the calculated binding free energies of the most potent compounds **17b** and **18a**. Based on the RMSD plot, it was confirmed that the docking pose of each inhibitor did not deviate significantly from the initial state during the simulation process. In addition, the molecular dynamics simulation results show good agreement with the IC_50_ results. In both proteins, **18a** produces a smaller binding free energy, indicating a higher binding affinity. In addition, all of the selected compounds obtained a smaller binding free energy to C-RAF than to B-RAF^V600E^, indicating that the compounds showed stronger binding to CRAF.

## 3. Conclusions

In this report, a concise set of ten new quinoline-based diarylamides have been designed and synthesized to improve the RAF inhibitory potency of our previously reported lead **VII**. Herein, multidimensional structural modifications of lead compound **Vll** have been performed, including the introduction of methylene between the terminal phenyl ring and cyclic diamine, the substitution of fluorine and installing hydroxyl groups at the central phenyl ring, and the 5-/6-position of quinoline, respectively. All the target compounds have been tested against B-RAF^V600E^ and C-RAF at a 10 µM concentration of ATP. The SAR study pointed out that the additional methylene and hydroxyl group resulted in notable increased inhibitory activity. Among the newly designed members, **17b** and **18a** emerged as the most potent compounds against B-RAF^V600E^ and C-RAF. Moreover, **17b** elicited nanomolar potency against the clinically resistant mutant B-RAF^V600K^. Compound **18a** showed a better IC_50_ value at 1 µM concentration of ATP, indicating that these compounds are ATP-competitive kinase inhibitors. Single-dose assay of all compounds over a panel of NCI-60 human cancer cell lines indicated that, consistent with the kinase assay, additional methylene led to a broad antiproliferative advantage. In the five-dose testing mode, **17b** maintained highly potent antiproliferative activity against melanoma cell lines. Given both cell-free and cell-based assay findings, **17b** was discovered as a promising RAF-inhibitor candidate with the potential for cancer treatment, especially oncogenic RAF-derived melanoma.

## 4. Materials and Methods

### 4.1. Chemistry

General: All reagents and anhydrous solvents were purchased from Sigma-Aldrich Korea, TCI, and Combi-blocks and used without further purification. Unless otherwise noted, the reactions were carried out under an argon atmosphere and monitored on thin-layer chromatography (TLC) plates (Merck, silica gel 60 F_254_). TLC plates were observed using UV light (254 nm and 365 nm) and ninhydrin or phosphomolybdic acid (PMA) staining solution. Silica gel (Merck, 230–400 mesh) was used for flash column chromatography. ^1^H and ^13^C nuclear magnetic resonance (NMR) spectra were obtained by measuring with Bruker Advance III 400 MHz spectrometers, and CDCl_3_-*d*, methanol-*d*_4_, DMSO-*d*_6_ and acetone-*d*_6_ from Cambridge Isotope laboratories were used as NMR solvents. Chemical shift (δ) was recorded in ppm [45] using tetramethylsilane (TMS) as an internal standard, and the abbreviations s (single), d (doublet), t (triplet), m (multiplet), br. s (broad singlet), dd (doublet of doublet), or dt (doublet of triplet). The coupling constant (*J*) was recorded in hertz (Hz). High-resolution mass spectra (HRMS) were obtained by measuring with Waters SYNAPT G2 mass spectrometer ESI-TOF mode. Compounds **6**, **9**, and **12** were prepared following the reported procedures references [35,37].

### 4.2. Synthesis

#### 4.2.1. Methyl 4-methyl-3-(trifluoromethyl)benzoate (**1**)

To a solution of 4-methyl-3-(trifluoromethyl)benzoic acid (5.00 g, 24.5 mmol) in anhydrous methanol (80.0 mL), H_2_SO_4_ (8.50 mL) was added dropwise. The reaction mixture was stirred under reflux overnight, cooled down, and the solvent was evaporated under reduced pressure. NaHCO_3_ (aq.) was added dropwise to the residue in an ice bath until slightly basic (pH = 8), then extracted with ethyl acetate (20 × 3 mL). The combined organic layer was washed with brine, and dried over anhydrous Na_2_SO_4,_ and the solvent was evaporated under reduced pressure to afford the titled product **1** (4.83 g, 90% yield) as a yellow oil. ^1^H NMR (400 MHz, CDCl_3_) δ 8.27 (s, 1H), 8.07 (d, *J* = 8.0 Hz, 1H), 7.35 (d, *J* = 8.0 Hz, 1H), 3.92 (s, 3H), 2.53 (s, 3H); ^13^C NMR (100 MHz, CDCl_3_) δ 165.89, 142.06 (d, *J* = 1.2 Hz), 132.64, 132.20, 129.24 (q, *J* = 30 Hz), 128.26, 127.10 (q, *J* = 5.6 Hz), 124.18 (q, *J* = 272 Hz), 52.22, 19.39 (d, *J* = 1.9 Hz).

#### 4.2.2. Methyl 4-(bromomethyl)-3-(trifluoromethyl)benzoate (**2**)

To a solution of **1** (4.39 g, 20.1 mmol) in anhydrous acetonitrile (15.0 mL), NBS (4.30 g, 24.1 mmol) and AIBN (1.30 g, 7.92 mmol) were added and stirred under reflux overnight. The reaction mixture was cooled, and distilled H_2_O was added to quench the reaction, then extracted with ethyl acetate (20 × 3 mL). The combined organic layer was washed with brine solution, and dried over anhydrous Na_2_SO_4_, and the solvent was evaporated under reduced pressure to afford residue **2** (4.18 g, 70% yield) as a yellow oil. The residue was used in the next step without further purification. ^1^H NMR (400 MHz, CDCl_3_) δ 8.31 (s, 1H), 8.20 (dd, *J* = 8.1, 1.4 Hz, 1H), 7.69 (d, *J* = 8.1 Hz, 1H), 4.64 (s, 2H), 3.96 (s, 3H); ^13^C NMR (100 MHz, CDCl_3_) δ 165.35, 140.91, 133.34, 133.11, 130.57, 128.64 (q, *J* = 31 Hz), 127.63 (q, *J* = 5.7 Hz), 123.71 (q, *J* = 273 Hz), 52.68, 27.48 (d, *J* = 2.6 Hz).

#### 4.2.3. General Procedure for the Synthesis of Benzoate **3a**–**c**

To a solution of **2** (1.0 eq.) and appropriate cyclic amine (3.0 eq.) in anhydrous DCM (20 mL), K_2_CO_3_ (3.0 eq) was added. The reaction mixture was stirred at room temperature for 2 h, then extracted with distilled water (20 × 3 mL). The combined organic layers were washed with brine solution, dried over anhydrous Na_2_SO_4_, and filtered. The solvent was evaporated under reduced pressure, and the residue was purified by silica gel flash column chromatography using proper eluent solvent to get the **3a**–**c**.

- Methyl 4-((4-methylpiperazin-1-yl)methyl)-3-trifluoromethyl) (**3a**)

The silica gel flash column chromatography was conducted using MeOH:28% NH_3(aq.)_:EA = 0.5:1:98.5 to get the **3a** (1.90 g, 64% yield) as a yellow oil. ^1^H NMR (400 MHz, CDCl_3_) δ 8.28 (s, 1H), 8.15 (d, *J* = 8.0 Hz, 1H), 7.91 (d, *J* = 8.0 Hz, 1H), 3.93 (s, 3H), 3.69 (s, 2H), 2.51–2.45 (m, 8H), 2.29 (s, 3H); ^13^C NMR (100 MHz, CDCl_3_) δ 166.09, 143.52, 132.82, 130.63, 129.09, 129.07 (q, *J* = 31 Hz), 127.32 (q, *J* = 5.9 Hz), 124.14 (d, *J* = 272 Hz), 58.20 (d, *J* = 1.5 Hz), 55.38, 53.44, 52.55, 46.25.

- Methyl 4-((4-methyl-1,4-diazepan-1-yl)methyl)-3-(trifluoromethyl)benzoate (**3b**)

The silica gel flash column chromatography was conducted using MeOH:28% NH_3(aq.)_: EA = 3:1:96 to get the **3c** (1.05 g, 83% yield) as a yellow oil. H NMR (400 MHz, CDCl_3_) δ 8.27 (s, 1H), 8.16 (dd, *J* = 8.1, 1.1 Hz, 1H), 7.97 (d, *J* = 8.1 Hz, 1H), 3.93 (s, 3H), 3.93 (s, 2H), 2.75–2.72 (m, 6H), 2.68–2.65 (m, 2H), 2.40 (s, 3H), 1.87 (quintet, *J* = 6.0 Hz, 2H); ^13^C NMR (100 MHz, CDCl_3_) δ 165.93, 144.34, 132.67, 130.53, 128.88, 128.67 (q, *J* = 31 Hz), 127.16 (q, *J* = 5.9 Hz), 124.00 (q, *J* = 272 Hz), 58.38, 58.36, 58.29, 56.63, 54.66, 54.58, 52.38, 46.86, 27.37.

- Methyl 4-((4-ethylpiperazin-1-yl)methyl)-3-(trifluoromethyl)benzoate (**3c**)

The silica gel flash column chromatography was conducted using hexane:ethyl acetate = 1:1 to obtain **3b** (1.59 g, 89% yield) as a yellow oil. ^1^H NMR (400 MHz, CDCl_3_) δ 8.29 (s, 1H), 8.16 (d, J = 8.0 Hz, 1H), 7.92 (d, J = 8.4 Hz, 1H), 3.94 (s, 3H), 3.70 (s, 2H), 2.53–2.41 (m, 10H), 1.09 (t, *J* = 7.2 Hz, 3H); ^13^C NMR (100 MHz, CDCl_3_) δ 166.05, 143.51, 132.76, 130.60, 129.02 (q, *J* = 31 Hz), 129.01, 127.26 (q, *J* = 5.9 Hz), 124.09 (q, *J* = 272 Hz), 58.19, 53.42, 53.00, 52.50, 52.44, 12.16.

#### 4.2.4. General Procedure for the Synthesis of Benzoic Acid (**4a**–**c**)

A suspension of **3a**, **3b**, or **3c** (1.0 eq.) in anhydrous methanol (20 mL) was cooled in an ice bath. 2.0 N NaOH (aq.) (25 mL) was added dropwise for 15 min. The reaction mixture was stirred and heated at 60 °C overnight, then cooled down. The solvent was evaporated under reduced pressure, then 1 N HCl (aq.) was added dropwise to the residue in the ice bath until a slightly acidic pH = 5–6 value. The solvent was evaporated under reduced pressure, then methanol (10 mL) and DCM (10 mL) were added. The precipitate was filtered, and the filtrate was evaporated to obtain the **4a**–**c** as a light yellow (**4a** and **4b**) or white solid (**4c**) produt.

- 4-((4-methylpiperazin-1-yl)methyl)-3-(trifluoromethyl)benzoic acid (**4a**)

94% yield; ^1^H NMR (400 MHz, Methanol) δ 8.16 (s, 1H), 8.03 (d, *J* = 8.0 Hz, 1H), 7.70 (d, *J* = 8.4 Hz, 1H), 3.66 (s, 2H), 3.10 (br. s, 4H), 2.70 (s, 3H), 2.63 (br. s, 4H); ^13^C NMR (100 MHz, methanol) δ 172.56, 140.36, 137.50, 133.98, 131.78, 129.45 (q, *J* = 30 Hz), 128.07 (q, *J* = 5.6 Hz), 125.92 (q, *J* = 272 Hz), 58.50, 54.94, 51.24, 43.72.

- 4-((4-methyl-1,4-diazepan-1-yl)methyl)-3-(trifluoromethyl)benzoic acid (**4b**)

100% yield; ^1^H NMR (400 MHz, methanol) δ 8.27 (s, 1H), 8.15 (d, *J* = 8.0 Hz, 1H), 7.88 (d, *J* = 8.0 Hz, 1H), 3.90 (s, 2H), 3.43 (t, *J* = 5.4 Hz, 2H), 3.28 (methanol-*d_4_* overlap, 2H), 2.91–2.89 (m, 5H), 2.79 (t, *J* = 6.2 Hz, 2H), 2.07 (quintet, *J* = 5.8 Hz, 2H); ^13^C NMR (100 MHz, methanol-) δ 170.60, 142.70, 134.80, 134.13, 131.96, 129.40 (q, *J* = 30 Hz), 128.03 (q, *J* = 5.8 Hz), 125.74 (d, *J* = 272 Hz), 59.22, 58.44, 56.35, 55.01, 50.96, 49.88, 45.06, 25.70.

- 4-((4-ethylpiperazin-1-yl)methyl)-3-(trifluoromethyl)benzoic acid (**4c**)

76% yield; 1H NMR (400 MHz, Methanol) δ 7.92 (s, 1H), 7.86 (d, *J* = 8.0 Hz, 1H), 7.59 (d, *J* = 8.4 Hz, 1H), 3.50 (s, 2H), 3.03–2.90 (m, 6H), 2.51 (br. s, 4H), 1.07 (t, *J* = 7.4 Hz, 3H); 13C NMR (100 MHz, methanol) δ 172.54, 140.10, 137.85, 133.89, 131.68, 129.99, 129.44 (q, *J* = 30 Hz), 128.04 (q, *J* = 6.0 Hz), 125.92 (d, *J* = 271 Hz), 58.54, 52.99, 52.85, 51.38, 9.82.

##### 4.2.5. 2-Chloro-6-methoxyquinoline (**9′**)

A solution of 6-methoxyquinoline (5.0 g, 30.8 mmol) in anhydrous DCM (20.0 mL) was cooled in an ice bath, then *m*-CPBA (77%, 10.4 g, 46.2 mmol) was added. After the reaction mixture was stirred at room temperature for 3 h, saturated NaHCO_3_ aqueous solution was added to render the solution basic pH = 8. The aqueous layer was extracted with ethyl acetate (20 × 3 mL), and the combined organic layers were washed with brine, dried over anhydrous Na_2_SO_4_, and the solvent was evaporated under reduced pressure to obtain a residue for the next step. After the residue was cooled in an ice bath, POCl_3_ (9.0 mL) was added, heated at 110 °C, and stirred for 1 h. To the reaction mixture, ammonium hydroxide solution was added to alkalinize (pH = 8), and the aqueous layer was extracted with DCM (20 × 3 mL). The combined organic layers were washed with brine solution, dried over anhydrous Na_2_SO_4_, and the solvent was evaporated under reduced pressure to afford a residue which was purified by silica gel flash column chromatography (Hexane:DCM = 1:3) to obtain the **9′** (2.42 g, 41% yield) as a yellow solid. ^1^H NMR (400 MHz, CDCl_3_) δ 7.99 (d, *J* = 8.6 Hz, 1H), 7.91 (d, *J* = 9.2 Hz, 1H), 7.38 (dd, *J* = 9.2, 2.8 Hz, 1H), 7.33 (d, *J* = 8.6 Hz, 1H), 7.06 (d, *J* = 2.8 Hz, 1H), 3.92 (s, 3H); ^13^C NMR (100 MHz, CDCl_3_) δ 158.07, 148.00, 143.78, 137.61, 129.90, 127.89, 123.04, 122.46, 105.25, 55.59.

##### 4.2.6. 4-Fluoro-N^1^-(5-methoxyquinolin-2-yl)benzene-1,3-diamine (**12′**)

A mixture of 2-chloro-5-methoxyquinoline (300 mg, 1.55 mmol) and 4-fluoro-3-nitroaniline (242 mg, 1.55 mmol) was heated at 160 °C. After 3 h, the reaction mixture was cooled, then anhydrous methanol (20.0 mL) and palladium on carbon (10%, 33.0 mg, 0.03 mmol) were added and stirred under H_2_ atmosphere at room temperature. After 12 h, the reaction mixture was filtered over celite, and distilled water was added to the filtrate. The aqueous layer was extracted with ethyl acetate (20 × 3 mL), and the combined organic layer was washed with brine, dried over anhydrous Na_2_SO_4_, and evaporated under reduced pressure to afford a residue which was purified by silica gel flash column chromatography (hexane:ethyl acetate = 3:1, then switching to 1:1) to obtain the **12′** (335 mg, 69% yield) as a dark brown color solid. ^1^H NMR (400 MHz, CDCl_3_) δ 8.32 (d, *J* = 8.6 Hz, 1H), 7.51 (t, *J* = 7.6 Hz, 1H), 7.35 (d, *J* = 7.7 Hz, 1H), 6.96–6.88l (m, 3H), 6.68 (d, J = 7.7 Hz, 2H), 3.95 (s, 3H); ^13^C NMR (100 MHz, CDCl_3_) δ 155.54, 155.05, 148.39 (d, J = 234 Hz), 147.80, 136.21 (d, *J* = 2.6 Hz), 135.11 (d, *J* = 14 Hz), 132.98, 130.17, 118.27, 115.60 (d, *J* = 20 Hz), 115.57, 111.65 (d, *J* = 6.7 Hz), 110.26 (d, *J* = 3.3 Hz), 102.03, 55.63.

#### 4.2.7. N^1^-(6-Methoxyquinolin-2-yl)benzene-1,3-diamine (**13**)

A mixture of **9′** (500 mg, 2.58 mmol) and 3-nitroaniline (357 mg, 2.58 mmol) was heated at 160 °C overnight. After 3 h, the reaction mixture was cooled, then anhydrous MeOH (20.0 mL) and palladium on carbon (10%, 137 mg, 0.129 mmol) were added and stirred under H_2_ atmosphere at room temperature. After 22 h, the reaction mixture was filtered over celite, and distilled water was added to the filtrate. The aqueous layer was extracted with ethyl acetate (20 × 3 mL), and the combined organic layer was washed with brine, dried over anhydrous Na_2_SO_4_, and filtered. The solvent was evaporated under reduced pressure to afford a residue which was purified by silica gel flash column chromatography (hexane:ethyl acetate = 3:1, then switching to 1:1) to obtain the **13** (552 mg, 81% yield) as a yellow color solid. ^1^H NMR (400 MHz, CDCl_3_) δ 7.79 (d, *J* = 9.0 Hz, 1H), 7.63 (d, *J* = 9.1 Hz, 1H), 7.21 (dd, *J* = 9.1, 2.8 Hz, 1H), 7.07 (t, *J* = 7.9 Hz, 1H), 7.02 (d, *J* = 9.0 Hz, 1H), 6.95 (d, *J* = 2.8 Hz, 1H), 6.87 (t, *J* = 2.1 Hz, 1H), 6.38 (dd, *J* = 7.6, 1.8 Hz, 1H), 3.84 (s, 3H), 2.81 (br. s, 2H); ^13^C NMR (100 MHz, CDCl_3_) δ 155.44, 153.27, 147.26, 142.80, 141.35, 136.83, 129.93, 127.46, 124.47, 121.37, 112.14, 110.77, 110.05, 107.07, 106.30, 55.47.

#### 4.2.8. General Procedure for Synthesis **14a**–**c**, **15a**, **16a**, and **16b**

To a solution of **12**, **12′** or **13** (1.0 eq.), **4a**–**c** (1.1 eq.), and HATU (1.5 eq.) in anhydrous DMF (1.0 mL), DIPEA (4.0 eq.) was added, then the reaction mixture was stirred at room temperature overnight. The reaction was quenched by adding a saturated NaHCO_3_ aqueous solution. The aqueous layer was extracted with ethyl acetate (20 × 3 mL), then the combined organic layer was washed with brine, dried over anhydrous Na_2_SO_4_, and filtered. The solvent was evaporated under reduced pressure to afford a residue which was purified by silica gel flash column chromatography using proper eluent to obtain the desired **14a–c**, **15a**, **16a,** and **16b**.

- 4-((4-methylpiperazin-1-yl)methyl)-N-(3-((5-methoxyquinolin-2-yl)amino)phenyl)-3-(trifluoromethyl)benzamide (**14a**)

The silica gel flash column chromatography was conducted using MeOH:DCM = 1:19 eluent to obtain the desired **14a** (116 mg, 80% yield) as a light yellow solid. ^1^H NMR (400 MHz, CDCl_3_) δ 8.52 (s, 1H), 8.29 (d, *J* = 9.2 Hz, 1H), 8.13 (s, 1H), 8.02–7.99 (m, 2H), 7.88 (d, *J* = 8.0 Hz, 1H), 7.47–7.27 (m, 6H), 6.90 (d, *J* = 9.2 Hz, 1H), 6.64 (d, *J* = 7.6 Hz, 1H), 3.94 (s, 3H), 3.67 (s, 2H), 2.50 (br. s, 8H), 1.92 (s, 3H); ^13^C NMR (100 MHz, CDCl_3_) δ 170.94, 164.62, 155.46, 154.38, 148.46, 142.05, 141.16, 138.47, 133.69, 132.52, 130.77, 130.30, 129.81, 129.68, 129.17, 125.32, 124.83 (d, *J* = 5.5 Hz), 119.19, 116.24, 115.94, 114.61, 111.88, 110.95, 102.07, 57.92, 55.60, 55.17, 53.17, 46.03; HRMS (ESI-TOF) m/z calcd for C_30_H_31_F_3_N_5_O_2_ [M + H]^+^: 550.2430, found: 550.2427.

- *N*-(3-((5-methoxyquinolin-2-yl)amino)phenyl)-4-((4-methyl-1,4-diazepan-1-yl)methyl)-3-(trifluoromethyl)benzamide (**14b**)

The silica gel flash column chromatography was conducted using MeOH:DCM = 2:98 eluent to obtain the desired **14b** (36.7 mg, 20% yield) as a light yellow solid. ^1^H NMR (400 MHz, methanol) δ 8.37 (s, 1H), 8.32 (d, *J* = 8.8 Hz, 1H), 8.28 (s, 1H), 8.21 (d, *J* = 8.4 Hz, 1H), 8.04 (d, *J* = 8.0 Hz, 1H), 7.56–7.54 (m, 1H), 7.47 (t, *J* = 8.2 Hz, 1H), 7.36–7.32 (m, 3H), 6.98 (d, *J* = 9.2 Hz, 1H), 6.76 (d, *J* = 8.0 Hz, 1H), 3.97 (s, 3H), 3.95 (s, 2H), 3.46 (t, *J* = 5.4 Hz, 2H), 3.35–3.33 (m, 2H), 2.94–2.92 (m, 5H), 2.80 (t, *J* = 6.1 Hz, 2H), 2.07 (quintet, *J* = 5.7 Hz, 2H);^13^C NMR (100 MHz, methanol) δ 167.10, 156.96, 156.24, 149.26, 142.95, 142.89, 140.21, 135.82, 133.36, 132.51, 132.25, 131.04, 130.21, 130.02, 129.86 (d, *J* = 31 Hz), 126.65,119.79, 117.16, 116.78, 116.18, 113.59, 113.36, 103.36, 59.29, 58.63, 56.51, 56.26, 55.07, 51.13, 45.18, 25.93; HRMS (ESI-TOF) m/z calcd for C_31_H_33_F_3_N_5_O_2_ [M + H]^+^: 564.2586, found: 564.2593.

- 4-((4-ethylpiperazin-1-yl)methyl)-N-(3-((5-methoxyquinolin-2-yl)amino)phenyl)-3-(trifluoromethyl)benzamide (**14c**)

The silica gel flash column chromatography was conducted using MeOH:DCM = 3:97 eluent to obtain the desired **14c** (44.7 mg, 38% yield) as a light yellow solid. ^1^H NMR (400 MHz, CDCl_3_) δ 8.31 (d, *J* = 9.0 Hz, 1H), 8.20 (s, 1H), 8.11 (s, 1H), 8.07 (s, 1H), 8.00 (d, *J* = 8.2 Hz, 1H), 7.88 (d, *J* = 8.1 Hz, 1H), 7.48–7.42 (m, 2H), 7.38 (d, *J* = 8.4 Hz, 1H), 7.32–7.28 (m, 2H), 6.92 (d, *J* = 9.1 Hz, 1H), 6.65 (d, *J* = 7.7 Hz, 1H), 3.95 (s, 3H), 3.68 (s, 2H), 2.55–2.44 (m, 10H), 1.11 (t, *J* = 7.2 Hz, 3H); ^13^C NMR (100 MHz, CDCl_3_) δ 164.51, 155.41, 154.22, 148.35, 141.84, 141.09, 138.38, 133.67, 132.50, 130.81, 130.18, 129.79, 129.65, 128.91, 124.87, 119.19, 116.17, 115.90, 114.55, 117.74, 110.96, 102.07, 57.87, 55.56, 52.78, 52.67, 52.24, 11.68; HRMS (ESI-TOF) m/z calcd for C_31_H_33_F_3_N_5_O_2_ [M + H]^+^: 564.2586, found: 564.2587.

- *N*-(2-fluoro-5-((5-methoxyquinolin-2-yl)amino)phenyl)-4-((4-methylpiperazin-1-yl)methyl)-3-(trifluoromethyl)benzamide (**15a**)

The silica gel flash column chromatography was conducted using MeOH:DCM = 5:95 eluent to obtain the desired **15a** (15.2 mg, 9.4% yield) as a light brown solid. ^1^H NMR (400 MHz, CDCl_3_) δ 8.60–8.58 (m, 1H), 8.33 (d, *J* = 9.0 Hz, 1H), 8.16 (s, 1H), 8.09 (br. s, 1H), 8.01–7.95 (m, 2H), 7.65–7.63 (m, 1H), 7.48 (t, *J* = 8.1 Hz, 1H), 7.40 (d, *J* = 8.4 Hz, 1H), 7.12 (dd, *J* = 10.5, 9.0 Hz, 1H), 6.89 (d, *J* = 9.1 Hz, 1H), 6.66 (d, *J* = 7.4 Hz, 1H), 3.95 (s, 3H), 3.72 (s, 2H), 2.55 (br. s, 8H), 2.33 (s, 3H); ^13^C NMR (100 MHz, CDCl_3_) δ 164.13, 164.04, 155.44, 154.26, 148.51 (d, *J* = 238 Hz), 148.13, 142.46, 136.86, 132.22, 132.75, 130.94, 129.98, 129.95, 127.90 (q, *J* = 31 Hz), 126.28, 126.17, 125.01 (d, *J* = 5.9 Hz), 123.86 (d, *J* = 273 Hz), 119.05, 116.36, 115.83, 115.25, 115.05, 113.41, 110.62, 102.12, 57.89, 55.60, 55.09, 53.00, 45.89; HRMS (ESI-TOF) m/z calcd for C_30_H_30_F_4_N_5_O_2_ [M + H]^+^: 536.2274, found: 536.2278.

- *N*-(3-((6-methoxyquinolin-2-yl)amino)phenyl)-4-((4-methylpiperazin-1-yl)methyl)-3-(trifluoromethyl)benzamide (**16a**)

The silica gel flash column chromatography was conducted using MeOH:ethyl acetate = 5:95 eluent to obtain the desired **16a** (49.8 mg, 27% yield) as a light brown solid. ^1^H NMR (400 MHz, CDCl_3_) δ 8.23 (s, 1H), 8.10 (s, 1H), 8.02 (s, 1H), 7.97 (d, *J* = 8.1 Hz, 1H), 7.88 (d, *J* = 8.2 Hz, 1H), 7.78 (d, *J* = 8.9 Hz, 1H), 7.70 (d, *J* = 9.1 Hz, 1H), 7.38 (d, *J* = 7.8 Hz, 1H), 7.28–7.20 (m, 4H), 7.03 (br. s, 1H), 6.94 (d, *J* = 2.8 Hz, 1H), 6.91 (d, *J* = 8.9 Hz, 1H), 3.86 (s, 3H), 3.67 (s, 2H), 2.50 (br. s, 8H), 2.30 (s, 3H); ^13^C NMR (100 MHz, CDCl_3_) δ 164.65, 155.69, 152.47, 142.72, 142.02, 141.41, 138.38, 136.84, 133.66, 130.77, 130.23, 129.67, 129.02 (q, *J* = 31 Hz), 128.00, 124.81 (q, *J* = 5.8 Hz), 124.65, 123.93 (q, *J* = 273 Hz), 121.41, 115.85, 114.30, 112.66, 111.43, 106.27, 57.90, 55.51, 55.13, 53.09, 45.97; HRMS (ESI-TOF) m/z calcd for C_30_H_31_F_3_N_5_O_2_ [M + H]^+^: 550.2430, found: 550.2436.

- N-(3-((6-methoxyquinolin-2-yl)amino)phenyl)-4-((4-methyl-1,4-diazepan-1-yl)methyl)-3-(trifluoromethyl)benzamide (**16b**)

The silica gel flash column chromatography was conducted using MeOH:DCM = 10:90 eluent to obtain the desired **16b** (108 mg, 44% yield) as a light brown solid. ^1^H NMR (400 MHz, CDCl_3_) δ 8.24 (s, 1H), 8.09 (s, 1H), 8.05 (br. s, 1H), 7.96 (br. s, 2H), 7.78 (d, *J* = 8.9 Hz, 1H), 7.70 (d, *J* = 9.1 Hz, 1H), 7.40 (d, *J* = 7.7 Hz, 1H), 7.28–7.20 (m, 4H), 6.94 (d, *J* = 2.7 Hz, 1H), 6.91 (d, *J* = 8.8 Hz, 2H), 3.86 (s, 3H), 3.80 (s, 2H), 2.72–2.60 (m, 8H), 2.37 (s, 3H), 1.82 (quintet, *J* = 5.9 Hz, 2H); ^13^C NMR (100 MHz, CDCl_3_) δ 164.70, 155.65, 152.49, 143.22, 142.93, 141.51, 138.39, 136.70, 133.52, 130.75, 130.18, 129.64, 128.73 (d, *J* = 31 Hz), 128.19, 124.80 (d, *J* = 5.8 Hz), 124.69, 123.97 (q, *J* = 273 Hz), 121.34, 115.75, 114.20, 112.74, 111.34, 106.25, 58.27, 58.20, 56.73, 55.51, 54.98, 54.59, 47.01, 27.60; HRMS (ESI-TOF) *m*/*z* calcd for C_31_H_33_F_3_N_5_O_2_ [M + H]^+^: 564.2586, found: 564.2597.

#### 4.2.9. General Procedure for Synthesis **17a**,**b** and **18a**,**b**

The crude of **14a**–**c** and **16a**,**b** were prepared as starting material. To the crude mixture in an ice bath, 1.0 M BBr_3_ in DCM (6.0 eq.) was added dropwise, then the reaction mixture was stirred at 0 °C for 30 min at room temperature overnight. After the reaction mixture was cooled down, a saturated NaHCO_3_ aqueous solution was added to render the solution basic, pH = 8. The aqueous layer was extracted with ethyl acetate (20 × 3 mL) and the combined organic layer was washed with brine, dried over anhydrous Na_2_SO_4_, and filtered. The solvent was evaporated under reduced pressure; then, the residue was purified by silica gel flash column chromatography using proper eluent to obtain the desired target compounds **17a**,**b** and **18a**,**b**.

- *N*-(3-((5-hydroxyquinolin-2-yl)amino)phenyl)-4-((4-methylpiperazin-1-yl)methyl)-3-(trifluoromethyl)benzamide (**17a**)

The silica gel flash column chromatography was conducted using MeOH:DCM (6:94, then switching to 10:90) eluent to obtain the desired **17a** (97.5 mg, 46% yield) as a brown solid. ^1^H NMR (400 MHz, DMSO) δ 10.55 (s, 1H), 9.56 (s, 1H), 8.36–8.17 (m, 4H), 7.94 (d, *J* = 7.8 Hz, 1H), 7.60–7.43 (m, 4H), 7.23 (d, *J* = 8.0 Hz, 1H), 7.07 (d, *J* = 8.7 Hz, 1H), 6.77 (s, 1H), 3.80 (s, 2H), 3.16 (s, 1H), 3.09–2.91 (m, 6H), 2.81 (s, 3H), 2.46–2.43 (m, 2H); ^13^C NMR (100 MHz, Methanol) δ 167.00, 155.55, 154.85, 144.28, 141.60, 140.76, 140.05, 136.91, 135.53, 133.11, 132.47, 132.22, 130.94, 130.13, 126.89, 126.60 (d, *J* = 5.7 Hz), 119.28, 118.71, 115.80, 115.38, 114.14, 112.45, 108.99, 58.19, 54.99, 51.06, 43.61.; HRMS (ESI-TOF) *m*/*z* calcd for C_29_H_29_F_3_N_5_O_2_ [M + H]^+^: 536.2274, found: 536.2281.

- *N*-(3-((5-hydroxyquinolin-2-yl)amino)phenyl)-4-((4-methyl-1,4-diazepan-1-yl)methyl)-3-(trifluoromethyl)benzamide (**17b**)

The silica gel flash column chromatography was conducted using MeOH:DCM (10:90) eluent to obtain the desired **17b** (19.5 mg, 9.4%) as a yellow solid. ^1^H NMR (400 MHz, methanol) δ 8.38 (d, *J* = 9.2 Hz, 1H), 8.28 (t, *J* =1.9 Hz, 1H), 8.25 (br. s, 1H), 8.19 (d, *J* = 8.2 Hz, 1H), 8.02 (d, *J* = 8.2 Hz, 1H), 7.47 (dt, *J* = 7.6, 1.7 Hz, 1H), 7.41–7.34 (m, 3H), 7.23 (d, *J* = 8.4 Hz, 1H), 6.99 (d, *J* = 9.2 Hz, 1H), 6.69 (d, *J* = 7.5 Hz, 1H), 3.91 (s, 2H), 3.43 (t, *J* = 5.4 Hz, 2H), 3.34 (s, 1H), 3.32 (methanol overlap, 1H), 2.91 (d, *J* = 5.1 Hz, 2H), 2.89 (s, 3H), 2.76 (t, *J* = 6.1 Hz, 2H), 2.05 (quintet, *J* = 5.7 Hz, 2H); ^13^C NMR (100 MHz, methanol) δ 167.07, 155.49, 155.21, 142.91, 141.55, 140.46, 135.57, 135.31, 132.45, 132.21, 132.08, 130.53, 129.79 (d, *J* = 30 Hz), 126.56 (d, *J* = 5.9 Hz), 118.16, 117.36, 116.28, 115.84, 114.64, 112.66, 108.15, 59.18, 58.53, 56.42, 54.99, 51.04, 45.10, 25.83.; HRMS (ESI-TOF) m/z calcd for C_30_H_31_F_3_N_5_O_2_ [M + H]^+^: 550.2430, found: 550.2444.

- *N*-(3-((6-hydroxyquinolin-2-yl)amino)phenyl)-4-((4-methylpiperazin-1-yl)methyl)-3-(trifluoromethyl)benzamide (**18a**)

The silica gel flash column chromatography was conducted using MeOH:DCM (5:95, then switching to 10:90) eluent to obtain the desired **18a** (15.0 mg, 9.4%) as a brown solid. ^1^H NMR (400 MHz, methanol) δ 8.23 (s, 1H), 8.10 (s, 1H), 8.02 (s, 1H), 7.97 (d, *J* = 8.1 Hz, 1H), 7.88 (d, *J* = 8.2 Hz, 1H), 7.78 (d, *J* = 8.9 Hz, 1H), 7.70 (d, *J* = 9.1 Hz, 1H), 7.38 (d, *J* = 7.8 Hz, 1H), 7.28–7.20 (m, 4H), 7.03 (br. s, 1H), 6.94 (d, *J* = 2.8 Hz, 1H), 6.91 (d, *J* = 8.9 Hz, 1H), 3.86 (s, 3H), 3.67 (s, 2H), 2.50 (br. s, 8H), 2.30 (s, 3H); ^13^C NMR (100 MHz, methanol) δ 164.65, 155.69, 152.47, 142.72, 142.02, 141.41, 138.38, 136.84, 133.66, 130.77, 130.23, 129.67, 129.02 (q, *J* = 31 Hz), 128.00, 124.81 (q, *J* = 5.8 Hz), 124.65, 123.93 (q, *J* = 273 Hz), 121.41, 115.85, 114.30, 112.66, 111.43, 106.27, 57.90, 55.51, 55.13, 53.09, 45.97; HRMS (ESI-TOF) m/z calcd for C_29_H_29_F_3_N_5_O_2_ [M + H]^+^: 536.2273, found: 536.2279.

- *N*-(3-((6-hydroxyquinolin-2-yl)amino)phenyl)-4-((4-methyl-1,4-diazepan-1-yl)methyl)-3-(trifluoromethyl)benzamide (**18b**)

The silica gel flash column chromatography was conducted using MeOH:DCM (10:90, then switching to 15:95) eluent to obtain the desired **18a** (19.1 mg, 4.5%) as yellow solid. ^1^H NMR (400 MHz, methanol) δ 8.27 (s, 1H), 8.23–8.22 (m, 2H), 8.08 (d, *J* = 8.2 Hz, 1H), 8.03 (d, *J* = 9.1 Hz, 1H), 7.67 (d, *J* = 9.0 Hz, 1H), 7.45–7.40 (m, 3H), 7.21 (dd, *J* = 9.0, 2.7 Hz, 1H), 7.08 (dd, *J* = 5.9, 3.2 Hz, 2H), 3.96 (s, 2H), 3.48 (t, *J* = 5.3 Hz, 2H), 3.35 (s, 2H), 2.96–2.93 (m, 5H), 2.81 (t, *J* = 6.1 Hz, 2H), 2.09 (quintet, *J* = 5.7 Hz, 2H); ^13^C NMR (100 MHz, Methanol) δ 165.71, 154.11, 151.90, 141.57, 139.44, 139.29, 138.99, 134.13, 131.09, 130.88, 129.41,128.42 (d, *J* = 30 Hz), 125.16 (d, *J* = 5.8 Hz), 124.46, 123.91, 121.47, 117.19, 116.55, 113.66, 113.49, 109.89, 57.80, 57.15, 55.07, 53.60, 49.66, 48.46, 43.76, 24.48; HRMS (ESI-TOF) m/z calcd for C_30_H_31_F_3_N_5_O_2_ [M + H]^+^: 550.2430, found: 550.2441.

### 4.3. In Vitro Kinase Screening

Reaction Biology Corporation (RBC) Kinase HotSpotSM service was utilized for cell-free biochemical kinase evaluation of the target compounds according to the reported assay protocol [46].

### 4.4. Anticancer Screening at NCI

The anticancer screening of target compounds over a full panel of 60 human cancer cell lines was carried out using Sulforhodamine B (SRB) assay at the National Cancer Institute (NCI), Bethesda, Maryland, USA, employing the standard protocol [47].

### 4.5. Molecular Docking

The crystal structure of B-RAF^V600E^ protein in its DFG-out conformation, co-crystal structure with Sorafenib was taken from Protein Data Bank (PDB code: 1UWJ). Homology modeling of C-RAF (Uniprot: p04049) was built from B-RAF^V600E^ structure after sequence alignment using Discovery Studio 2022 (DS). C-RAF homology model was selected with the lowest PDF energy. The protein structure of B-RAF^V600E^ and C-RAF were prepared for docking by employing the protocol “prepare protein” by removing water molecules, and ligands were prepared through protonation at pH 7.4 and energy minimization. The inhibitors were docked into B-RAF^V600E^ and C-RAF models, respectively, using the CDOCKER in DS software.

### 4.6. Molecular Dynamcis Simulation

Molecular dynamics (MD) simulations were conducted using the same crystal structure of the B-RAF^V600E^ and the C-RAF used in molecular docking calculations. Missing residues were additionally modeled using GalaxyFill [48]. The initial systems were prepared using CHARMM-GUI [49]. AMBER20 software [50] was used to carry out MD simulation. The topology and parameters were modeled using ff19SB force field [51] for the protein and the generalized AMBER force field (GAFF) [52] for the ligands. Each simulation system was solvated using TIP3P water model, and extra NaCl ions were added to the box to neutralize the systems. Temperature and pressure were kept at 300 K and 1 atm, respectively. A SHAKE algorithm was used to constrain all hydrogen atom bonds [53]. Long-range electrostatic interactions were calculated using the particle mesh Ewald (PME) method [54]. Each system was first minimized using 500 steps of steepest descent, and another 20 ps simulation was performed for the equilibration of the system at 300 K in the NVT ensemble. Then, 10 ns equilibration runs were performed to fully relax the systems in NPT ensemble. Finally, we ran a production run for 50 ns and 120 ns (for Figure S6), and last, 20 ns was used for the MM-GBSA calculation.

For MM-GBSA analyses, snapshots were taken every 100 ps of 20 ns MD production runs, resulting in two hundred snapshots per compound. The energies were obtained using the MM_GBSA module of Amber20 [55].

## Figures and Tables

**Table 1:** In vitro kinase inhibitory activity (IC_50_, µM) of the lead compound **VII** and target compounds against B-RAF^V600E^ and C-RAF ^a,b,c^.

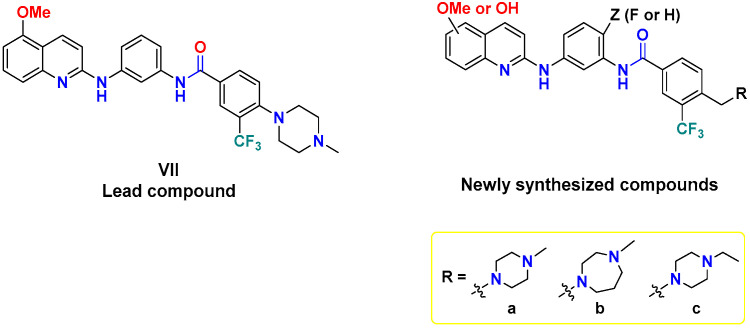						
Compound No.	Substitution Position of Quinoline				IC_50_ (µM)	
	5-	6-	Z	R	B-RAF^V600E^	C-RAF
**VII**	-	-	-	-	1.46 ^b^	0.370 ^b^
**14a**	OMe	H	H	a	0.498 ± 0.0358	0.151 ± 0.00235
**14b**	OMe	H	H	b	0.355 ± 0.0108	0.192 ± 0.0006
**14c**	OMe	H	H	c	0.619 ± 0.00270	0.182 ± 0.0152
**15a**	OMe	H	F	a	0.314 ± 0.0229	0.161 ± 0.00157
**16a**	H	OMe	H	a	0.193 ± 0.00760	0.103 ± 0.00265
**16b**	H	OMe	H	b	0.308 ± 0.0149	0.164 ± 0.00605
**17a**	OH	H	H	a	0.137 ± 0.0132	0.0950 ± 0.0173
**17b**	OH	H	H	b	**0.128 ± 0.00005**	**0.0653 ± 0.0006**
**18a**	H	OH	H	a	**0.114 ± 0.00495**	**0.0676 ± 0.00510**
**18b**	H	OH	H	b	0.227 ± 0.00355	0.161 ± 0.0301
**GW5074**	-	-	-	-	0.00599 ± 0.000727	0.00723 ±0.000159

^a^ Compounds were tested in a 10-dose singlicate IC_50_ mode with 3-fold serial dilution starting at 10, 20, or 100 µM, and the reactions were carried out at 10 µM ATP. ^b^ Data were taken from ref [35]. ^c^ Bold value means a value with the most significant degree of inhibition.

**Table 2:** In vitro kinase inhibitory activity (IC_50_, µM) of the most potent compounds, **17b** and **18a**, against B-RAF^V600E/V600K^ and C-RAF ^a,b,c^.

	IC_50_ (µM)				
	B-RAF^V600E^		C-RAF		B-RAF^V600K^
ATP conc.	10 µM	1 µM	10 µM	1 µM	10 µM
**17b**	0.128 ± 0.001 ^b^	NT	0.0653 ± 0.001 ^b^	NT	0.0616 ± 0.001
**18a**	0.114 ± 0.005 ^b^	0.0843 ± 0.009	0.0676 ± 0.005 ^b^	0.0293 ± 0.009	<0.00508

^a^ Compounds were tested in a 10-dose singlicate IC_50_ mode with 3-fold serial dilution starting at 10, 20, or 100 µM, and the reactions were carried out at 1 or 10 µM ATP. ^b^ Data were taken from Table 1. ^c^ NT: not tested.

**Table 3:** GI_50_ (μM) of compounds **14a–c**, **15a** and **16a** against NCI-60 human tumor cell lines in five-dose test ^a,b^.

Cancer Type	Cell Lines	GI_50_ (μM)				
		14a	14b	14c	15a	16a
Leukemia	CCRF-CEM	2.10	**1.62**	**1.97**	9.71	2.15
	HL-60(TB)	**1.22**	**1.65**	**1.57**	6.52	5.46
	K-562	2.70	**1.23**	2.11	3.59	**0.882**
	MOLT-4	**1.45**	**1.24**	**1.23**	4.10	**1.63**
	RPMI-8226	2.78	**1.93**	2.24	9.30	3.14
	SR	**1.85**	NT	**1.87**	9.83	**1.31**
Non-Small-Cell Lung (NSCL) Cancer	A549/ATCC	2.44	**1.90**	2.50	12.2	4.36
	EKVX	2.10	**1.72**	4.06	14.7	4.53
	HOP-62	**1.56**	**1.76**	3.67	14.5	6.36
	HOP-92	**1.5**	NT	**1.58**	6.64	**1.36**
	NCI-H226	4.63	**1.94**	5.53	11.1	4.60
	NCI-H23	**1.91**	**1.68**	2.37	15.5	5.06
	NCI-H322M	2.29	2.12	3.50	15.2	6.06
	NCI-H460	**1.71**	**1.76**	**1.47**	15.9	**1.88**
	NCI-H522	**1.79**	**1.76**	**1.70**	16.0	4.88
Colon Cancer	COLO 205	**1.79**	**1.81**	**1.74**	17.2	5.78
	HCC-2998	**1.99**	**1.71**	**1.59**	18.8	5.25
	HCT-116	**1.82**	**1.67**	**1.45**	8.75	2.41
	HCT-15	**1.78**	**1.62**	**1.58**	13.4	1.18
	HT29	**1.86**	**1.66**	**1.76**	3.61	1.32
	KM12	2.13	2.49	2.20	15.4	4.05
	SW-620	**1.73**	**1.75**	**1.45**	16.9	5.89
CNS Cancer	SF-268	**1.69**	**1.77**	2.17	11.1	5.20
	SF-295	**1.77**	**1.74**	**1.52**	17.1	3.41
	SF-539	**1.64**	**1.55**	**1.37**	12.4	2.08
	SNB-19	**1.78**	**1.72**	**1.65**	17.2	6.14
	SNB-75	**1.60**	**1.52**	**1.20**	9.09	**0.684**
	U251	**1.92**	**1.76**	**1.69**	13.4	3.77
Melanoma	LOX IMVI	**1.74**	**1.68**	**1.45**	13.8	1.55
	MALME-3M	**1.65**	**1.72**	**1.44**	11.7	4.62
	M14	**1.76**	**1.81**	**1.47**	13.1	3.63
	MDA-MB-435	**1.68**	**1.80**	**1.36**	15.1	5.73
	SK-MEL-2	3.52	2.15	10.7	16.1	5.98
	SK-MEL-28	**1.71**	**1.68**	**1.56**	15.3	5.33
	SK-MEL-5	**1.62**	**1.71**	**1.45**	15.5	5.10
	UACC-257	**1.98**	**1.86**	**1.51**	16.3	4.35
	UACC-62	**1.75**	**1.81**	**1.53**	16.2	6.45
Ovarian Cancer	IGROV1	2.25	**1.81**	3.09	14.5	6.22
	OVCAR-3	2.15	2.14	**1.80**	14.4	5.89
	OVCAR-4	2.21	2.67	2.89	13.9	4.32
	OVCAR-5	**1.84**	**1.71**	3.28	16.1	6.10
	OVCAR-8	3.09	2.03	6.78	15.8	5.82
	NCI/ADR-RES	2.28	**1.72**	2.28	NT	NT
	SK-OV-3	9.22	4.33	12.6	15.7	5.99
Renal Cancer	786-0	**1.76**	**1.72**	**1.41**	12.5	5.00
	A498	2.00	13.5	11.8	16.9	7.35
	ACHN	2.05	**1.87**	**1.85**	16.5	4.88
	CAKI-1	**1.81**	**1.72**	**1.46**	11.5	4.90
	RXF 393	**1.73**	**1.66**	**1.63**	3.71	**1.75**
	SN12C	**1.71**	**1.63**	3.62	13.4	5.49
	TK-10	2.15	4.91	8.67	16.4	6.52
	UO-31	**1.64**	**1.74**	2.33	12.6	5.68
Prostate Cancer	PC-3	**1.70**	**1.54**	2.14	12.0	2.90
	DU-145	**1.98**	2.19	2.55	12.9	5.24
Breast Cancer	MCF7	**1.57**	**1.59**	**1.50**	12.3	**1.37**
	MDA-MB-231/ATCC	**1.77**	**1.83**	**1.54**	12.1	**1.84**
	HS 578T	**1.84**	2.46	**1.96**	14.1	4.14
	BT-549	7.01	2.06	5.35	10.5	5.58
	T-47D	2.28	5.43	2.11	14.8	4.12
	MDA-MB-468	**1.92**	**1.56**	**1.60**	4.79	**1.56**

^a^ Bold values indicate the GI_50_ under 2.0 μM. ^b^ NT: Not tested.

**Table 4:** GI_50_ (μM) of compounds **17a**, **17b**, **18a**, **18b**, and Sorafenib against NCI-60 human tumor cell lines in five-dose test ^a,b,c^.

Cancer Type	Cell Lines	GI_50_ (μM)				
		17a	17b	18a	18b	Sorafenib ^c^
Leukemia	CCRF-CEM	2.91	2.07	3.48	4.94	2.2
	HL-60(TB)	2.47	2.35	2.58	11.9	**1.6**
	K-562	2.42	2.18	**1.88**	**1.18**	2.8
	MOLT-4	**1.86**	2.11	3.52	2.05	2.9
	RPMI-8226	2.92	2.89	2.65	11.4	**1.5**
	SR	4.39	NT	2.68	**1.89**	3.1
Non-Small-Cell Lung (NSCL) Cancer	A549/ATCC	4.50	3.63	8.70	16.2	2.9
	EKVX	3.75	**1.82**	14.2	16.0	2.5
	HOP-62	3.00	2.03	12.1	17.8	**1.9**
	HOP-92	**1.32**	**1.64**	3.39	10.4	**1.6**
	NCI-H226	19.2	16.7	16.0	13.8	**1.9**
	NCI-H23	2.26	**1.78**	13.9	16.8	**1.8**
	NCI-H322M	3.23	3.27	11.0	16.2	2.8
	NCI-H460	2.19	**1.81**	3.84	18.0	2.3
	NCI-H522	**1.93**	**1.84**	4.82	17.0	2.1
Colon Cancer	COLO 205	**1.99**	**1.83**	10.2	18.5	2.2
	HCC-2998	**1.89**	**1.80**	2.39	16.5	3.0
	HCT-116	2.70	**1.62**	3.93	6.51	**1.7**
	HCT-15	**1.82**	**1.93**	2.09	16.4	2.5
	HT29	2.06	**1.92**	3.37	13.3	2.2
	KM12	2.30	**1.98**	3.16	14.1	**1.6**
	SW-620	2.24	**1.95**	2.30	14.5	2.8
CNS Cancer	SF-268	3.81	2.14	10.4	17.3	2.5
	SF-295	**1.65**	**1.67**	2.33	17.3	**1.6**
	SF-539	**1.67**	**1.70**	**1.98**	11.3	**1.6**
	SNB-19	2.39	2.03	13.8	17.0	3.4
	SNB-75	**1.85**	**1.32**	**0.656**	**1.17**	**3.0**
	U251	2.38	2.07	2.17	14.8	2.1
Melanoma	LOX IMVI	**1.65**	**1.63**	**1.73**	12.1	**1.6**
	MALME-3M	**1.67**	**1.70**	**1.59**	12.6	2.2
	M14	2.21	**1.89**	9.06	12.7	2.2
	MDA-MB-435	**1.78**	**1.78**	8.53	10.4	**1.8**
	SK-MEL-2	**1.92**	**1.81**	13.5	17.2	**1.8**
	SK-MEL-28	**1.77**	**1.82**	**1.79**	13.9	2.6
	SK-MEL-5	**1.68**	**1.72**	2.35	16.1	**1.5**
	UACC-257	**1.92**	**1.60**	7.35	14.7	2.2
	UACC-62	**1.73**	**1.69**	15.4	15.9	**1.7**
Ovarian Cancer	IGROV1	4.70	2.56	16.6	17.2	2.6
	OVCAR-3	3.10	**1.97**	11.2	16.3	2.9
	OVCAR-4	7.39	3.83	11.2	15.0	3.5
	OVCAR-5	3.24	11.4	14.2	15.3	2.9
	OVCAR-8	3.56	2.51	13.2	18.9	3.0
	NCI/ADR-RES	3.45	6.43	NT	NT	2.5
	SK-OV-3	14.4	11.1	16.5	17.6	2.3
Renal Cancer	786-0	2.11	2.06	3.13	16.0	3.4
	A498	17.5	15.3	22.5	17.6	2.3
	ACHN	2.07	**1.92**	7.59	19.7	2.8
	CAKI-1	2.13	**1.79**	11.9	14.1	2.9
	RXF 393	**1.66**	**1.85**	3.55	12.5	3.4
	SN12C	2.34	**1.83**	14.1	17.3	2.4
	TK-10	3.36	2.80	21.4	18.5	4.4
	UO-31	**1.99**	2.04	13.4	15.2	2.6
Prostate Cancer	PC-3	2.53	**1.87**	4.68	13.1	2.0
	DU-145	4.68	3.27	10.5	16.9	3.3
Breast Cancer	MCF7	**1.74**	**1.68**	2.46	9.83	2.8
	MDA-MB-231/ATCC	**1.62**	**1.65**	2.54	13.9	**1.3**
	HS 578T	2.96	**1.93**	4.17	14.8	2.6
	BT-549	15.6	**1.80**	12.2	NT	3.3
	T-47D	10.7	3.46	14.6	15.8	**1.8**
	MDA-MB-468	**1.62**	**1.66**	2.23	8.88	2.0

^a^ Bold values indicate the GI_50_ under 2.0 μM. ^b^ NT: Not tested. ^c^ Data taken from ref [43].

**Table 5:** Calculated binding free energy of compounds **17b** and **18a** against B-RAF^V600E^ and C-RAF.

Compound No.	MMGBSA (kcal/mol)	
	B-RAF^V600E^	C-RAF
**17b**	−33.85 ± 12.05	−70.64 ± 6.96
**18a**	−58.02 ± 9.42	−75.35 ± 6.88

## Data Availability

Data are contained within the article and Supplementary Materials.

## Supplementary Materials

The following supporting information can be downloaded at: https://www.mdpi.com/article/10.3390/ijms24043216/s1, The supporting information associated with this article is available.
