# Supplementary material for: Discovery of New Quinolone-Based Diarylamides as Potent B-RAFV600E/C-RAF Kinase Inhibitors Endowed with Promising In Vitro Anticancer Activity
Source: Int J Mol Sci. 2023 Feb 6;24(4):3216. doi: 10.3390/ijms24043216 (PMC9963398; doi:10.3390/ijms24043216)
Supplement: Supplementary file 1 [file ijms-24-03216-s001.zip › ijms-2101649-supplementary.pdf]

Supporting Information

Discovery of new quinoline-based diarylamides as potent B-RAF^{V600E}/C-RAF kinase inhibitors endowed with promising in vitro anticancer activity

Hyun Ji Kim[1], Jung Woo Park[2], Sangjae Seo[2], Kwang-Hwi Cho[3], Mohammed M. Alanazi[4], Eun-Kyoung Bang[1], Gyochang Keum[1,5*,] and Ashraf K. El-Damasy[1*]

[1] Center for Brain Technology, Brain Science Institute, Korea Institute of Science and Technology (KIST), Seoul 02792, Republic of Korea; hjkim926@kist.re.kr (H.J.K), eunkbang@kist.re.kr (E.-K.B.)
[2] Supercomputing Application Center, Div. of National Supercomputing, Korea Institute of Science and Technology Information, 245, Daehak-ro, Yuseong-gu, Daejeon 34141, Republic of Korea; j.w.park@kisti.re.kr (J.W.P.), sj.seo@kisti.re.kr (S.S.)
[3] School of Systems Biomedical Science, Soongsil University, Seoul, 06978, Republic of Korea; chokh@ssu.ac.kr (K.-H.C.)
[4] Department of Pharmaceutical Chemistry, College of Pharmacy, King Saud University, Saudi Arabia; mmalanazi@ksu.edu.sa
[5] Division of Bio-Medical Science & Technology, KIST School, Korea University of Science and Technology (UST), Seoul, 02792, Republic of Korea
***** Correspondence: gkeum@kist.re.kr (G. Keum), ashraf.el-damasy@kist.re.kr or phkarem2006@gmail.com (A.K.El-Damasy)

Contents **Pages**

1) ^{1}H NMR and ^{13}C NMR spectra 2–11
2) HRMS charts 12–21
3) Chemical structure of GW5074 21
4) NCI single dose data 22–31
5) Molecular docking 32–35
6) Molecular dynamics 36–37

1) ^{1}H NMR and ^{13}C NMR spectra

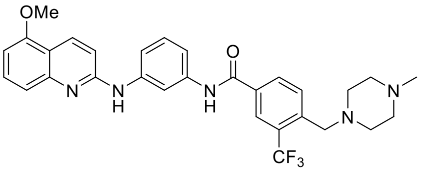

AKA-Vlll-75 (14a)

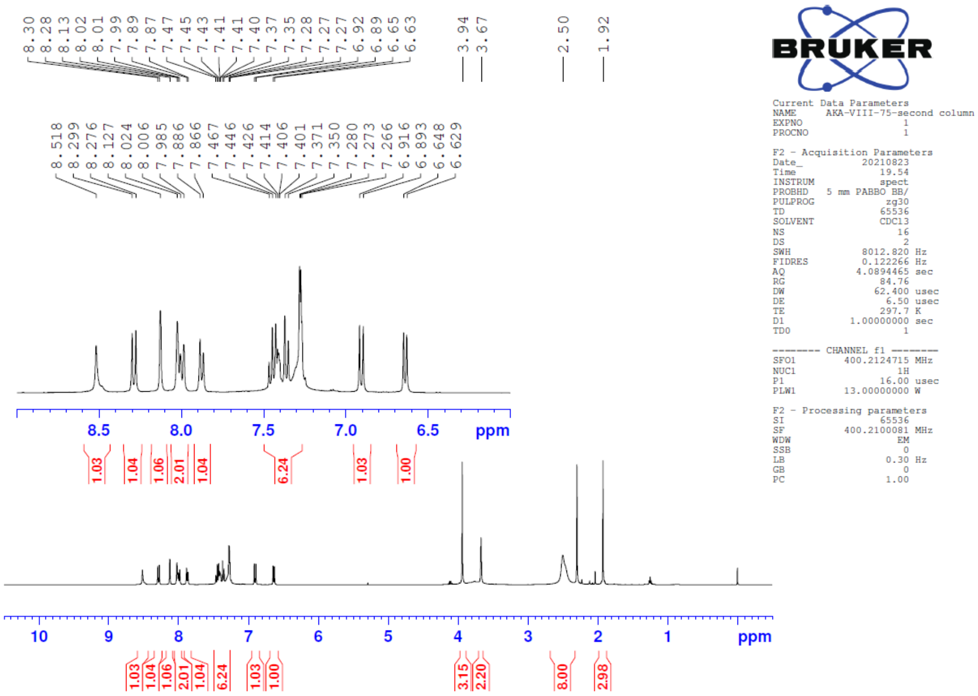

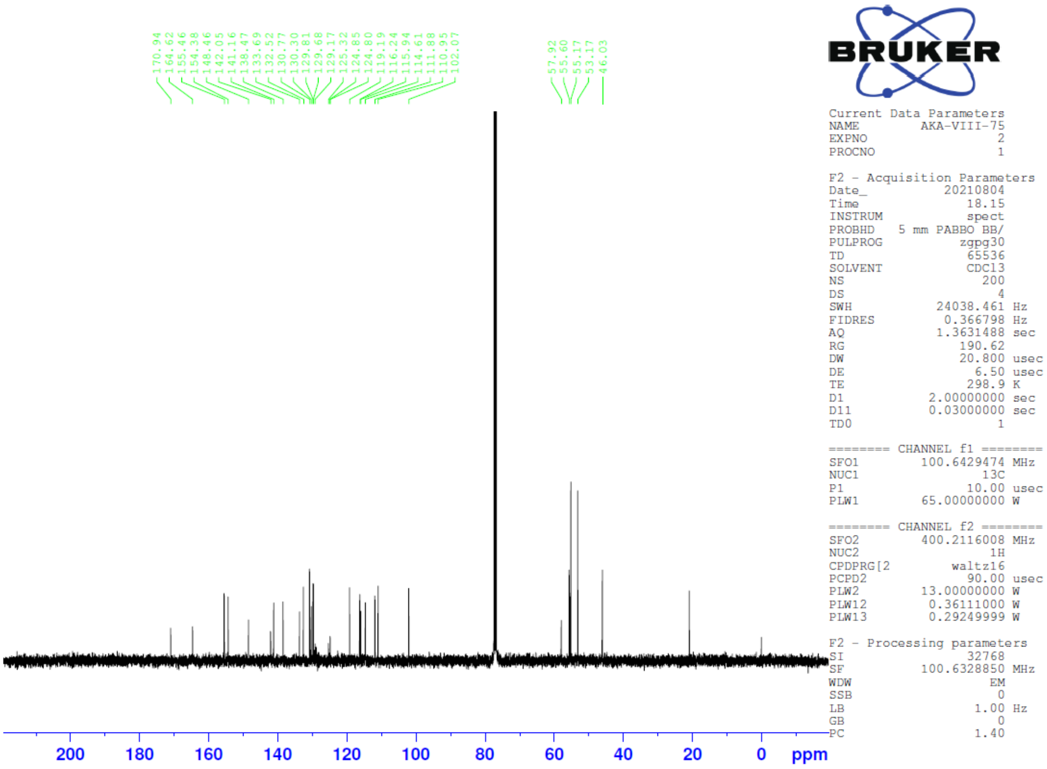

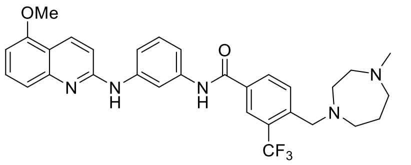

HJ_1-99 = HJ_2-9 (14b)

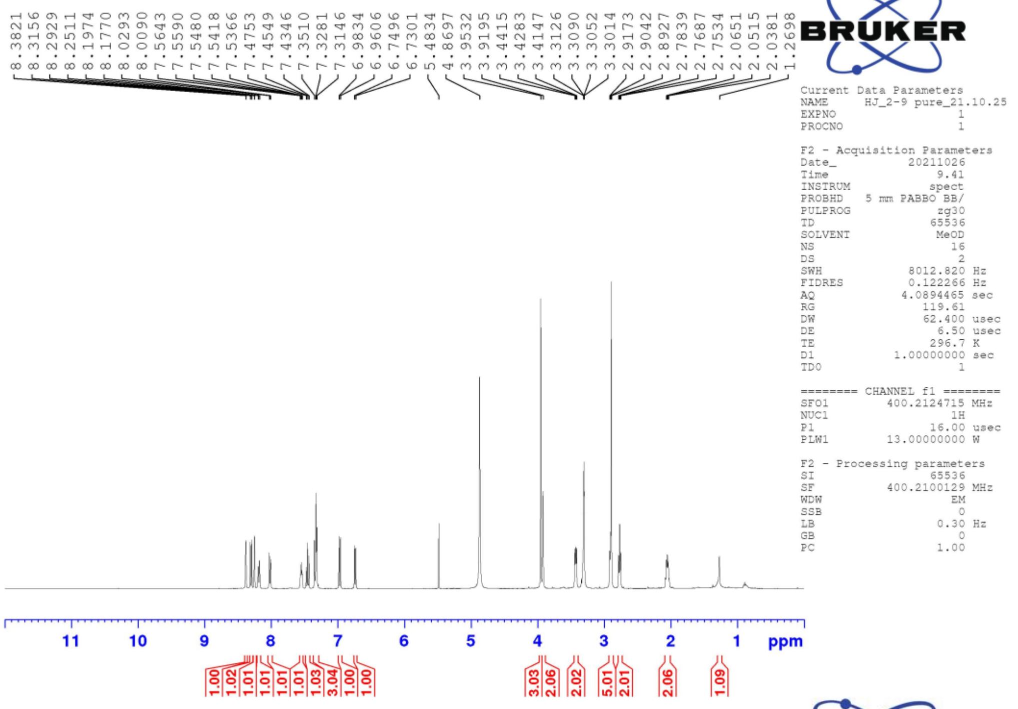

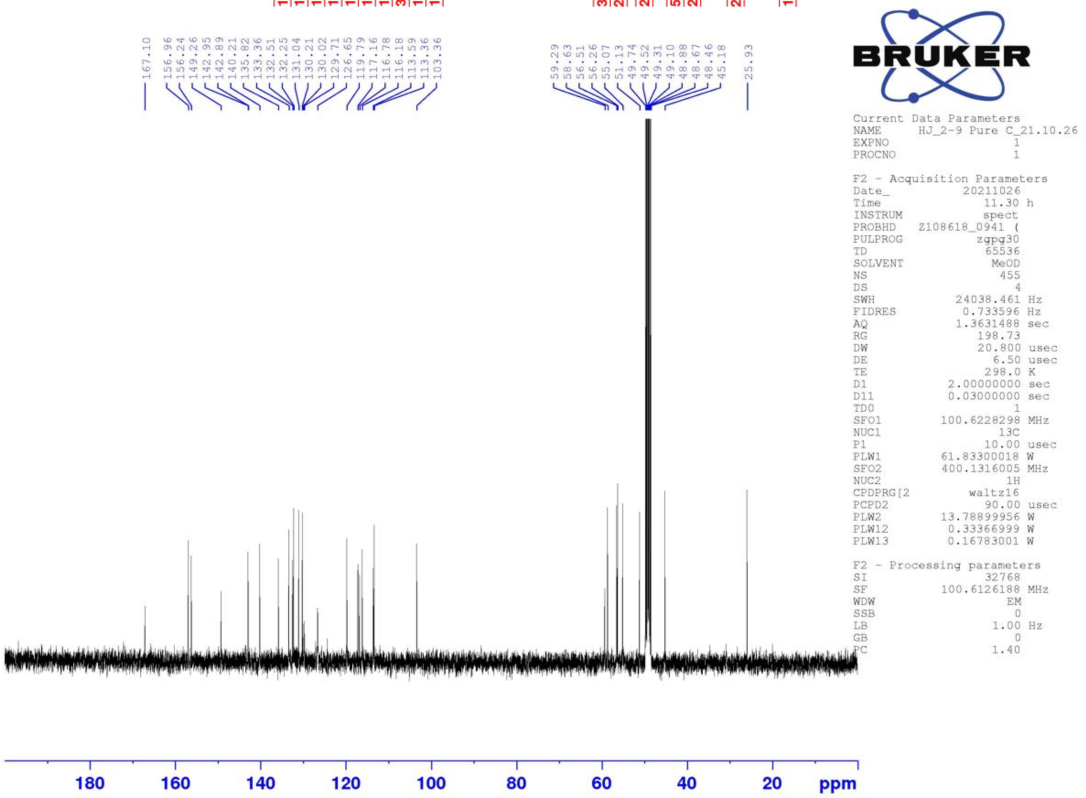

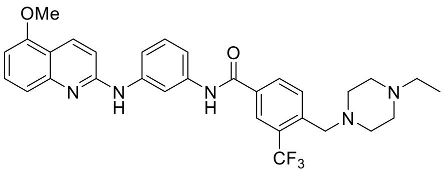
OMe
N
N
H
N
H
O
N
N
CF3

HJ-1-98 (14c)

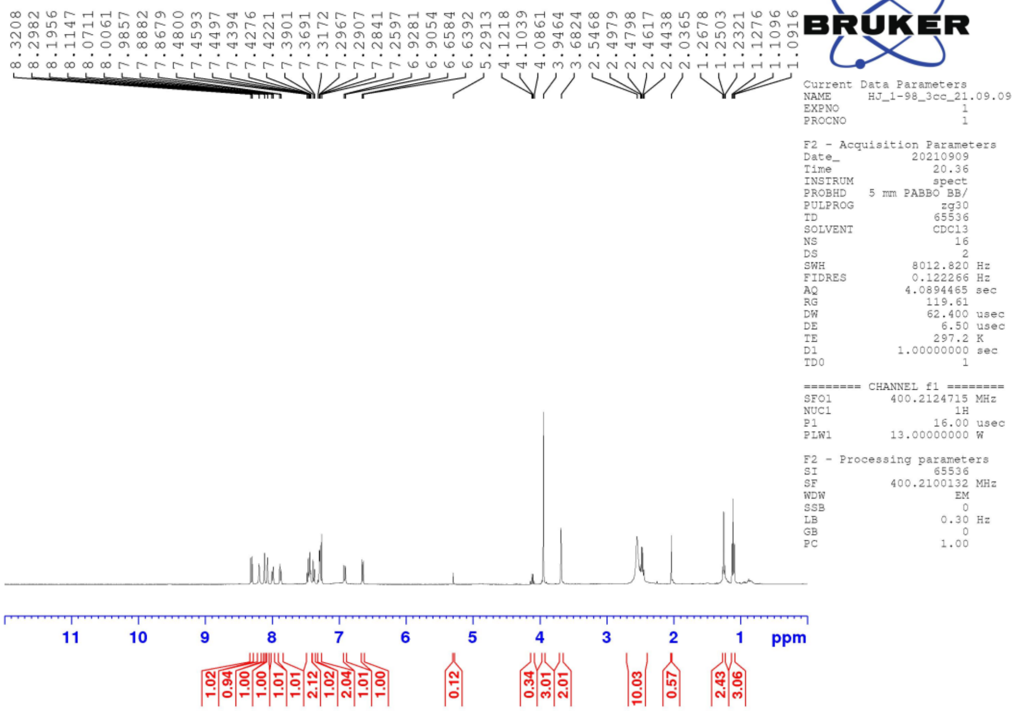
8.3208
8.2982
8.1956
8.1147
8.0711
8.0061
7.9857
7.8882
7.8679
7.4800
7.4593
7.4497
7.4394
7.4276
7.4221
7.3901
7.3691
7.3172
7.2967
7.2907
7.2841
7.2597
6.9281
6.9054
6.6584
6.6392
5.2913
4.1218
4.1039
4.0861
3.9464
3.6824
2.5468
2.4979
2.4798
2.4617
2.4438
2.0365
1.2678
1.2503
1.2321
1.1276
1.1096
1.0916
BRUKER
Current Data Parameters
NAME HJ_1-98_3cc_21.09.09
EXPNO 1
PROCNO 1
F2 - Acquisition Parameters
Date_ 20210909
Time 20.36
INSTRUM spect
PROBHD 5 mm PABBO BB/
PULPROG zg30
TD 65536
SOLVENT CDCl3
NS 16
DS 2
SWH 8012.820 Hz
FIDRES 0.122266 Hz
AQ 4.0894465 sec
RG 119.61
DW 62.400 usec
DE 6.50 usec
TE 297.2 K
D1 1.00000000 sec
TD0 1
======== CHANNEL f1 ========
SFO1 400.2124715 MHz
NUC1 1H
P1 16.00 usec
PLW1 13.00000000 W
F2 - Processing parameters
SI 65536
SF 400.2100132 MHz
WDW EM
SSB 0
LB 0.30 Hz
GB 0
PC 1.00
11 10 9 8 7 6 5 4 3 2 1 ppm
1.02
0.94
1.00
1.00
1.01
1.01
2.12
1.02
2.04
1.01
1.00
0.12
0.34
3.01
2.01
10.03
0.57
2.43
3.06

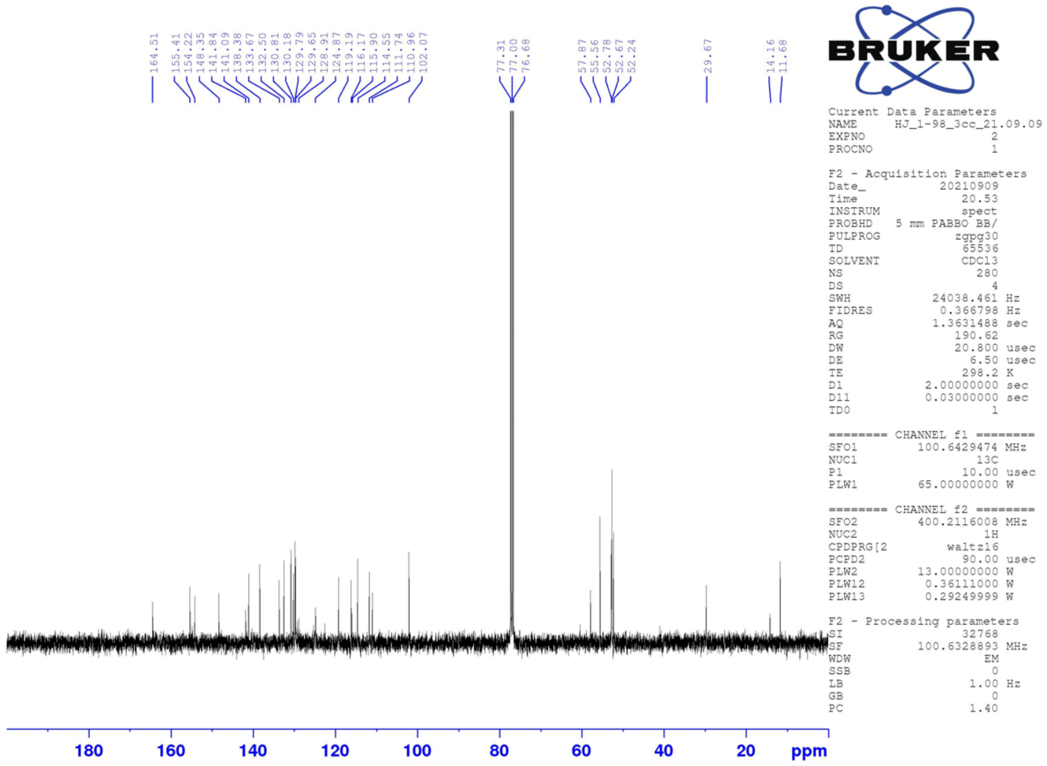
164.51
155.41
154.22
148.35
141.84
141.09
138.38
133.67
132.50
130.81
130.18
129.79
129.65
128.91
124.87
119.19
116.17
115.90
114.55
111.74
110.96
102.07
77.31
77.00
76.68
57.87
55.56
52.78
52.67
52.24
29.67
14.16
11.68
BRUKER
Current Data Parameters
NAME HJ_1-98_3cc_21.09.09
EXPNO 2
PROCNO 1
F2 - Acquisition Parameters
Date_ 20210909
Time 20.53
INSTRUM spect
PROBHD 5 mm PABBO BB/
PULPROG zgpg30
TD 65536
SOLVENT CDCl3
NS 280
DS 4
SWH 24038.461 Hz
FIDRES 0.366798 Hz
AQ 1.3631488 sec
RG 190.62
DW 20.800 usec
DE 6.50 usec
TE 298.2 K
D1 2.00000000 sec
D11 0.03000000 sec
TD0 1
======== CHANNEL f1 ========
SFO1 100.6429474 MHz
NUC1 13C
P1 10.00 usec
PLW1 65.00000000 W
======== CHANNEL f2 ========
SFO2 400.2116008 MHz
NUC2 1H
CPDPRG[2 waltz16
PCPD2 90.00 usec
PLW2 13.00000000 W
PLW12 0.36111000 W
PLW13 0.29249999 W
F2 - Processing parameters
SI 32768
SF 100.6328893 MHz
WDW EM
SSB 0
LB 1.00 Hz
GB 0
PC 1.40
180 160 140 120 100 80 60 40 20 ppm

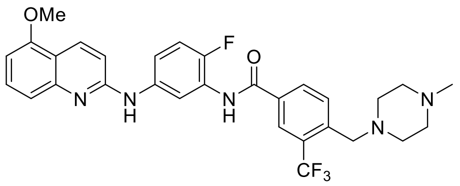
OMe
F
O
N
H
N
H
N
N
N
CF3

HJ_2-32 (15a)

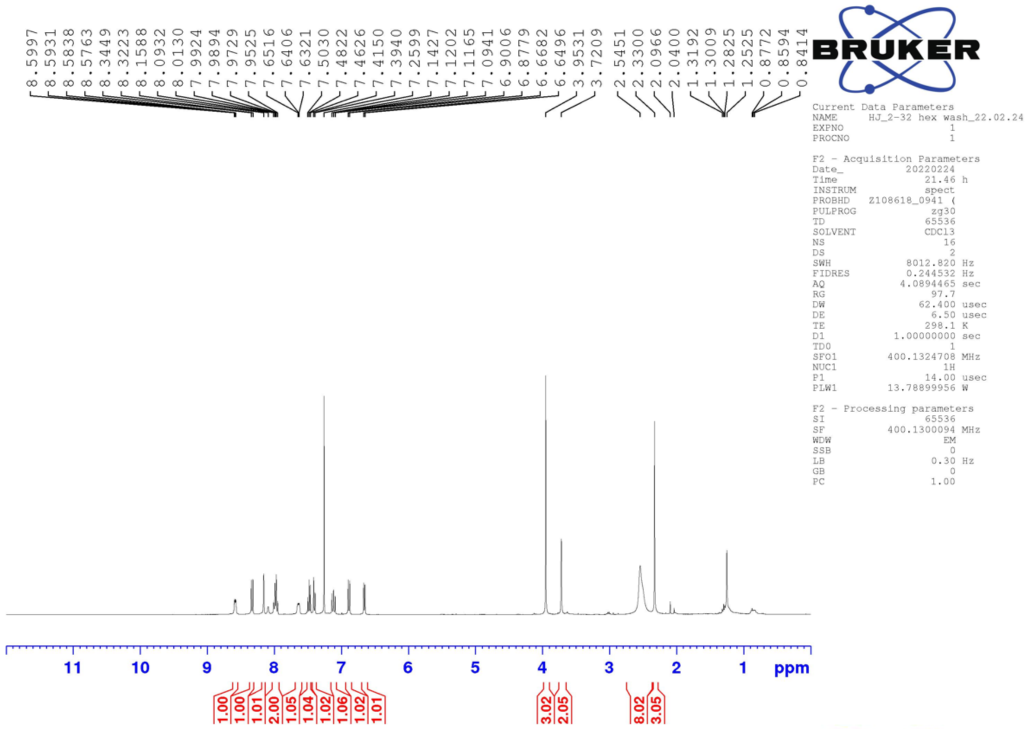
8.5997
8.5931
8.5838
8.5763
8.3449
8.3223
8.1588
8.0932
8.0130
7.9924
7.9894
7.9729
7.9525
7.6516
7.6406
7.6321
7.5030
7.4822
7.4626
7.4150
7.3940
7.2599
7.1427
7.1202
7.1165
7.0941
6.9006
6.8779
6.6682
6.6496
3.9531
3.7209
2.5451
2.3300
2.0966
2.0400
1.3192
1.3009
1.2825
1.2525
0.8772
0.8594
0.8414
BRUKER
Current Data Parameters
NAME HJ_2-32 hex wash_22.02.24
EXPNO 1
PROCNO 1
F2 - Acquisition Parameters
Date_ 20220224
Time 21.46 h
INSTRUM spect
PROBHD Z108618_0941 (
PULPROG zg30
TD 65536
SOLVENT CDCl3
NS 16
DS 2
SWH 8012.820 Hz
FIDRES 0.244532 Hz
AQ 4.0894465 sec
RG 97.7
DW 62.400 usec
DE 6.50 usec
TE 298.1 K
D1 1.00000000 sec
TD0 1
SFO1 400.1324708 MHz
NUC1 1H
P1 14.00 usec
PLW1 13.78899956 W
F2 - Processing parameters
SI 65536
SF 400.1300094 MHz
WDW EM
SSB 0
LB 0.30 Hz
GB 0
PC 1.00
11
10
9
8
7
6
5
4
3
2
1
ppm
1.00
1.00
1.01
2.00
1.05
1.04
1.02
1.06
1.02
1.01
3.02
2.05
8.02
3.05

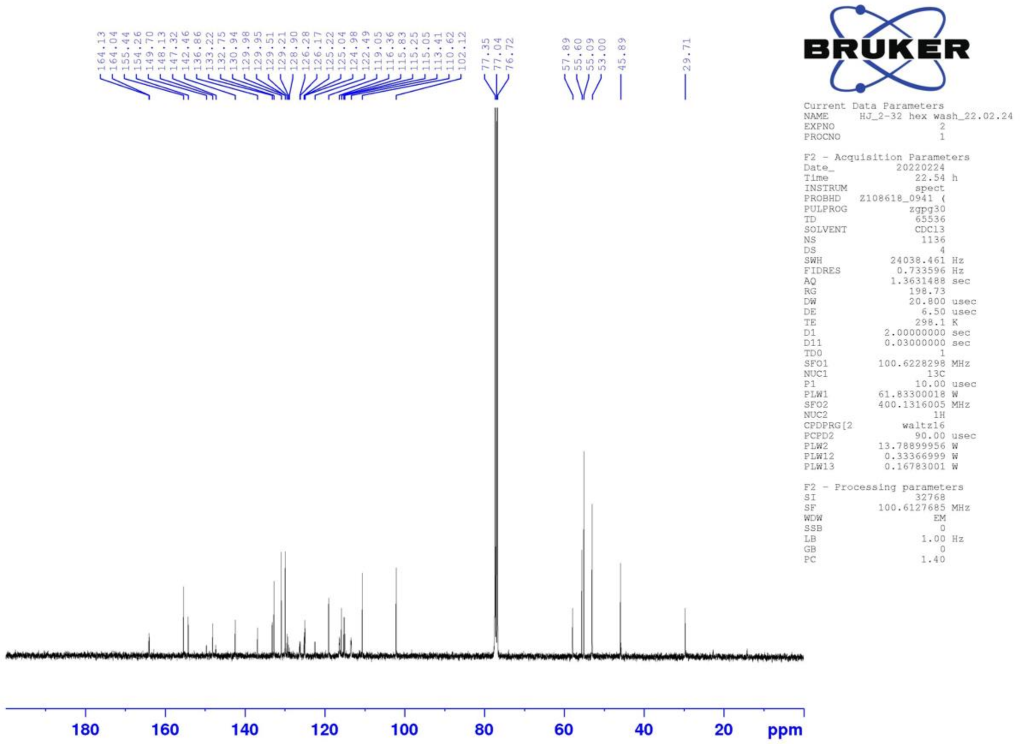
164.13
164.04
155.44
154.26
149.70
148.13
147.32
142.46
136.86
133.22
132.75
130.94
129.98
129.95
129.51
129.21
128.90
126.28
126.17
125.22
125.04
124.98
122.49
119.05
116.36
115.83
115.25
115.05
113.41
110.62
102.12
77.35
77.04
76.72
57.89
55.60
55.09
53.00
45.89
29.71
BRUKER
Current Data Parameters
NAME HJ_2-32 hex wash_22.02.24
EXPNO 2
PROCNO 1
F2 - Acquisition Parameters
Date_ 20220224
Time 22.54 h
INSTRUM spect
PROBHD Z108618_0941 (
PULPROG zgpg30
TD 65536
SOLVENT CDCl3
NS 1136
DS 4
SWH 24038.461 Hz
FIDRES 0.733596 Hz
AQ 1.3631488 sec
RG 198.73
DW 20.800 usec
DE 6.50 usec
TE 298.1 K
D1 2.00000000 sec
D11 0.03000000 sec
TD0 1
SFO1 100.6228298 MHz
NUC1 13C
P1 10.00 usec
PLW1 61.83300018 W
SFO2 400.1316005 MHz
NUC2 1H
CPDPRG[2 waltz16
PCPD2 90.00 usec
PLW2 13.78899956 W
PLW12 0.33366999 W
PLW13 0.16783001 W
F2 - Processing parameters
SI 32768
SF 100.6127685 MHz
WDW EM
SSB 0
LB 1.00 Hz
GB 0
PC 1.40
180
160
140
120
100
80
60
40
20
ppm

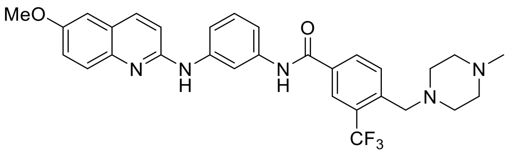
MeO
N
N
H
N
H
O
N
N
CF3

HJ_2-35 (16a)

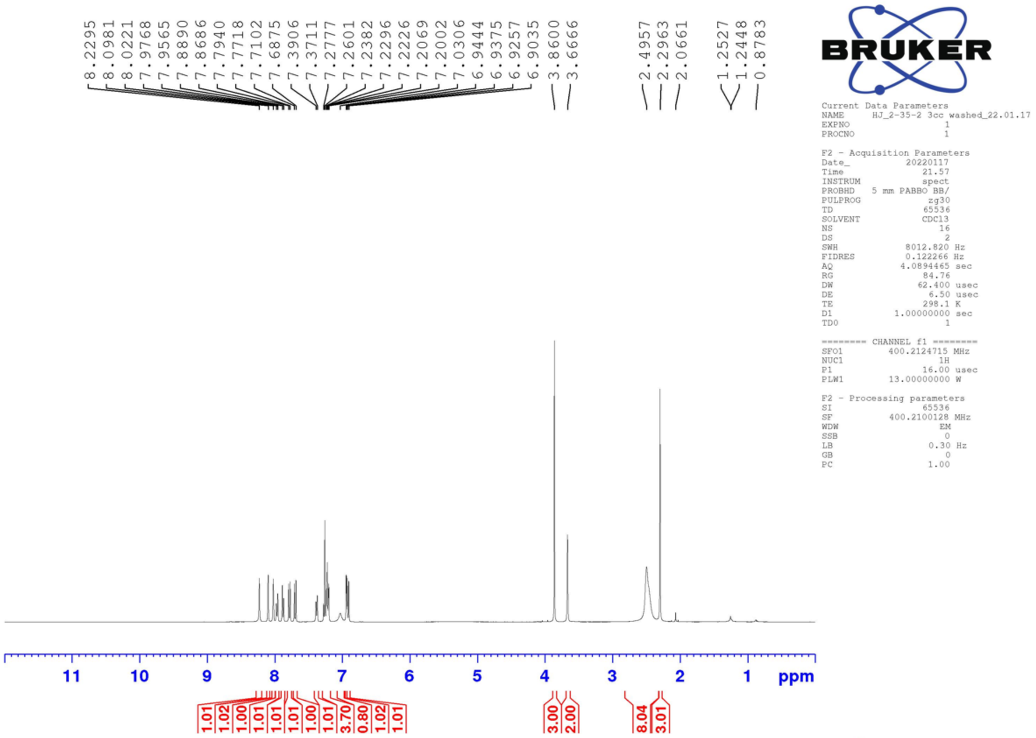
8.2295
8.0981
8.0221
7.9768
7.9565
7.8890
7.8686
7.7940
7.7718
7.7102
7.6875
7.3906
7.3711
7.2777
7.2601
7.2382
7.2296
7.2226
7.2069
7.2002
7.0306
6.9444
6.9375
6.9257
6.9035
3.8600
3.6666
2.4957
2.2963
2.0661
1.2527
1.2448
0.8783
BRUKER
Current Data Parameters
NAME HJ_2-35-2 3cc washed_22.01.17
EXPNO 1
PROCNO 1
F2 - Acquisition Parameters
Date_ 20220117
Time 21.57
INSTRUM spect
PROBHD 5 mm PABBO BB/
PULPROG zg30
TD 65536
SOLVENT CDCl3
NS 16
DS 2
SWH 8012.820 Hz
FIDRES 0.122266 Hz
AQ 4.0894465 sec
RG 84.76
DW 62.400 usec
DE 6.50 usec
TE 298.1 K
D1 1.00000000 sec
TD0 1
======== CHANNEL f1 ========
SFO1 400.2124715 MHz
NUC1 1H
P1 16.00 usec
PLW1 13.00000000 W
F2 - Processing parameters
SI 65536
SF 400.2100128 MHz
WDW EM
SSB 0
LB 0.30 Hz
GB 0
PC 1.00
11
10
9
8
7
6
5
4
3
2
1
ppm
1.01
1.02
1.00
1.01
1.01
1.01
1.00
1.01
3.70
0.80
1.02
1.01
3.00
2.00
8.04
3.01

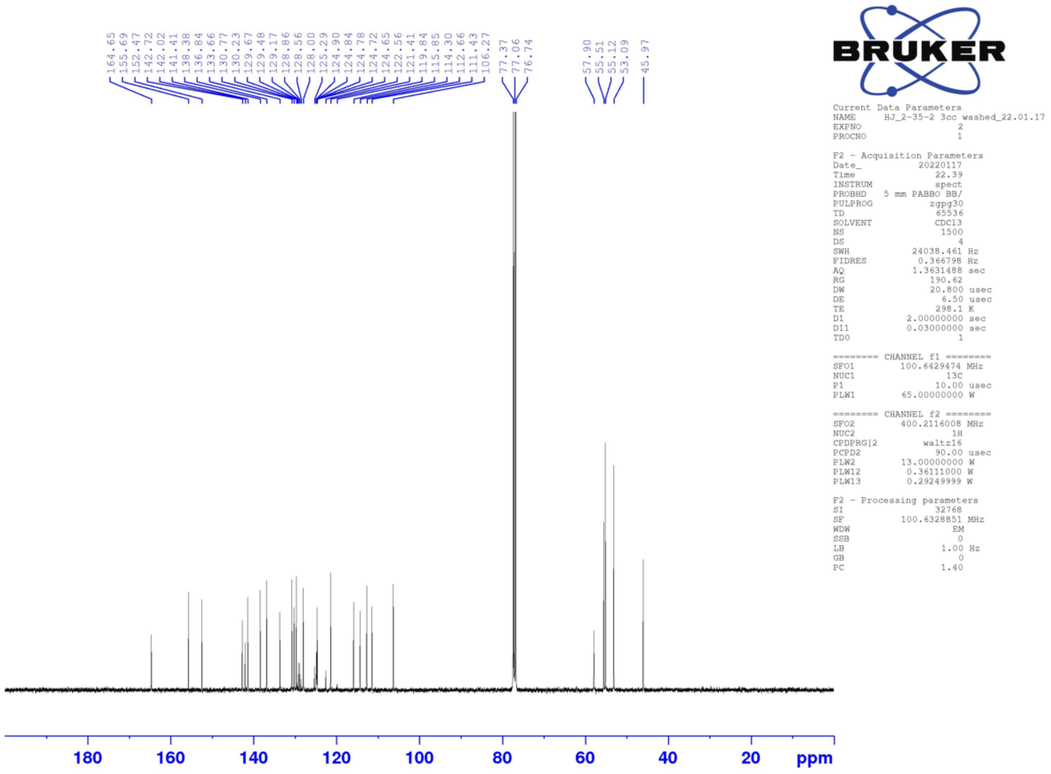
164.65
155.69
152.47
142.72
142.02
141.41
138.38
136.84
133.66
130.77
130.23
129.67
129.48
129.17
128.86
128.56
128.00
125.29
124.90
124.84
124.78
124.72
124.65
122.56
121.41
119.84
115.85
114.30
112.66
111.43
106.27
77.37
77.06
76.74
57.90
55.51
55.12
53.09
45.97
BRUKER
Current Data Parameters
NAME HJ_2-35-2 3cc washed_22.01.17
EXPNO 2
PROCNO 1
F2 - Acquisition Parameters
Date_ 20220117
Time 22.39
INSTRUM spect
PROBHD 5 mm PABBO BB/
PULPROG zgpg30
TD 65536
SOLVENT CDCl3
NS 1500
DS 4
SWH 24038.461 Hz
FIDRES 0.366798 Hz
AQ 1.3631488 sec
RG 190.62
DW 20.800 usec
DE 6.50 usec
TE 298.1 K
D1 2.00000000 sec
D11 0.03000000 sec
TD0 1
======== CHANNEL f1 ========
SFO1 100.6429474 MHz
NUC1 13C
P1 10.00 usec
PLW1 65.00000000 W
======== CHANNEL f2 ========
SFO2 400.2116008 MHz
NUC2 1H
CPDPRG[2 waltz16
PCPD2 90.00 usec
PLW2 13.00000000 W
PLW12 0.36111000 W
PLW13 0.29249999 W
F2 - Processing parameters
SI 32768
SF 100.6328851 MHz
WDW EM
SSB 0
LB 1.00 Hz
GB 0
PC 1.40
180
160
140
120
100
80
60
40
20
ppm

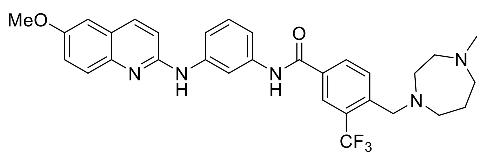

HJ_2-33 = 2-47 (16b)

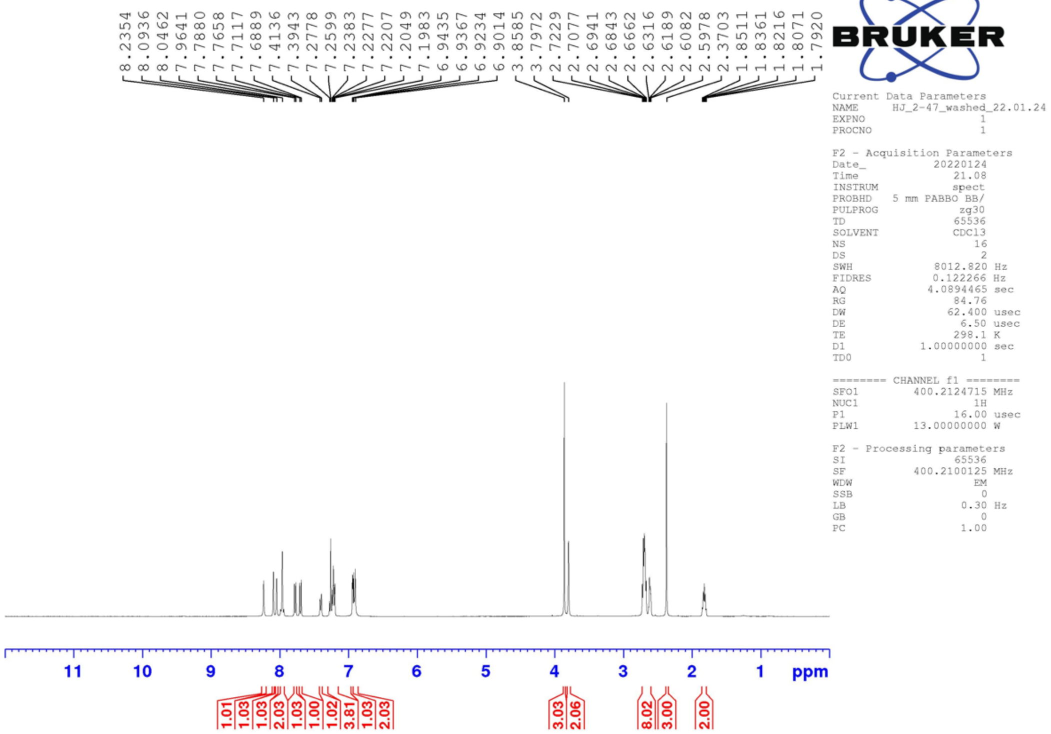

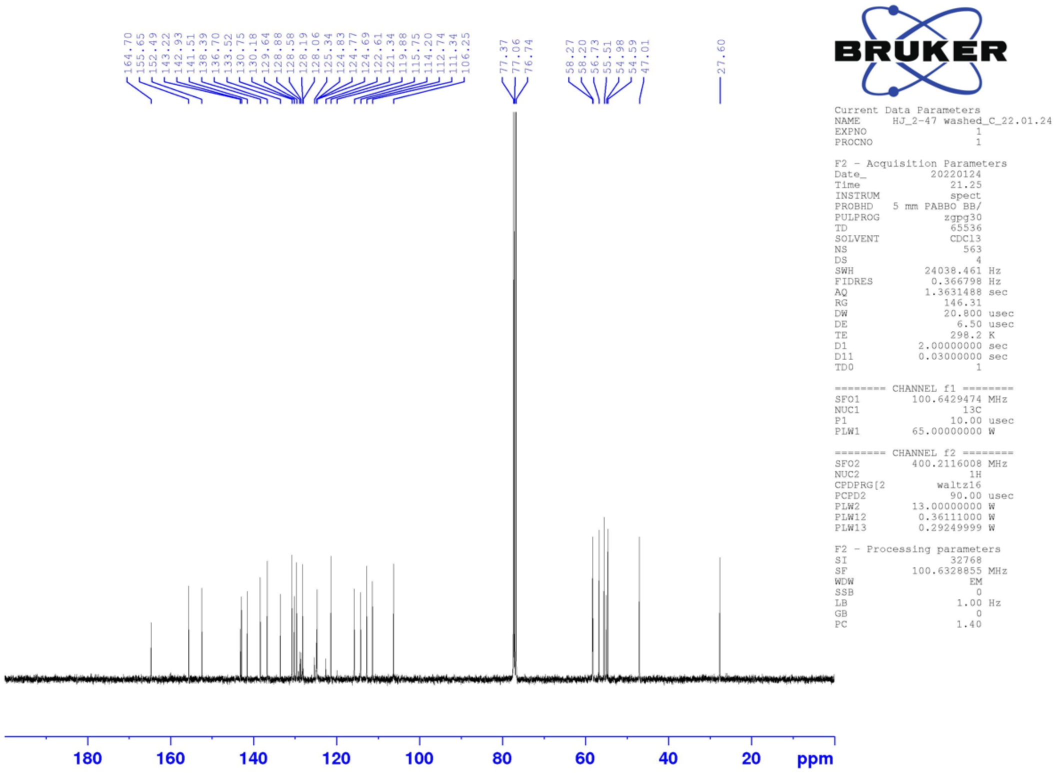

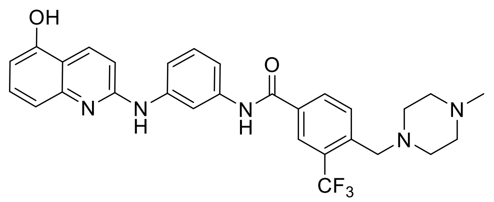
OH
N
N
H
N
H
O
N
N
CF3

HJ_2-14 (17a)

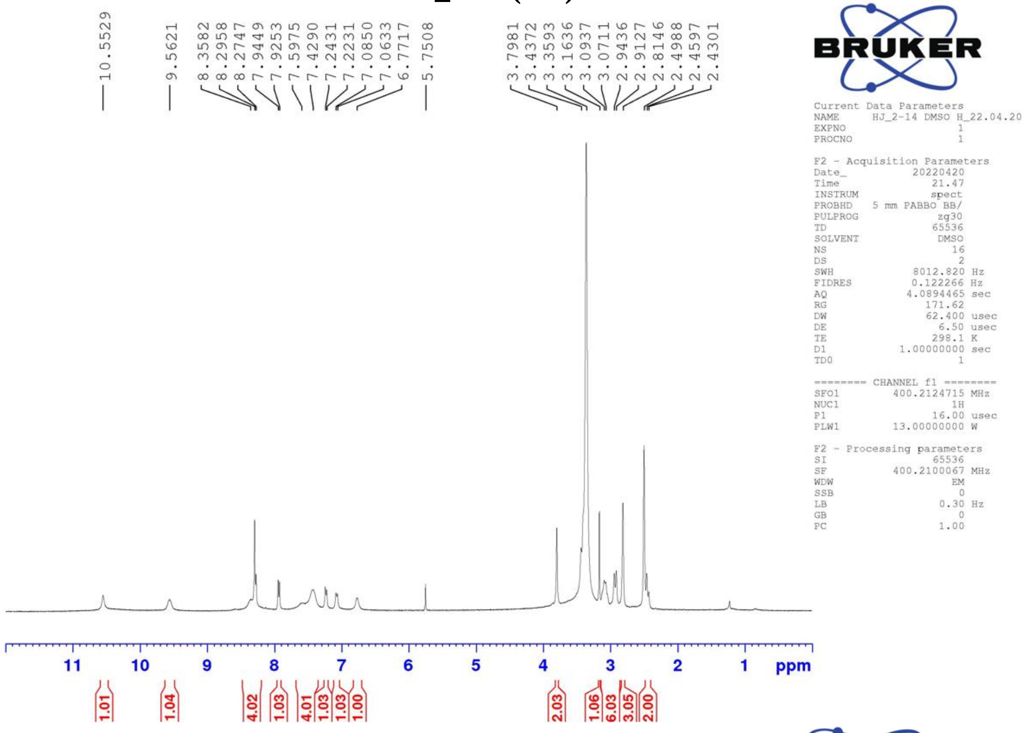
10.5529
9.5621
8.3582
8.2958
8.2747
7.9449
7.9253
7.5975
7.4290
7.2431
7.2231
7.0850
7.0633
6.7717
5.7508
3.7981
3.4372
3.3593
3.1636
3.0937
3.0711
2.9436
2.9127
2.8146
2.4988
2.4597
2.4301
BRUKER
Current Data Parameters
NAME HJ_2-14 DMSO H_22.04.20
EXPNO 1
PROCNO 1
F2 - Acquisition Parameters
Date_ 20220420
Time 21.47
INSTRUM spect
PROBHD 5 mm PABBO BB/
PULPROG zg30
TD 65536
SOLVENT DMSO
NS 16
DS 2
SWH 8012.820 Hz
FIDRES 0.122266 Hz
AQ 4.0894465 sec
RG 171.62
DW 62.400 usec
DE 6.50 usec
TE 298.1 K
D1 1.00000000 sec
TD0 1
======== CHANNEL f1 ========
SFO1 400.2124715 MHz
NUC1 1H
P1 16.00 usec
PLW1 13.00000000 W
F2 - Processing parameters
SI 65536
SF 400.2100067 MHz
WDW EM
SSB 0
LB 0.30 Hz
GB 0
PC 1.00
11
10
9
8
7
6
5
4
3
2
1
ppm
1.01
1.04
4.02
1.03
4.01
1.03
1.03
1.00
2.03
1.06
6.03
3.05
2.00

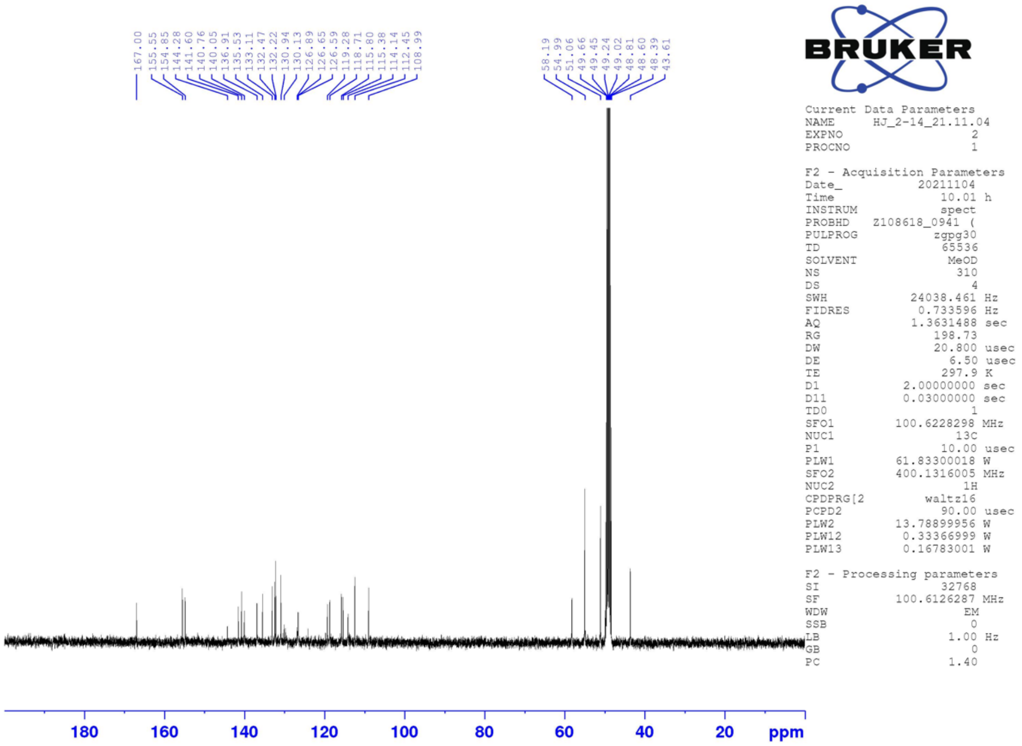
167.00
155.55
154.85
144.28
141.60
140.76
140.05
136.91
135.53
133.11
132.47
132.22
130.94
130.13
126.89
126.65
126.59
119.28
118.71
115.80
115.38
114.14
112.45
108.99
58.19
54.99
51.06
49.66
49.45
49.24
49.02
48.81
48.60
48.39
43.61
BRUKER
Current Data Parameters
NAME HJ_2-14_21.11.04
EXPNO 2
PROCNO 1
F2 - Acquisition Parameters
Date_ 20211104
Time 10.01 h
INSTRUM spect
PROBHD Z108618_0941 (
PULPROG zgpg30
TD 65536
SOLVENT MeOD
NS 310
DS 4
SWH 24038.461 Hz
FIDRES 0.733596 Hz
AQ 1.3631488 sec
RG 198.73
DW 20.800 usec
DE 6.50 usec
TE 297.9 K
D1 2.00000000 sec
D11 0.03000000 sec
TD0 1
SFO1 100.6228298 MHz
NUC1 13C
P1 10.00 usec
PLW1 61.83300018 W
SFO2 400.1316005 MHz
NUC2 1H
CPDPRG[2 waltz16
PCPD2 90.00 usec
PLW2 13.78899956 W
PLW12 0.33366999 W
PLW13 0.16783001 W
F2 - Processing parameters
SI 32768
SF 100.6126287 MHz
WDW EM
SSB 0
LB 1.00 Hz
GB 0
PC 1.40
180
160
140
120
100
80
60
40
20
ppm

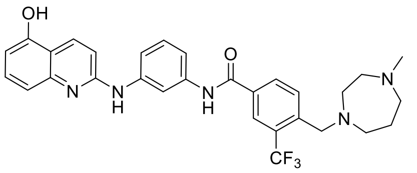
OH
N
N
H
N
H
O
N
N
CF3

HJ_2-12 (17b)

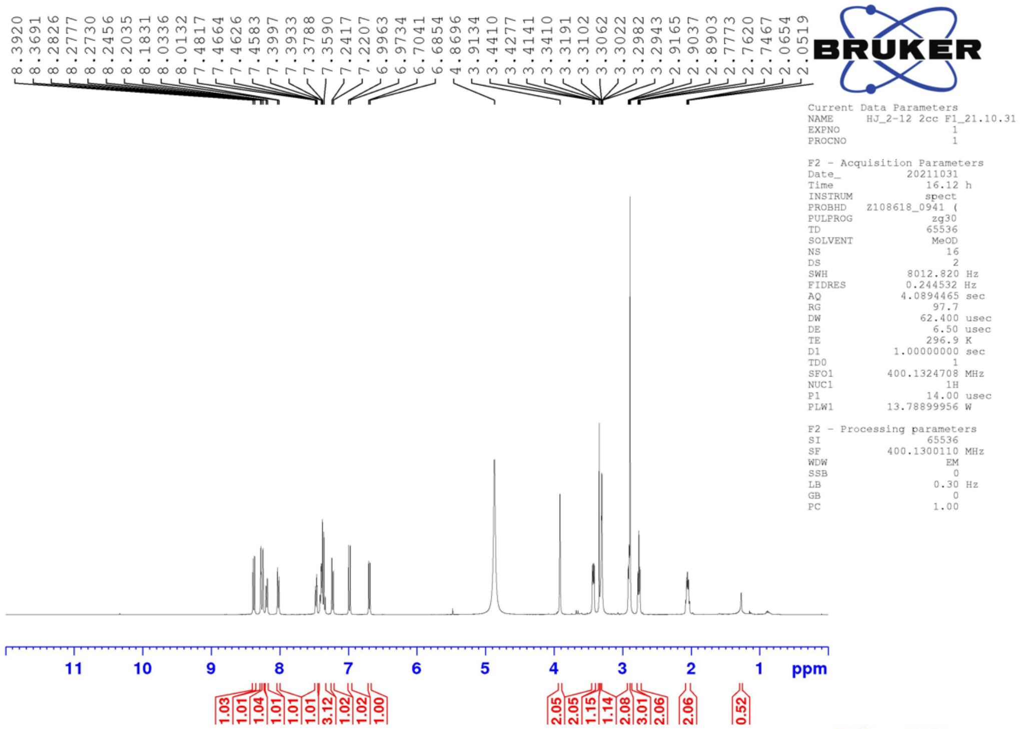
8.3920
8.3691
8.2826
8.2777
8.2730
8.2456
8.2035
8.1831
8.0336
8.0132
7.4817
7.4664
7.4626
7.4583
7.3997
7.3933
7.3788
7.3590
7.2417
7.2207
6.9963
6.9734
6.7041
6.6854
4.8696
3.9134
3.4410
3.4277
3.4141
3.3410
3.3191
3.3102
3.3062
3.3022
3.2982
3.2943
2.9165
2.9037
2.8903
2.7773
2.7620
2.7467
2.0654
2.0519
BRUKER
Current Data Parameters
NAME HJ_2-12 2cc F1_21.10.31
EXPNO 1
PROCNO 1
F2 - Acquisition Parameters
Date_ 20211031
Time 16.12 h
INSTRUM spect
PROBHD Z108618_0941 (
PULPROG zg30
TD 65536
SOLVENT MeOD
NS 16
DS 2
SWH 8012.820 Hz
FIDRES 0.244532 Hz
AQ 4.0894465 sec
RG 97.7
DW 62.400 usec
DE 6.50 usec
TE 296.9 K
D1 1.00000000 sec
TD0 1
SFO1 400.1324708 MHz
NUC1 1H
P1 14.00 usec
PLW1 13.78899956 W
F2 - Processing parameters
SI 65536
SF 400.1300110 MHz
WDW EM
SSB 0
LB 0.30 Hz
GB 0
PC 1.00
11
10
9
8
7
6
5
4
3
2
1
ppm
1.03
1.01
1.04
1.01
1.01
1.01
3.12
1.02
1.02
1.00
2.05
2.05
1.15
1.14
2.08
3.01
2.06
2.06
0.52

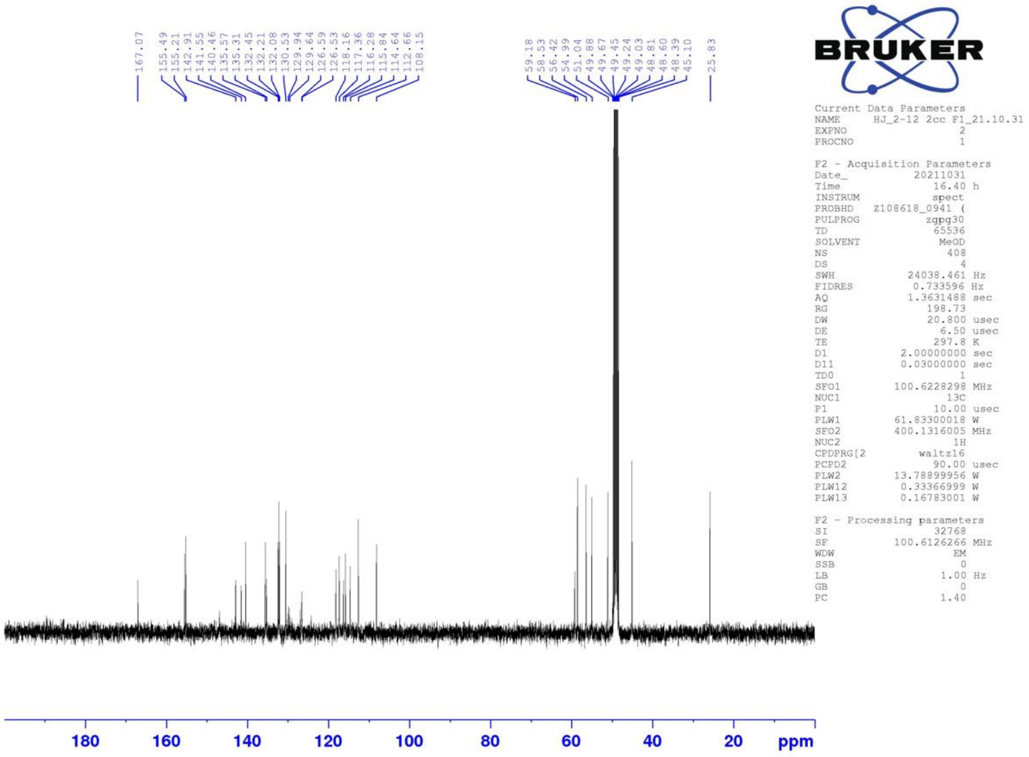
167.07
155.49
155.21
142.91
141.55
140.46
135.57
135.31
132.45
132.21
132.08
130.53
129.94
129.64
126.59
126.53
118.16
117.36
116.28
115.84
114.64
112.66
108.15
59.18
58.53
56.42
54.99
51.04
49.88
49.67
49.45
49.24
49.03
48.81
48.60
48.39
45.10
25.83
BRUKER
Current Data Parameters
NAME HJ_2-12 2cc F1_21.10.31
EXPNO 2
PROCNO 1
F2 - Acquisition Parameters
Date_ 20211031
Time 16.40 h
INSTRUM spect
PROBHD Z108618_0941 (
PULPROG zgpg30
TD 65536
SOLVENT MeOD
NS 408
DS 4
SWH 24038.461 Hz
FIDRES 0.733596 Hz
AQ 1.3631488 sec
RG 198.73
DW 20.800 usec
DE 6.50 usec
TE 297.8 K
D1 2.00000000 sec
D11 0.03000000 sec
TD0 1
SFO1 100.6228298 MHz
NUC1 13C
P1 10.00 usec
PLW1 61.83300018 W
SFO2 400.1316005 MHz
NUC2 1H
CPDPRG[2 waltz16
PCPD2 90.00 usec
PLW2 13.78899956 W
PLW12 0.33366999 W
PLW13 0.16783001 W
F2 - Processing parameters
SI 32768
SF 100.6126266 MHz
WDW EM
SSB 0
LB 1.00 Hz
GB 0
PC 1.40
180
160
140
120
100
80
60
40
20
ppm

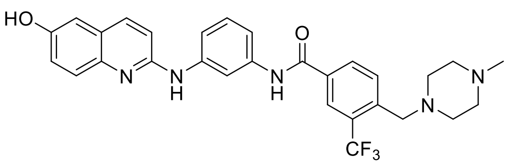
HO
N
H
N
H
N
O
N
N
CF_3

HJ_2-56 (18a)

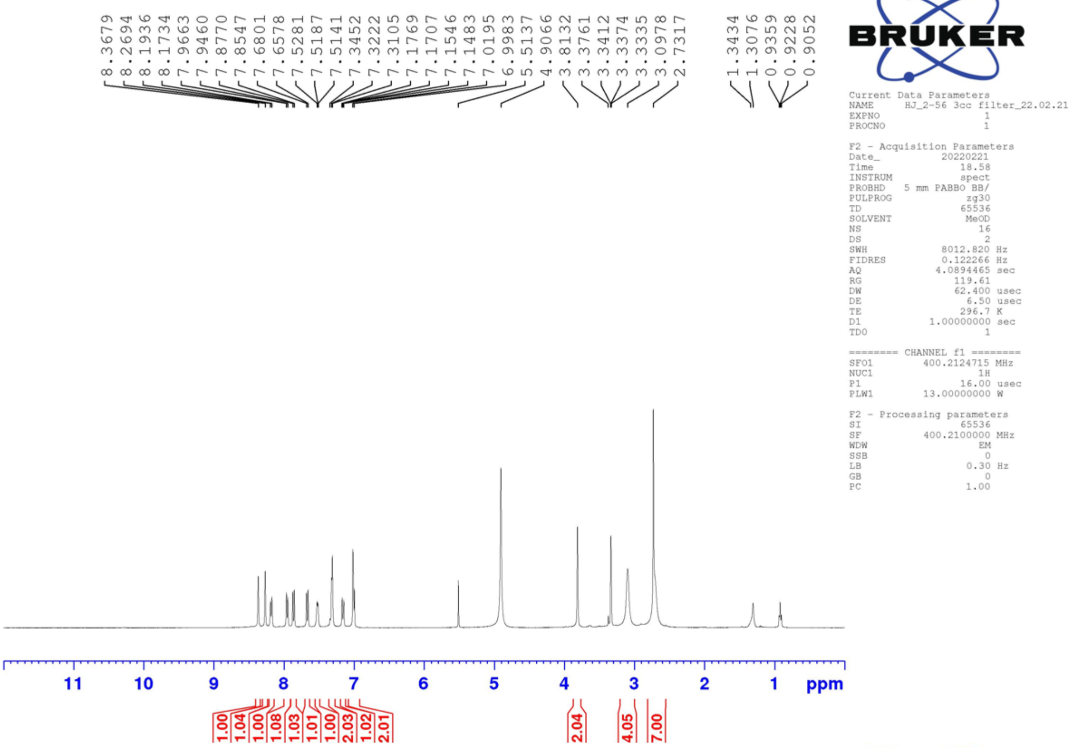
8.3679
8.2694
8.1936
8.1734
7.9663
7.9460
7.8770
7.8547
7.6801
7.6578
7.5281
7.5187
7.5141
7.3452
7.3222
7.3105
7.1769
7.1707
7.1546
7.1483
7.0195
6.9983
5.5137
4.9066
3.8132
3.3761
3.3412
3.3374
3.3335
3.0978
2.7317
1.3434
1.3076
0.9359
0.9228
0.9052
BRUKER
Current Data Parameters
NAME HJ_2-56 3cc filter_22.02.21
EXPNO 1
PROCNO 1
F2 - Acquisition Parameters
Date_ 20220221
Time 18.58
INSTRUM spect
PROBHD 5 mm PABBO BB/
PULPROG zg30
TD 65536
SOLVENT MeOD
NS 16
DS 2
SWH 8012.820 Hz
FIDRES 0.122266 Hz
AQ 4.0894465 sec
RG 119.61
DW 62.400 usec
DE 6.50 usec
TE 296.7 K
D1 1.00000000 sec
TD0 1
======== CHANNEL f1 ========
SFO1 400.2124715 MHz
NUC1 1H
P1 16.00 usec
PLW1 13.00000000 W
F2 - Processing parameters
SI 65536
SF 400.2100000 MHz
WDW EM
SSB 0
LB 0.30 Hz
GB 0
PC 1.00
11
10
9
8
7
6
5
4
3
2
1
ppm
1.00
1.04
1.00
1.08
1.03
1.01
1.00
2.03
1.02
2.01
2.04
4.05
7.00

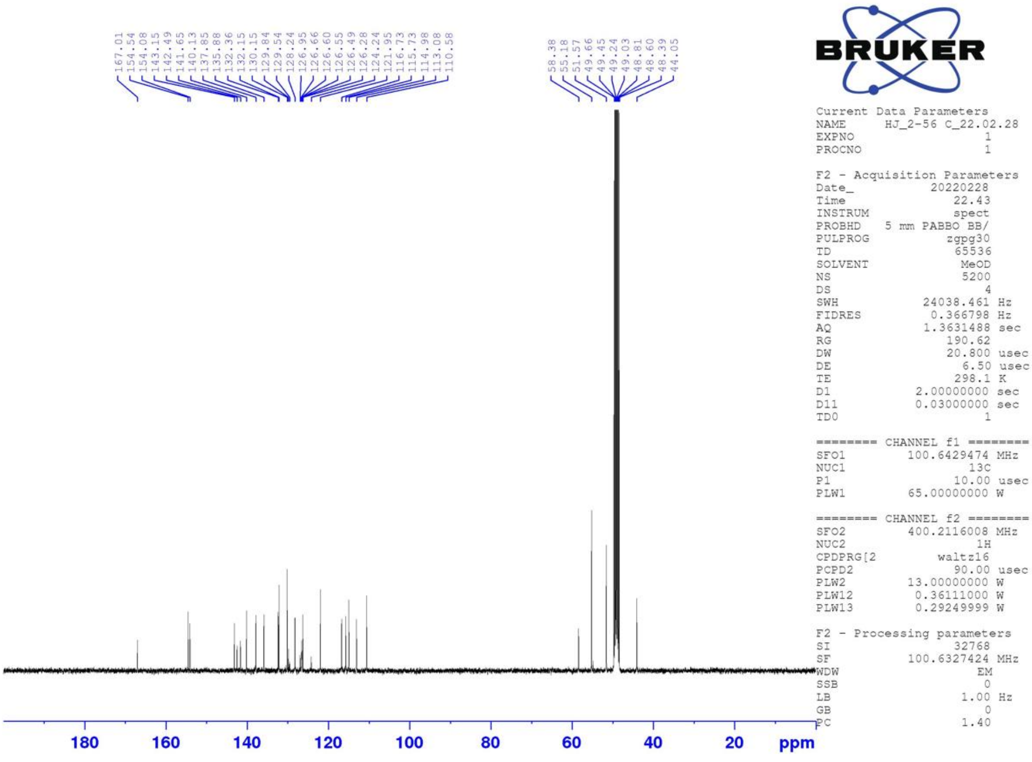
167.01
154.54
154.08
143.15
142.49
141.65
140.13
137.85
135.88
132.36
132.15
130.15
129.84
129.54
128.24
126.95
126.60
126.55
126.49
126.28
124.24
121.95
116.73
115.73
114.98
113.08
110.58
58.38
55.18
51.57
49.66
49.45
49.24
49.03
48.81
48.60
48.39
44.05
BRUKER
Current Data Parameters
NAME HJ_2-56 C_22.02.28
EXPNO 1
PROCNO 1
F2 - Acquisition Parameters
Date_ 20220228
Time 22.43
INSTRUM spect
PROBHD 5 mm PABBO BB/
PULPROG zgpg30
TD 65536
SOLVENT MeOD
NS 5200
DS 4
SWH 24038.461 Hz
FIDRES 0.366798 Hz
AQ 1.3631488 sec
RG 190.62
DW 20.800 usec
DE 6.50 usec
TE 298.1 K
D1 2.00000000 sec
D11 0.03000000 sec
TD0 1
======== CHANNEL f1 ========
SFO1 100.6429474 MHz
NUC1 13C
P1 10.00 usec
PLW1 65.00000000 W
======== CHANNEL f2 ========
SFO2 400.2116008 MHz
NUC2 1H
CPDPRG[2 waltz16
PCPD2 90.00 usec
PLW2 13.00000000 W
PLW12 0.36111000 W
PLW13 0.29249999 W
F2 - Processing parameters
SI 32768
SF 100.6327424 MHz
WDW EM
SSB 0
LB 1.00 Hz
GB 0
PC 1.40
180
160
140
120
100
80
60
40
20
ppm

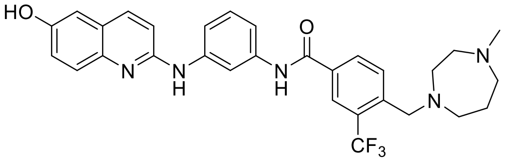
HO
N
N
H
N
H
O
N
N
CF3

HJ_2-57 (18b)

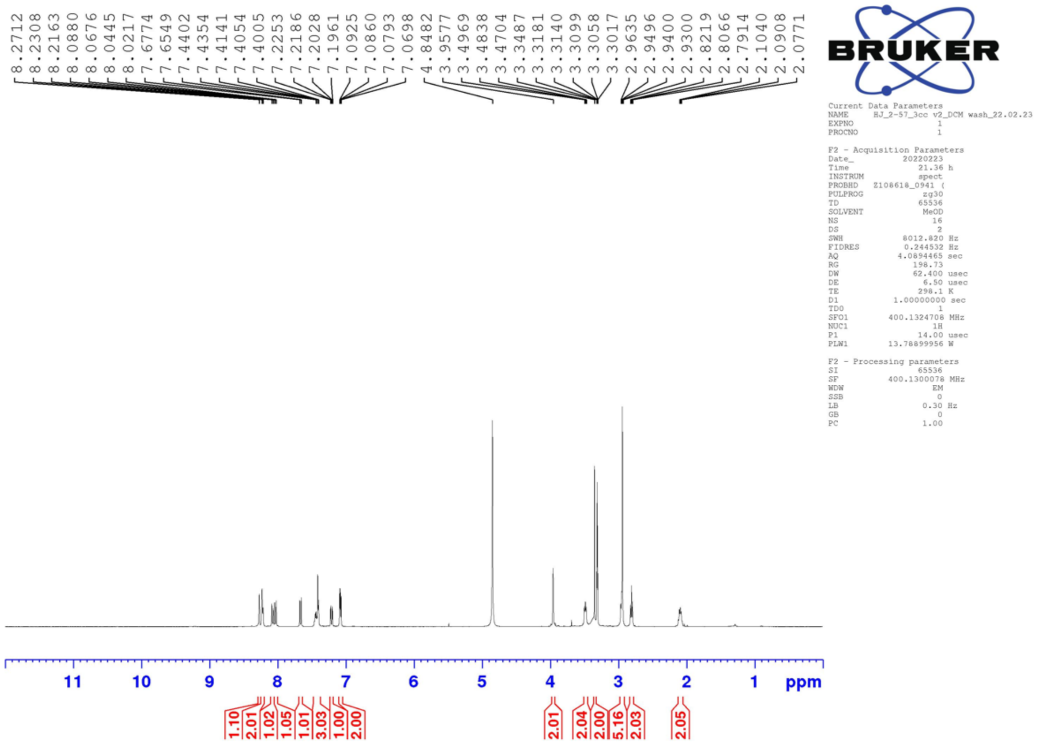
8.2712 8.2308 8.2163 8.0880 8.0676 8.0445 8.0217 7.6774 7.6549 7.4402 7.4354 7.4141 7.4054 7.4005 7.2253 7.2186 7.2028 7.1961 7.0925 7.0860 7.0793 7.0698 4.8482 3.9577 3.4969 3.4838 3.4704 3.3487 3.3181 3.3140 3.3099 3.3058 3.3017 2.9635 2.9496 2.9400 2.9300 2.8219 2.8066 2.7914 2.1040 2.0908 2.0771
BRUKER
Current Data Parameters
NAME HJ_2-57_3cc v2_DCM wash_22.02.23
EXPNO 1
PROCNO 1
F2 - Acquisition Parameters
Date_ 20220223
Time 21.36 h
INSTRUM spect
PROBHD Z108618_0941 (
PULPROG zg30
TD 65536
SOLVENT MeOD
NS 16
DS 2
SWH 8012.820 Hz
FIDRES 0.244532 Hz
AQ 4.0894465 sec
RG 198.73
DW 62.400 usec
DE 6.50 usec
TE 298.1 K
D1 1.00000000 sec
TD0 1
SFO1 400.1324708 MHz
NUC1 1H
P1 14.00 usec
PLW1 13.78899956 W
F2 - Processing parameters
SI 65536
SF 400.1300078 MHz
WDW EM
SSB 0
LB 0.30 Hz
GB 0
PC 1.00
11 10 9 8 7 6 5 4 3 2 1 ppm
1.10 2.01 1.02 1.05 1.01 3.03 1.00 2.00 2.01 2.04 2.00 5.16 2.03 2.05

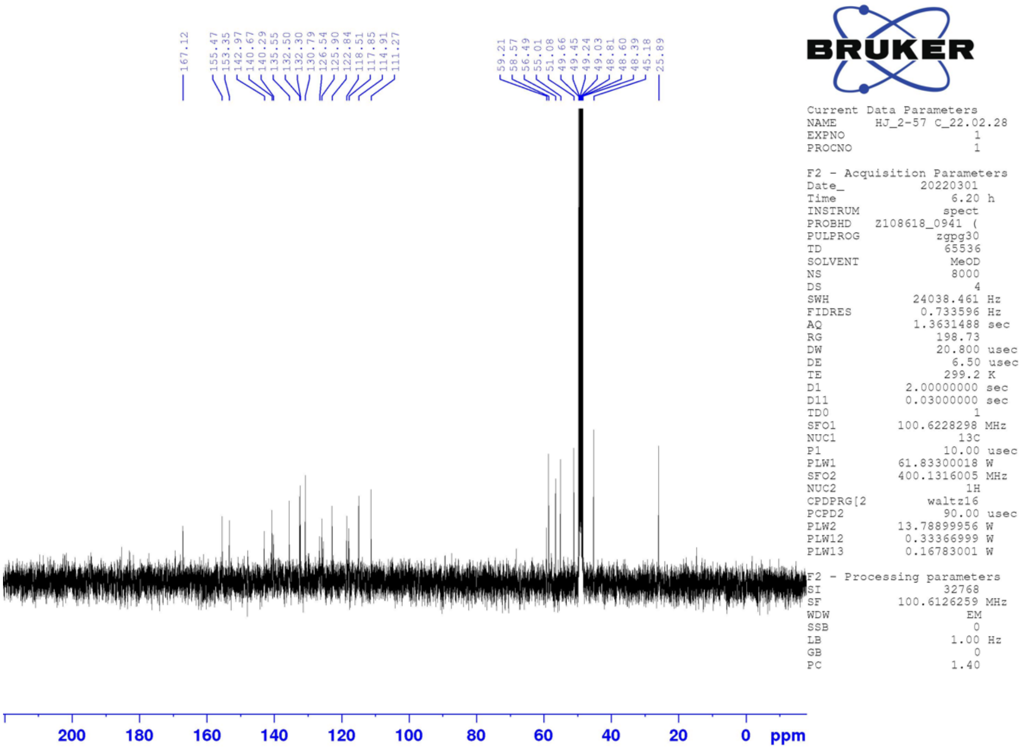
167.12 155.47 153.35 142.97 140.67 140.29 135.55 132.50 132.30 130.79 126.54 125.90 122.84 118.51 117.85 114.91 111.27 59.21 58.57 56.49 55.01 51.08 49.66 49.45 49.24 49.03 48.81 48.60 48.39 45.18 25.89
BRUKER
Current Data Parameters
NAME HJ_2-57 C_22.02.28
EXPNO 1
PROCNO 1
F2 - Acquisition Parameters
Date_ 20220301
Time 6.20 h
INSTRUM spect
PROBHD Z108618_0941 (
PULPROG zgpg30
TD 65536
SOLVENT MeOD
NS 8000
DS 4
SWH 24038.461 Hz
FIDRES 0.733596 Hz
AQ 1.3631488 sec
RG 198.73
DW 20.800 usec
DE 6.50 usec
TE 299.2 K
D1 2.00000000 sec
D11 0.03000000 sec
TD0 1
SFO1 100.6228298 MHz
NUC1 13C
P1 10.00 usec
PLW1 61.83300018 W
SFO2 400.1316005 MHz
NUC2 1H
CPDPRG[2 waltz16
PCPD2 90.00 usec
PLW2 13.78899956 W
PLW12 0.33366999 W
PLW13 0.16783001 W
F2 - Processing parameters
SI 32768
SF 100.6126259 MHz
WDW EM
SSB 0
LB 1.00 Hz
GB 0
PC 1.40
200 180 160 140 120 100 80 60 40 20 0 ppm

2) HRMS charts

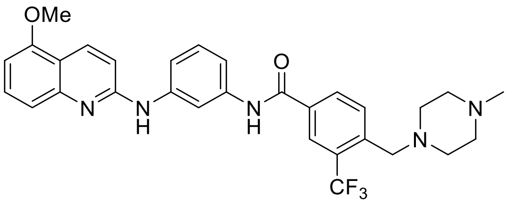

AKA-Vlll-75 (14a)

$C_{30}H_{31}F_3N_5O_2$ $[M+H]^+$:

Calculated: 550.2430

Found: 550.2427

Mass Spectrum: AKE-VIII-75 (Positive mode)

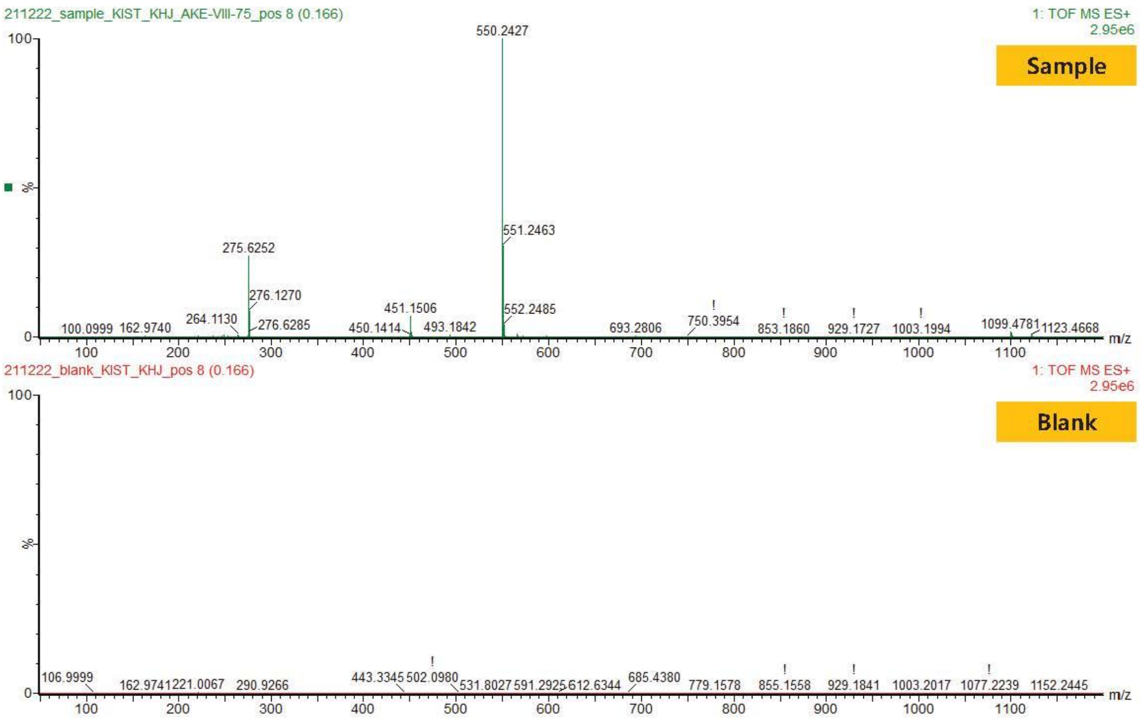

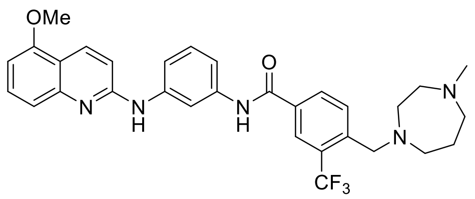

HJ_1-99 = HJ_2-9 (14b)

$C_{31}H_{33}F_3N_5O_2$ $[M+H]^+$:

Calculated: 564.2586

Found: 564.2593

Mass Spectrum: AK-HJ-II-9 (Positive mode)

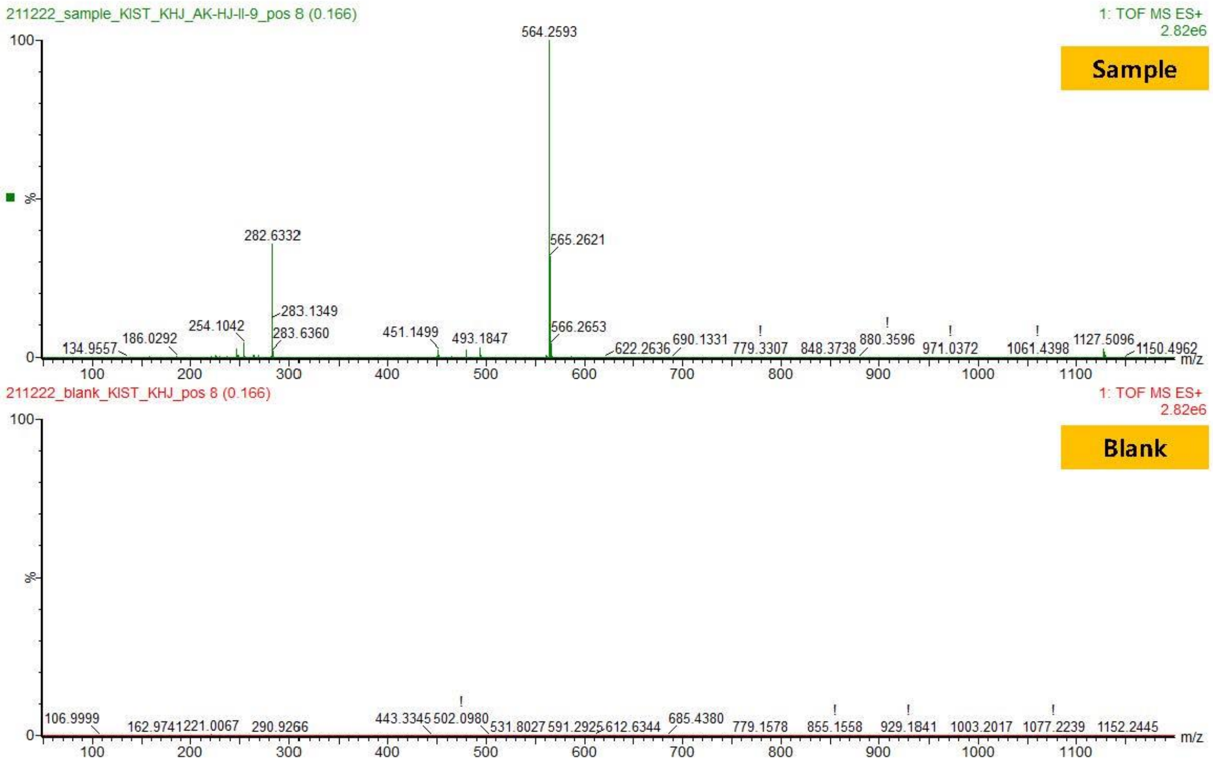

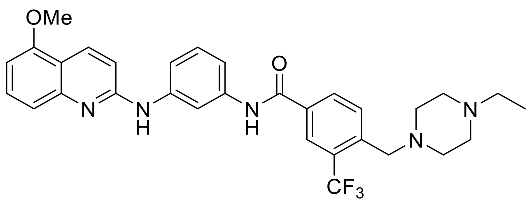

HJ-1-98 (14c)

$C_{31}H_{33}F_3N_5O_2$ $[M+H]^+$:

Calculated: 564.2586

Found: 564.2587

Mass Spectrum: AK-HJ-I-98 (Positive mode)

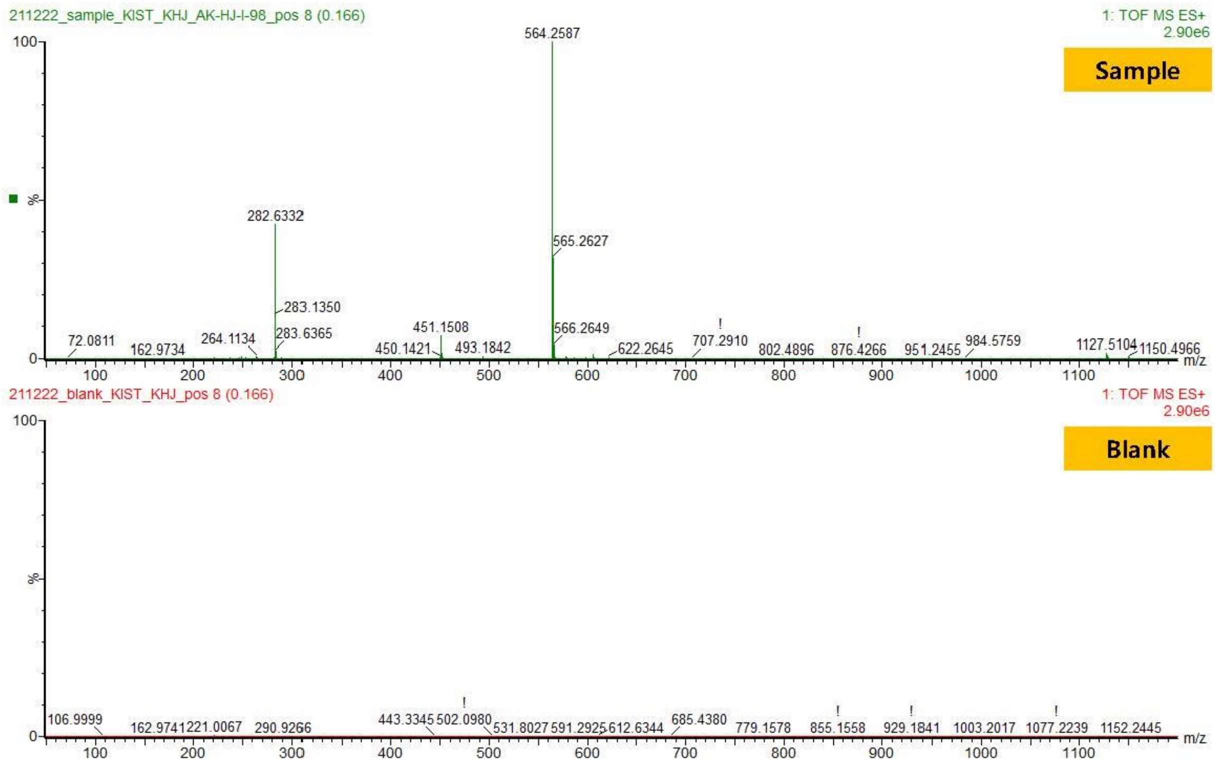

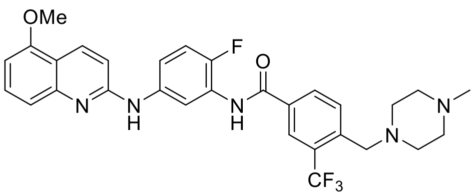

HJ_2-32 (15a)

$C_{30}H_{30}F_4N_5O_2$ $[M+H]^+$:

Calculated: 568.2336

Found: 568.2347

Mass Spectrum: AK-HJ-II-32 (Positive mode)

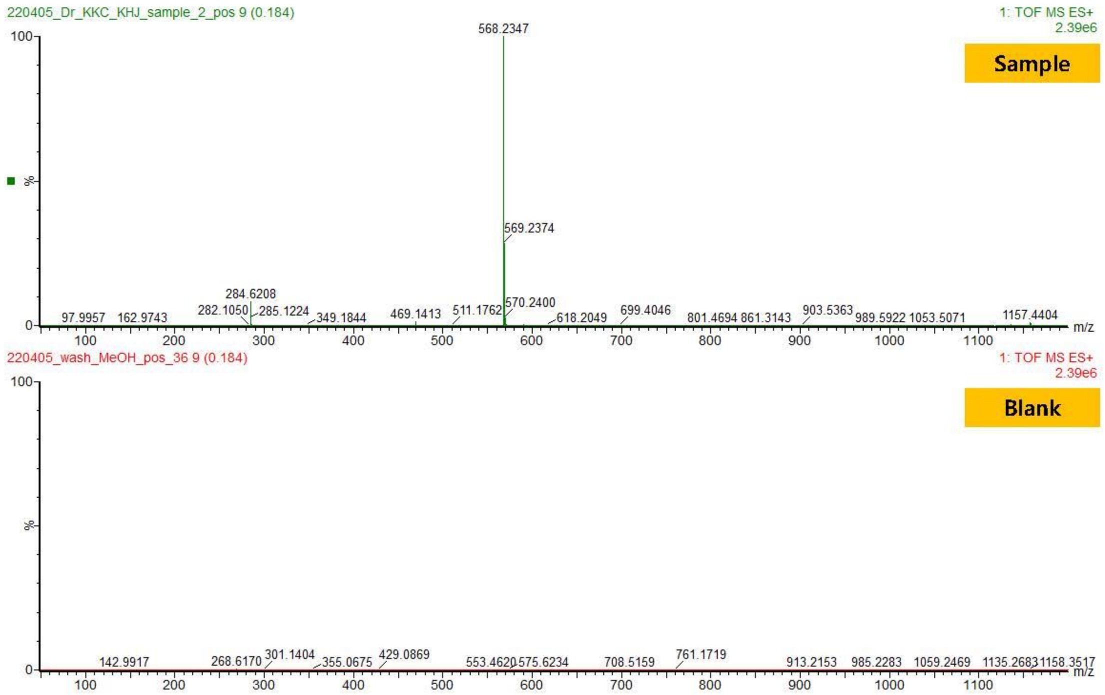

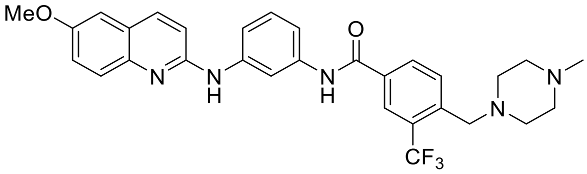

HJ_2-35 (16a)

$C_{30}H_{31}F_3N_5O_2$ $[M+H]^+$:

Calculated: 550.2430

Found: 550.2436

Mass Spectrum: AK-HJ-II-35 (Positive mode)

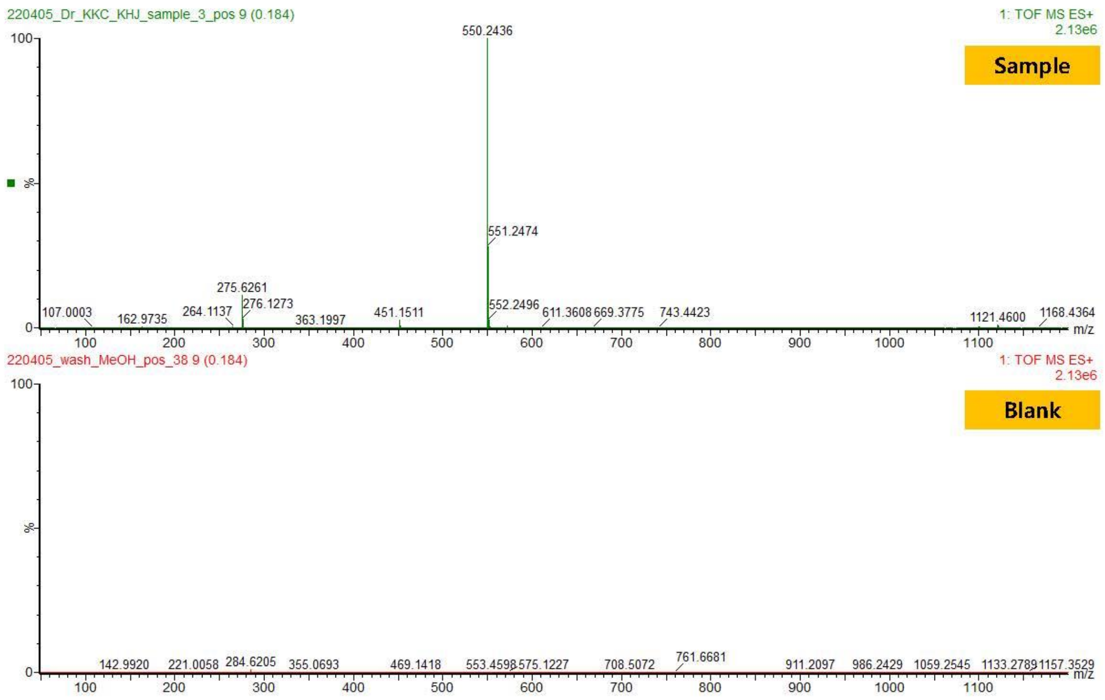

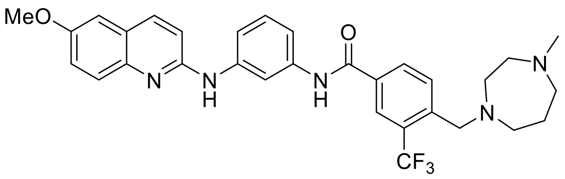

HJ_2-33 = 2-47 (16b)

$C_{31}H_{33}F_3N_5O_2$ $[M+H]^+$:

Calculated: 564.2586

Found: 564.2597

Mass Spectrum: AK-HJ-II-47 (Positive mode)

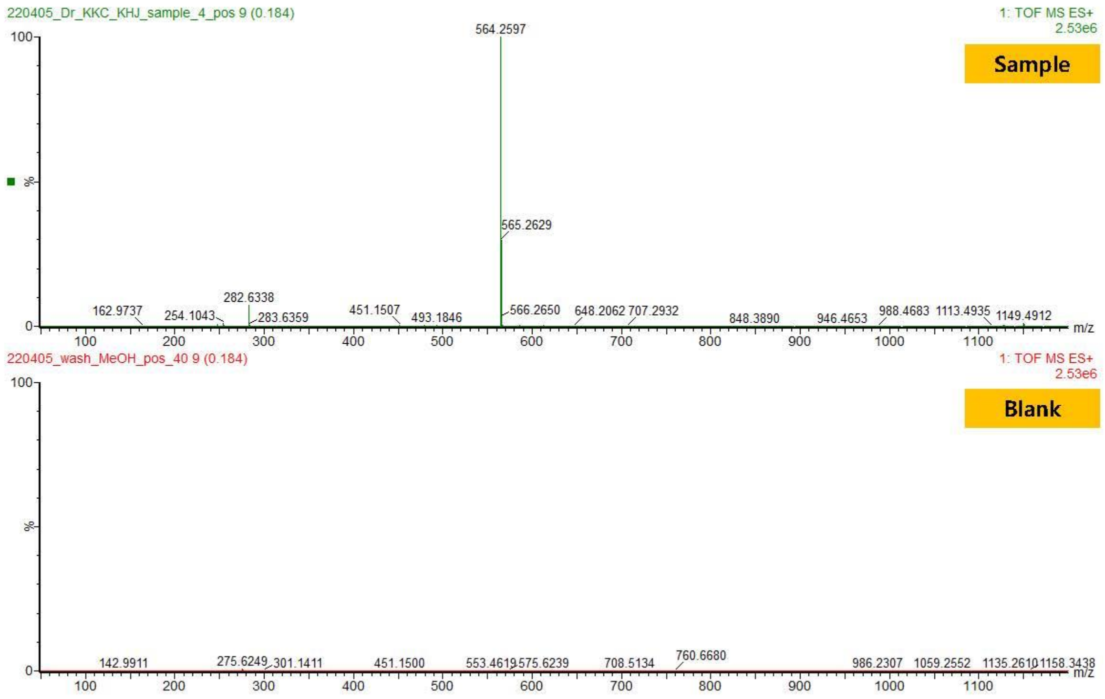

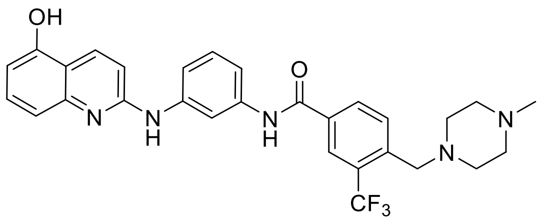

HJ_2-14 (17a)

$C_{29}H_{29}F_3N_5O_2$ $[M+H]^+$:

Calculated: 536.2274

Found: 536.2281

Mass Spectrum: AK-HJ-II-14 (Positive mode)

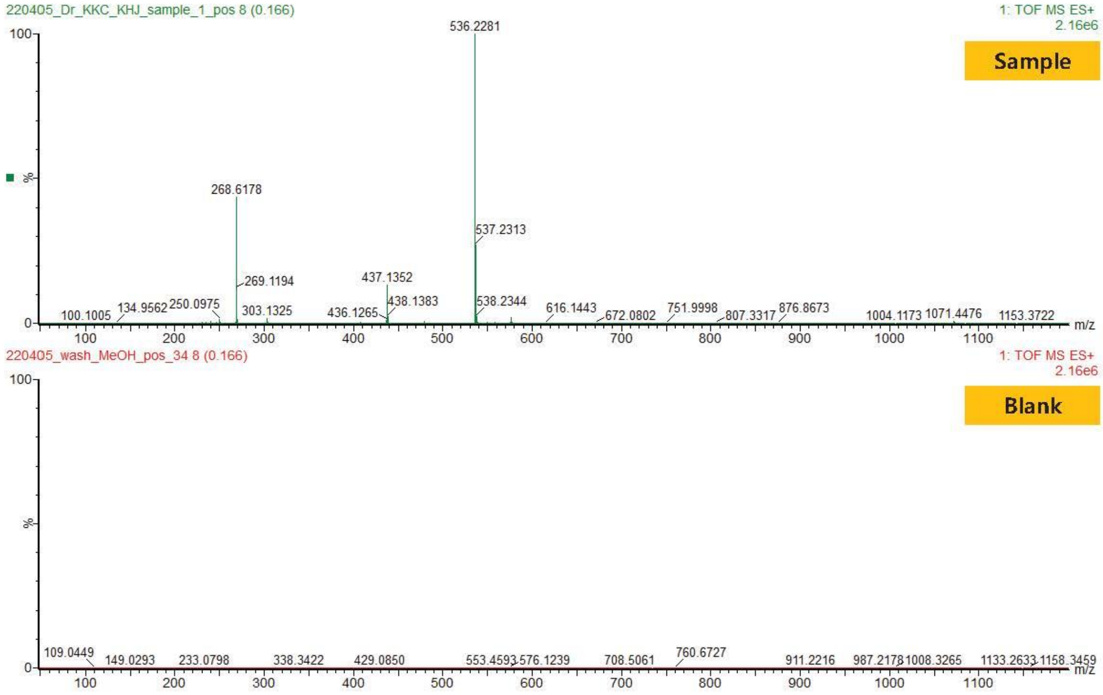

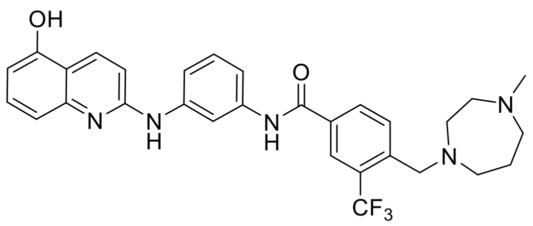

HJ_2-12 (17b)

$C_{30}H_{31}F_3N_5O_2$ $[M+H]^+$:

Calculated: 550.2430

Found: 550.2444

Mass Spectrum: AK-HJ-II-12 (Positive mode)

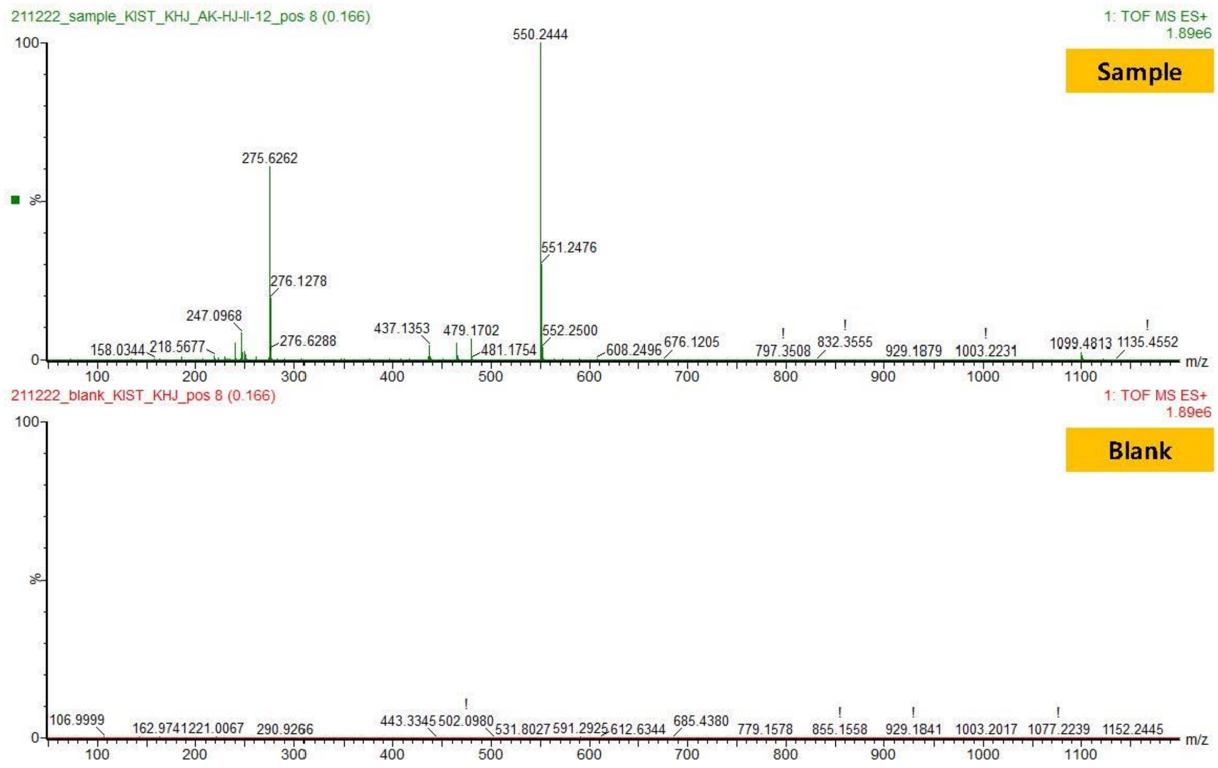

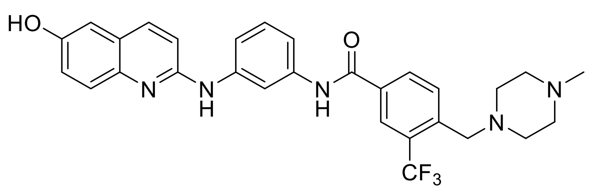

HJ_2-56 (18a)

$C_{29}H_{29}F_3N_5O_2$ $[M+H]^+$:

Calculated: 536.2274

Found: 536.2279

Mass Spectrum: AK-HJ-ll-56 (Positive mode)

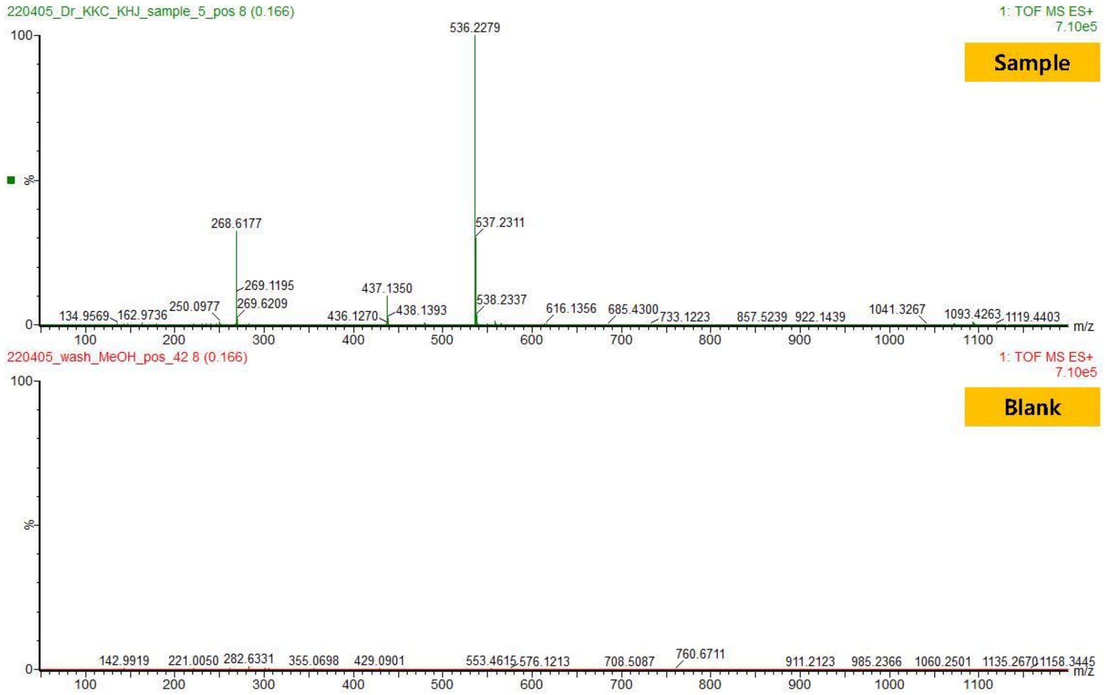

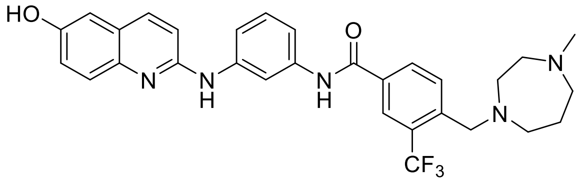

HJ_2-57 (18b)

$C_{30}H_{31}F_3N_5O_2$ $[M+H]^+$:

Calculated: 550.2430

Found: 550.2441

Mass Spectrum: AK-HJ-II-57 (Positive mode)

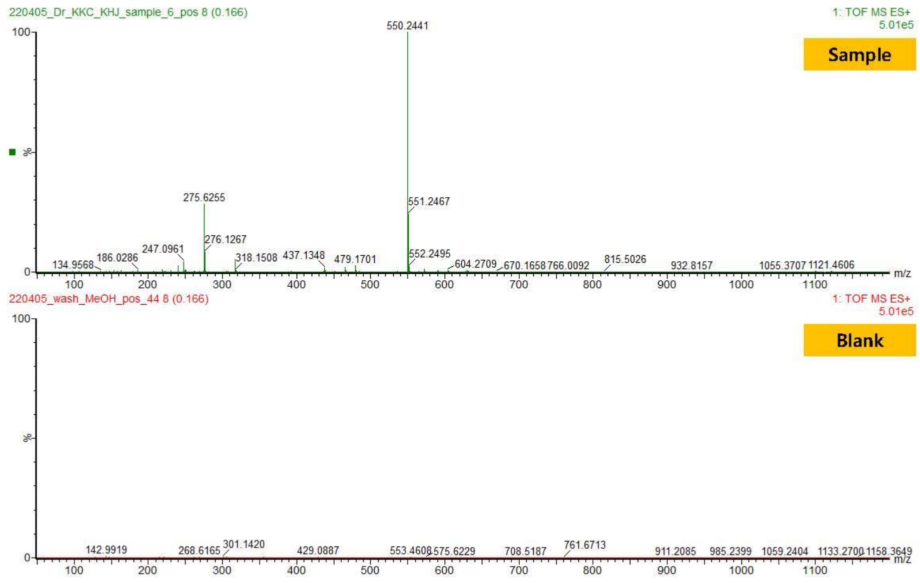

3) Chemical structure of GW5074

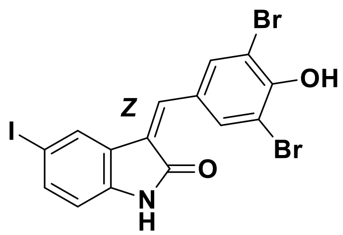

GW5074

4) NCI data

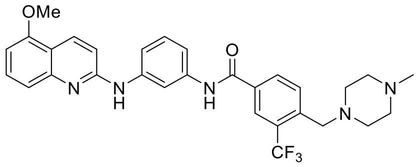

AKA-Vlll-75 (14a)

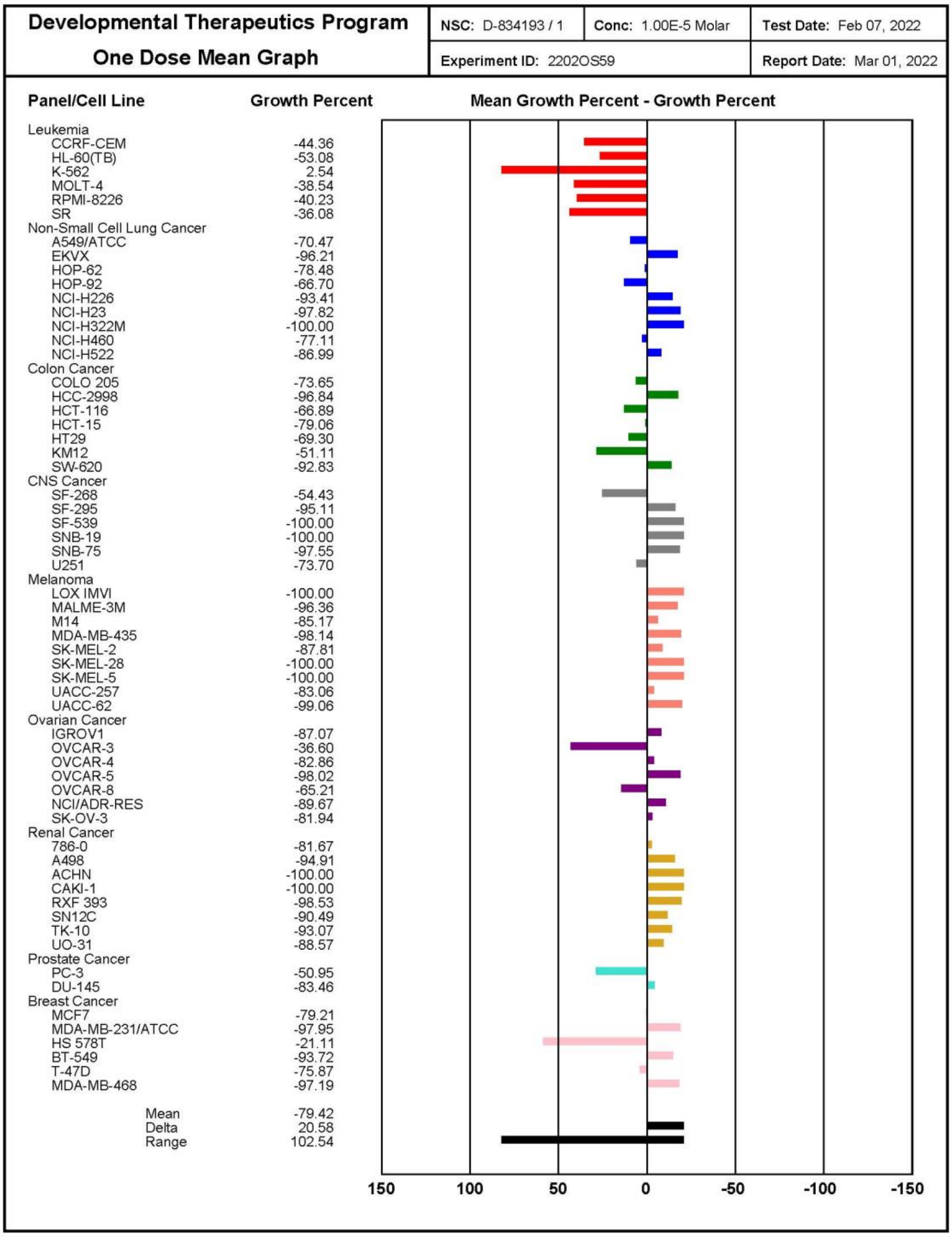

Developmental Therapeutics Program — One Dose Mean Graph

NSC: D-834193 / 1	Conc: 1.00E-5 Molar	Test Date: Feb 07, 2022
Experiment ID: 2202OS59		Report Date: Mar 01, 2022

Panel/Cell Line	Growth Percent
**Leukemia**	
CCRF-CEM	-44.36
HL-60(TB)	-53.08
K-562	2.54
MOLT-4	-38.54
RPMI-8226	-40.23
SR	-36.08
**Non-Small Cell Lung Cancer**	
A549/ATCC	-70.47
EKVX	-96.21
HOP-62	-78.48
HOP-92	-66.70
NCI-H226	-93.41
NCI-H23	-97.82
NCI-H322M	-100.00
NCI-H460	-77.11
NCI-H522	-86.99
**Colon Cancer**	
COLO 205	-73.65
HCC-2998	-96.84
HCT-116	-66.89
HCT-15	-79.06
HT29	-69.30
KM12	-51.11
SW-620	-92.83
**CNS Cancer**	
SF-268	-54.43
SF-295	-95.11
SF-539	-100.00
SNB-19	-100.00
SNB-75	-97.55
U251	-73.70
**Melanoma**	
LOX IMVI	-100.00
MALME-3M	-96.36
M14	-85.17
MDA-MB-435	-98.14
SK-MEL-2	-87.81
SK-MEL-28	-100.00
SK-MEL-5	-100.00
UACC-257	-83.06
UACC-62	-99.06
**Ovarian Cancer**	
IGROV1	-87.07
OVCAR-3	-36.60
OVCAR-4	-82.86
OVCAR-5	-98.02
OVCAR-8	-65.21
NCI/ADR-RES	-89.67
SK-OV-3	-81.94
**Renal Cancer**	
786-0	-81.67
A498	-94.91
ACHN	-100.00
CAKI-1	-100.00
RXF 393	-98.53
SN12C	-90.49
TK-10	-93.07
UO-31	-88.57
**Prostate Cancer**	
PC-3	-50.95
DU-145	-83.46
**Breast Cancer**	
MCF7	-79.21
MDA-MB-231/ATCC	-97.95
HS 578T	-21.11
BT-549	-93.72
T-47D	-75.87
MDA-MB-468	-97.19
Mean	-79.42
Delta	20.58
Range	102.54

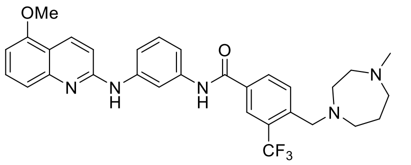

HJ_1-99 = HJ_2-9 (14b)

**Developmental Therapeutics Program**	**NSC:** D-834350 / 1	**Conc:** 1.00E-5 Molar	**Test Date:** Feb 28, 2022
**One Dose Mean Graph**	**Experiment ID:** 2202OS75		**Report Date:** Mar 17, 2022

Panel/Cell Line	Growth Percent
Leukemia	
CCRF-CEM	-54.31
HL-60(TB)	-54.27
K-562	5.93
MOLT-4	-42.81
RPMI-8226	-39.42
SR	-27.14
Non-Small Cell Lung Cancer	
A549/ATCC	-75.60
EKVX	-98.85
HOP-62	-74.72
HOP-92	-83.47
NCI-H226	-70.50
NCI-H23	-97.93
NCI-H322M	-100.00
NCI-H460	-83.59
NCI-H522	-67.46
Colon Cancer	
COLO 205	-56.91
HCC-2998	-96.55
HCT-116	-64.66
HCT-15	-100.00
HT29	-72.03
KM12	-63.86
SW-620	-97.46
CNS Cancer	
SF-268	-50.29
SF-295	-97.71
SF-539	-100.00
SNB-19	-99.28
SNB-75	-84.53
U251	-55.57
Melanoma	
LOX IMVI	-98.81
MALME-3M	-96.32
M14	-90.53
MDA-MB-435	-100.00
SK-MEL-2	-87.39
SK-MEL-28	-100.00
SK-MEL-5	-96.87
UACC-257	-86.01
UACC-62	-74.05
Ovarian Cancer	
IGROV1	-85.23
OVCAR-3	-58.61
OVCAR-4	-84.38
OVCAR-5	-100.00
OVCAR-8	-69.29
NCI/ADR-RES	-77.90
SK-OV-3	-72.83
Renal Cancer	
786-0	-84.92
A498	-91.05
ACHN	-100.00
CAKI-1	-100.00
SN12C	-79.87
TK-10	-89.35
UO-31	-97.23
Prostate Cancer	
PC-3	-69.80
DU-145	-83.37
Breast Cancer	
MCF7	-90.00
MDA-MB-231/ATCC	-99.14
HS 578T	-61.72
BT-549	-92.50
T-47D	-55.89
MDA-MB-468	-87.91
Mean	-79.05
Delta	20.95
Range	105.93

Mean Growth Percent - Growth Percent

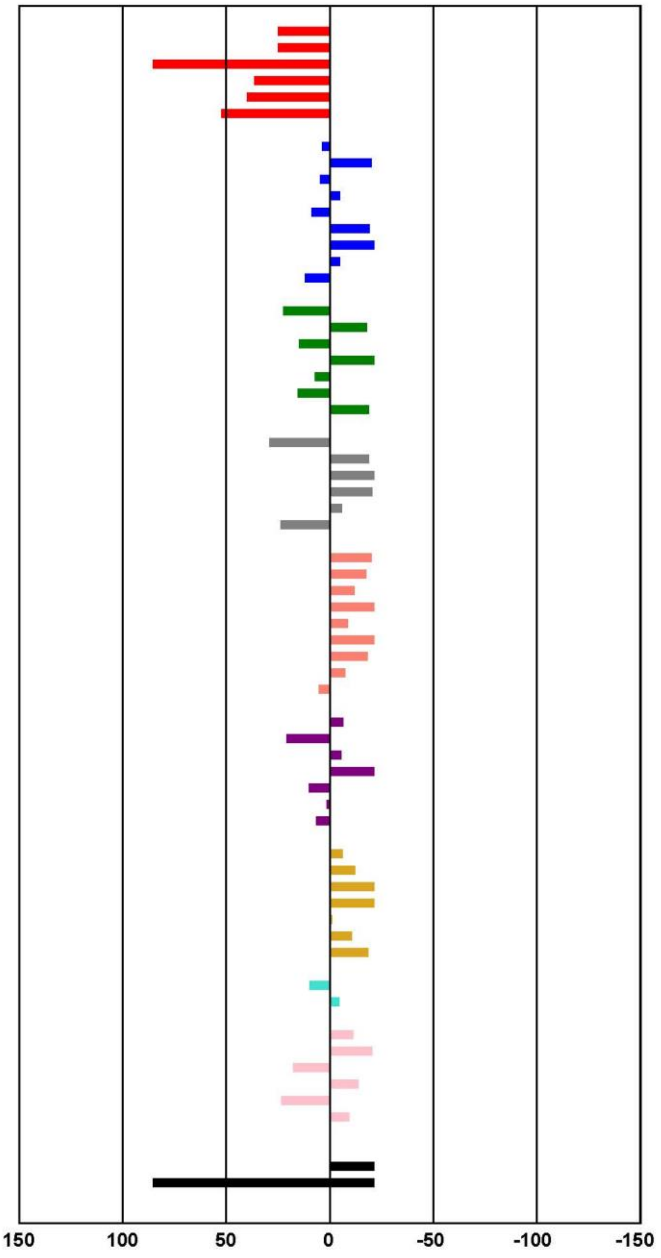

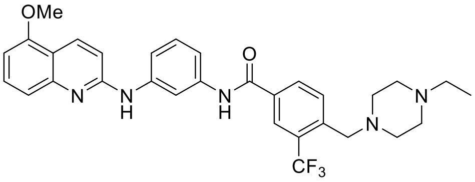

HJ-1-98 (14c)

Developmental Therapeutics Program One Dose Mean Graph	NSC: D-834194 / 1	Conc: 1.00E-5 Molar	Test Date: Feb 07, 2022
	Experiment ID: 2202OS59		Report Date: Mar 01, 2022

Panel/Cell Line	Growth Percent
**Leukemia**	
CCRF-CEM	-30.52
HL-60(TB)	-57.13
K-562	0.42
MOLT-4	-45.43
RPMI-8226	-34.83
SR	-35.07
**Non-Small Cell Lung Cancer**	
A549/ATCC	-68.03
EKVX	-95.72
HOP-62	-63.45
HOP-92	-75.09
NCI-H226	-85.79
NCI-H23	-96.15
NCI-H322M	-100.00
NCI-H460	-68.99
NCI-H522	-83.28
**Colon Cancer**	
COLO 205	-67.46
HCC-2998	-94.01
HCT-116	-56.42
HCT-15	-83.35
HT29	-67.57
KM12	-37.59
SW-620	7.01
**CNS Cancer**	
SF-268	-19.06
SF-295	-95.33
SF-539	-100.00
SNB-19	-99.72
SNB-75	-89.69
U251	-80.85
**Melanoma**	
LOX IMVI	-92.11
MALME-3M	-94.77
M14	-74.80
MDA-MB-435	-94.90
SK-MEL-2	-76.03
SK-MEL-28	-100.00
SK-MEL-5	-99.20
UACC-257	-82.77
UACC-62	-95.13
**Ovarian Cancer**	
IGROV1	-83.61
OVCAR-3	4.54
OVCAR-4	-53.13
OVCAR-5	-91.93
OVCAR-8	-66.11
NCI/ADR-RES	-81.04
SK-OV-3	-8.33
**Renal Cancer**	
786-0	-73.00
A498	-88.78
ACHN	-100.00
CAKI-1	-98.32
RXF 393	-93.27
SN12C	-90.48
TK-10	-86.17
UO-31	-85.81
**Prostate Cancer**	
PC-3	-38.64
DU-145	-57.91
**Breast Cancer**	
MCF7	-89.05
MDA-MB-231/ATCC	-94.01
HS 578T	-16.44
BT-549	-94.00
T-47D	-71.42
MDA-MB-468	-94.66
Mean	-71.41
Delta	28.59
Range	107.01

Mean Growth Percent - Growth Percent

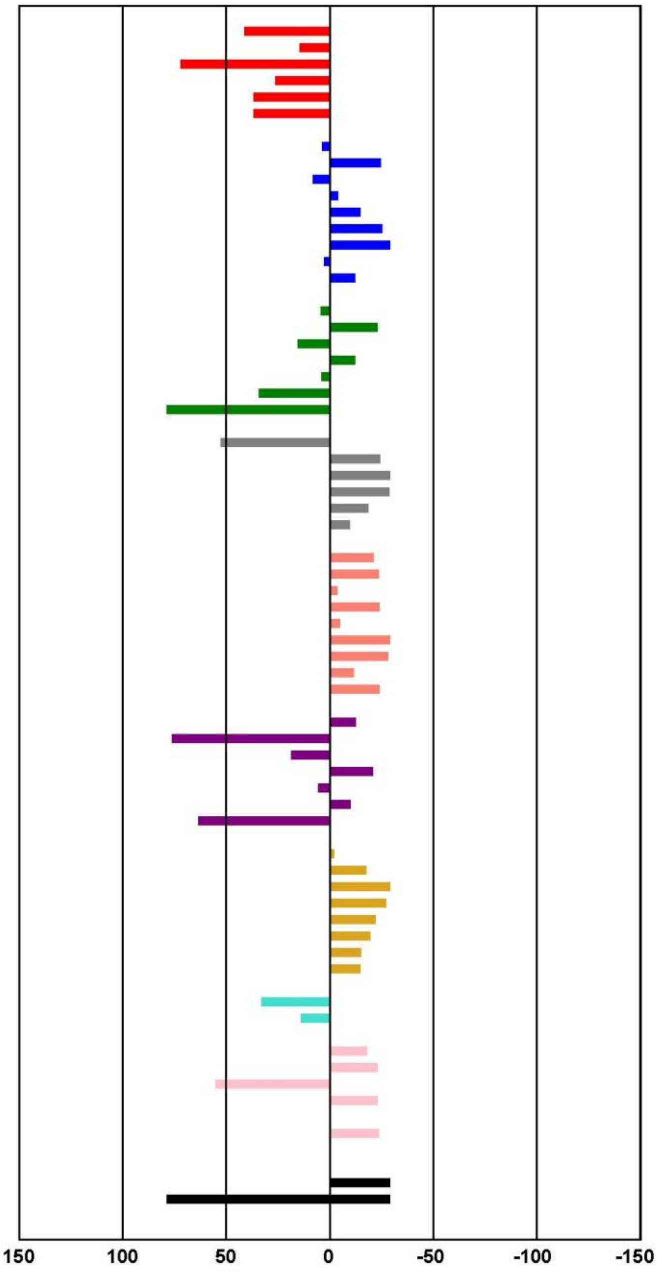

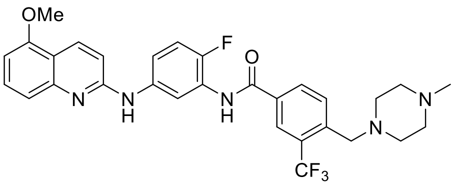

HJ_2-32 (15a)

Developmental Therapeutics Program

One Dose Mean Graph

NSC: D-837518 / 1 | **Conc:** 1.00E-5 Molar | **Test Date:** Apr 25, 2022

Experiment ID: 2204OS09 | **Report Date:** Jun 06, 2022

Panel/Cell Line	Growth Percent
**Leukemia**	
CCRF-CEM	-3.00
HL-60(TB)	-73.12
K-562	-70.81
MOLT-4	-16.49
RPMI-8226	-43.88
**Non-Small Cell Lung Cancer**	
A549/ATCC	18.55
EKVX	13.02
HOP-62	-36.37
HOP-92	-22.85
NCI-H226	16.97
NCI-H23	-17.76
NCI-H322M	32.71
NCI-H460	-68.84
NCI-H522	-60.89
**Colon Cancer**	
COLO 205	-78.70
HCC-2998	-94.49
HCT-116	1.84
HCT-15	-82.97
HT29	-30.25
KM12	-22.22
SW-620	-63.03
**CNS Cancer**	
SF-268	22.18
SF-295	-84.00
SF-539	-93.58
SNB-19	-49.17
SNB-75	-3.82
U251	-42.70
**Melanoma**	
LOX IMVI	-100.00
MALME-3M	-91.33
M14	-12.01
MDA-MB-435	-69.35
SK-MEL-2	14.59
SK-MEL-28	-84.03
SK-MEL-5	-94.93
UACC-257	-66.52
UACC-62	-82.06
**Ovarian Cancer**	
IGROV1	29.31
OVCAR-3	-33.27
OVCAR-4	32.63
OVCAR-5	72.52
OVCAR-8	30.94
NCI/ADR-RES	-3.13
SK-OV-3	53.81
**Renal Cancer**	
786-0	-66.29
A498	77.02
ACHN	0.66
CAKI-1	-2.36
RXF 393	-27.08
SN12C	26.76
TK-10	50.28
UO-31	5.08
**Prostate Cancer**	
PC-3	-8.36
DU-145	6.64
**Breast Cancer**	
MCF7	-61.35
MDA-MB-231/ATCC	-37.81
HS 578T	6.57
BT-549	82.60
T-47D	21.99
MDA-MB-468	-48.88
Mean	-22.56
Delta	77.44
Range	182.60

Mean Growth Percent - Growth Percent

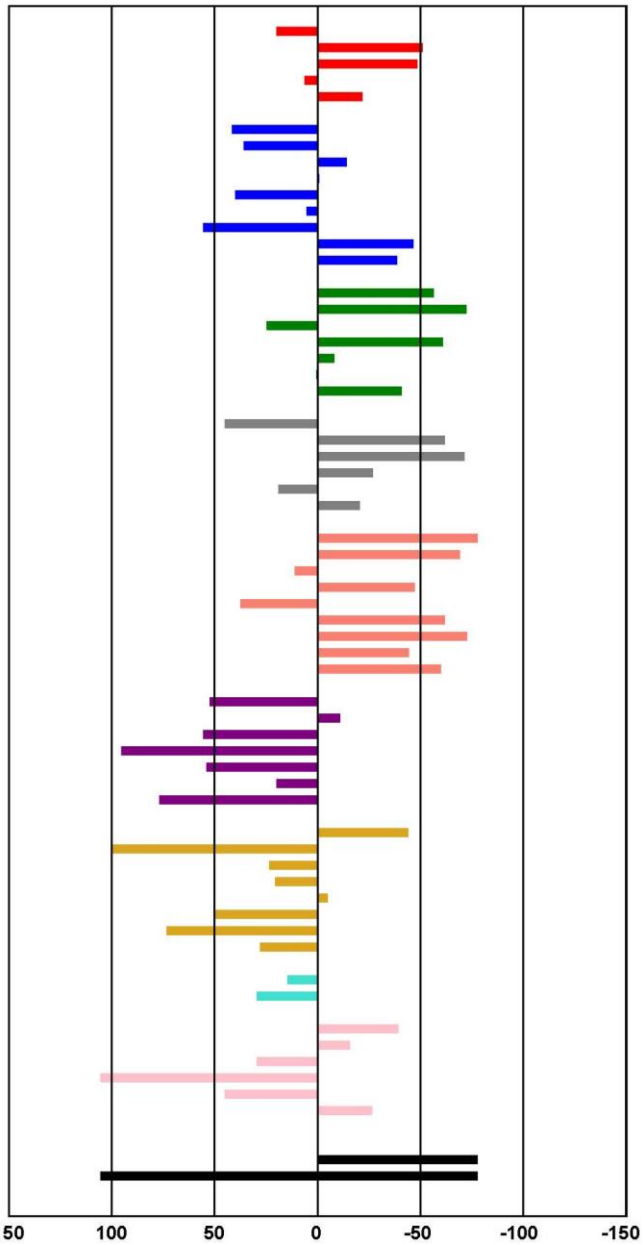

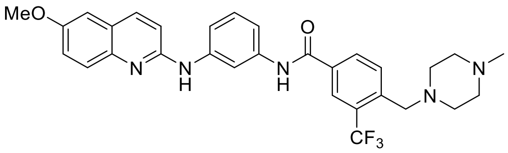

HJ_2-35 (16a)

Developmental Therapeutics Program One Dose Mean Graph	NSC: D-837513 / 1	Conc: 1.00E-5 Molar	Test Date: Apr 25, 2022
	Experiment ID: 2204OS09		Report Date: Oct 24, 2022

Panel/Cell Line	Growth Percent
Leukemia	
CCRF-CEM	-10.53
HL-60(TB)	-47.80
K-562	-70.81
MOLT-4	-44.63
RPMI-8226	-32.98
Non-Small Cell Lung Cancer	
A549/ATCC	-54.73
EKVX	-98.03
HOP-62	-77.60
HOP-92	-57.01
NCI-H226	-46.89
NCI-H23	-94.90
NCI-H322M	-64.01
NCI-H460	-84.53
NCI-H522	-85.72
Colon Cancer	
COLO 205	-90.31
HCC-2998	-99.41
HCT-116	-15.32
HCT-15	-83.42
HT29	-73.93
KM12	-68.10
SW-620	-89.69
CNS Cancer	
SF-268	-55.18
SF-295	-97.75
SF-539	-99.82
SNB-19	-96.76
SNB-75	-39.51
U251	-77.48
Melanoma	
LOX IMVI	-100.00
MALME-3M	-91.81
M14	-50.15
MDA-MB-435	-95.99
SK-MEL-2	-98.28
SK-MEL-28	-96.88
SK-MEL-5	-100.00
UACC-257	-83.88
UACC-62	-92.97
Ovarian Cancer	
IGROV1	-69.83
OVCAR-3	-36.05
OVCAR-4	-27.07
OVCAR-5	-74.96
OVCAR-8	-38.11
NCI/ADR-RES	-37.92
SK-OV-3	-48.78
Renal Cancer	
786-0	-55.29
A498	-42.30
ACHN	-94.83
CAKI-1	-33.74
RXF 393	-75.74
SN12C	-92.60
TK-10	-44.40
UO-31	-48.25
Prostate Cancer	
PC-3	-8.06
DU-145	-32.82
Breast Cancer	
MCF7	-97.14
MDA-MB-231/ATCC	-82.66
HS 578T	-23.08
BT-549	-45.77
T-47D	-25.89
MDA-MB-468	-97.09
Mean	-66.09
Delta	33.91
Range	91.94

Mean Growth Percent - Growth Percent

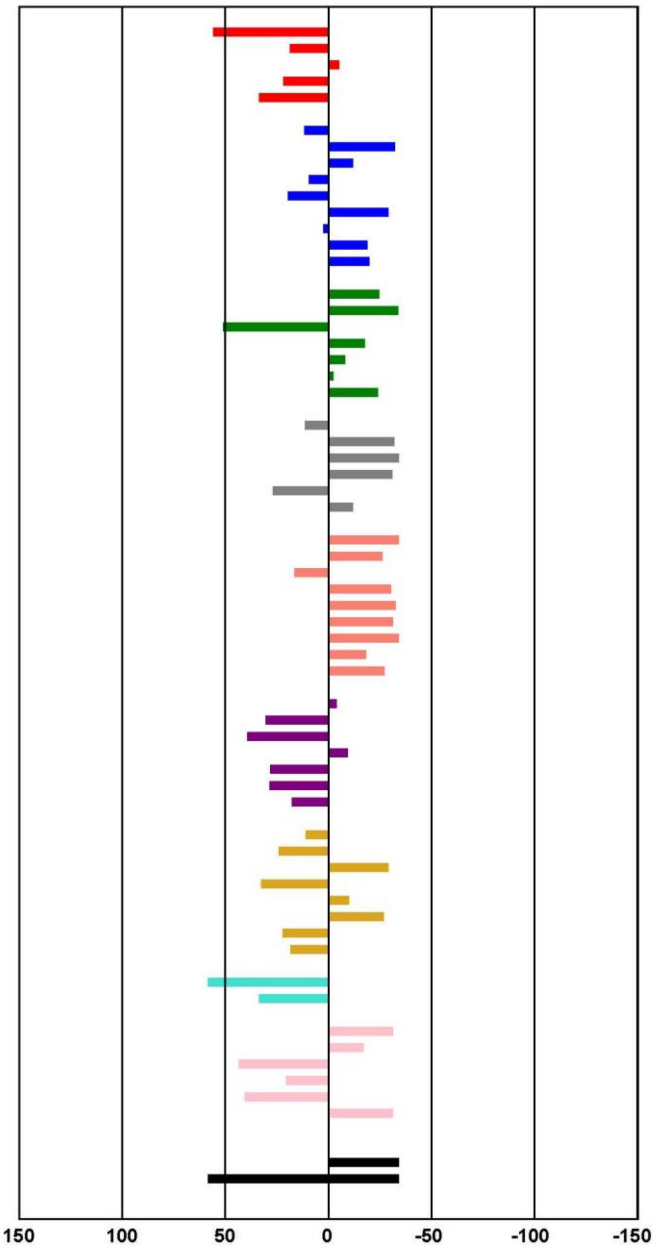

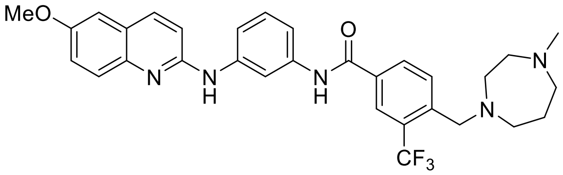

HJ_2-33 = 2-47 (16b)

Developmental Therapeutics Program One Dose Mean Graph	NSC: D-838088 / 1	Conc: 1.00E-5 Molar	Test Date: May 23, 2022
	Experiment ID: 2205OS30		Report Date: Jun 24, 2022

Panel/Cell Line	Growth Percent
Leukemia	
CCRF-CEM	-43.64
HL-60(TB)	-28.00
K-562	-18.57
MOLT-4	-4.91
RPMI-8226	-31.66
SR	4.09
Non-Small Cell Lung Cancer	
A549/ATCC	-24.84
EKVX	-98.52
HOP-62	-47.31
HOP-92	-53.05
NCI-H226	-39.80
NCI-H23	-71.94
NCI-H322M	-86.41
NCI-H460	8.75
NCI-H522	-50.66
Colon Cancer	
COLO 205	-31.70
HCC-2998	-62.55
HCT-116	-47.07
HCT-15	-64.74
HT29	-69.70
KM12	-47.54
CNS Cancer	
SF-268	-9.02
SF-295	-53.92
SF-539	-56.19
SNB-19	-76.44
SNB-75	-35.32
U251	1.47
Melanoma	
MALME-3M	-58.29
M14	-49.39
MDA-MB-435	-42.83
SK-MEL-2	-74.53
SK-MEL-28	-74.92
SK-MEL-5	-69.24
UACC-257	-33.96
UACC-62	-61.10
Ovarian Cancer	
IGROV1	-56.38
OVCAR-3	-48.84
OVCAR-4	-94.39
OVCAR-5	-84.45
OVCAR-8	2.26
NCI/ADR-RES	-43.10
SK-OV-3	-62.44
Renal Cancer	
786-0	-73.39
A498	-53.33
ACHN	-91.53
CAKI-1	-65.17
RXF 393	-82.92
SN12C	-65.88
TK-10	-81.12
UO-31	-66.86
Prostate Cancer	
PC-3	-56.99
DU-145	-54.21
Breast Cancer	
MCF7	-62.82
MDA-MB-231/ATCC	-75.65
BT-549	-61.37
T-47D	-44.62
MDA-MB-468	-71.14
Mean	-52.59
Delta	45.93
Range	107.27

Mean Growth Percent - Growth Percent

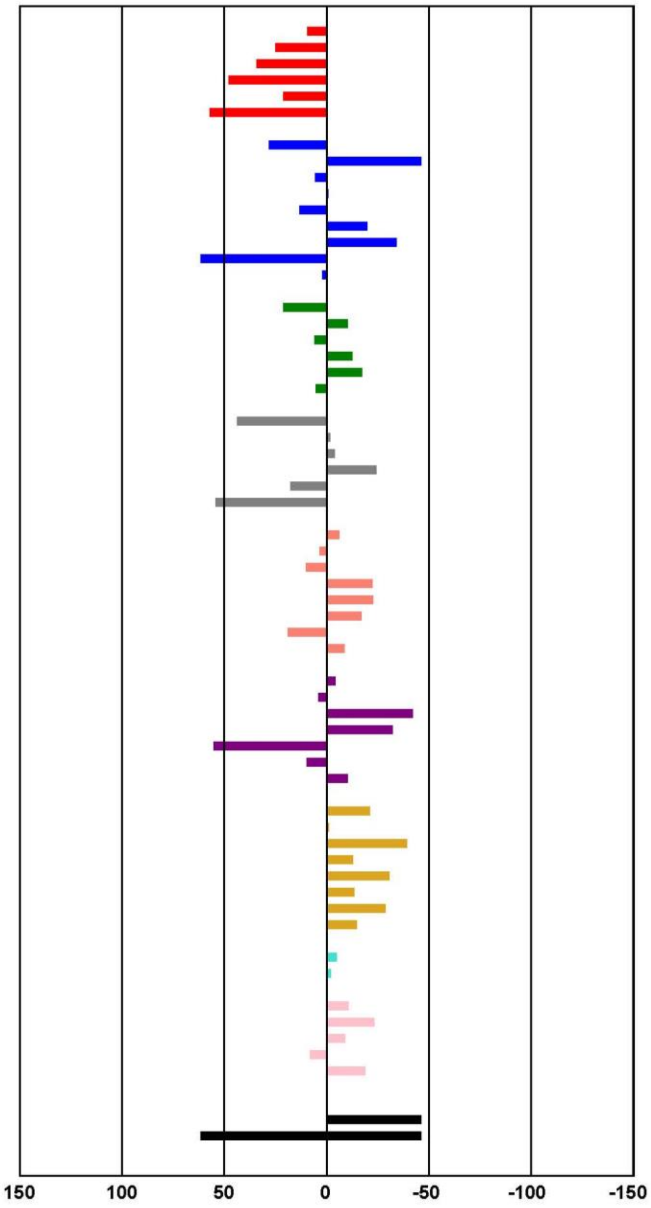

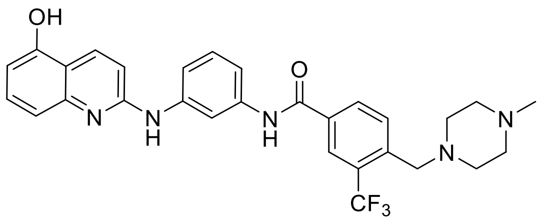

HJ_2-14 (17a)

Developmental Therapeutics Program<br>One Dose Mean Graph	NSC: D-834512 / 1	Conc: 1.00E-5 Molar	Test Date: Feb 28, 2022
	Experiment ID: 2202OS75		Report Date: Mar 17, 2022

Panel/Cell Line	Growth Percent
Leukemia	
CCRF-CEM	5.12
HL-60(TB)	-34.08
K-562	8.33
MOLT-4	-2.84
RPMI-8226	-10.64
SR	4.88
Non-Small Cell Lung Cancer	
A549/ATCC	-14.16
EKVX	-45.64
HOP-62	-47.18
HOP-92	-37.27
NCI-H226	41.26
NCI-H23	-69.79
NCI-H322M	-18.64
NCI-H460	-18.48
NCI-H522	-83.95
Colon Cancer	
COLO 205	-78.63
HCC-2998	-93.17
HCT-116	-43.93
HCT-15	-25.41
HT29	-50.81
KM12	-76.71
SW-620	-84.01
CNS Cancer	
SF-268	2.20
SF-295	-69.25
SF-539	-91.54
SNB-19	-64.31
SNB-75	33.11
U251	-35.26
Melanoma	
LOX IMVI	-100.00
MALME-3M	-95.91
M14	-87.35
MDA-MB-435	-91.56
SK-MEL-2	-75.85
SK-MEL-28	-94.99
SK-MEL-5	-100.00
UACC-257	-78.74
UACC-62	-79.87
Ovarian Cancer	
IGROV1	-54.62
OVCAR-3	-22.96
OVCAR-4	-13.10
OVCAR-5	-48.74
OVCAR-8	-19.53
NCI/ADR-RES	-0.43
SK-OV-3	-46.56
Renal Cancer	
786-0	-55.94
A498	-33.24
ACHN	-23.40
CAKI-1	-38.28
SN12C	1.56
TK-10	-21.03
UO-31	-20.21
Prostate Cancer	
PC-3	10.84
DU-145	-1.97
Breast Cancer	
MCF7	-67.08
MDA-MB-231/ATCC	-59.52
HS 578T	-37.51
BT-549	-58.41
T-47D	-36.84
MDA-MB-468	-92.68
Mean	-43.13
Delta	56.87
Range	141.26

Mean Growth Percent - Growth Percent

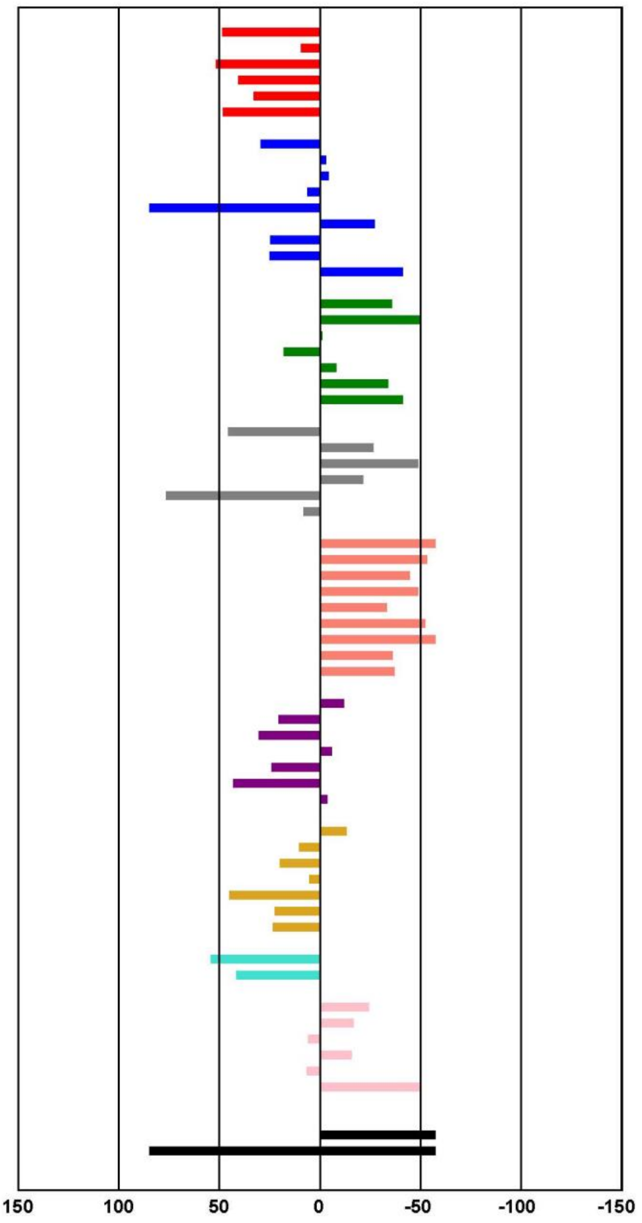

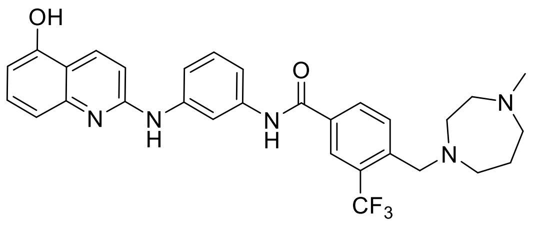

HJ_2-12 (17b)

Developmental Therapeutics Program

One Dose Mean Graph

**NSC:** D-834507 / 1	**Conc:** 1.00E-5 Molar	**Test Date:** Feb 28, 2022
**Experiment ID:** 2202OS75		**Report Date:** May 01, 2022

Panel/Cell Line	Growth Percent
Leukemia	
CCRF-CEM	8.39
HL-60(TB)	-7.86
K-562	11.45
MOLT-4	9.64
RPMI-8226	2.61
SR	11.45
Non-Small Cell Lung Cancer	
A549/ATCC	-61.38
EKVX	-87.02
HOP-62	-69.80
HOP-92	-51.50
NCI-H226	57.38
NCI-H23	-82.13
NCI-H322M	-72.12
NCI-H460	-85.28
NCI-H522	-83.36
Colon Cancer	
COLO 205	-64.41
HCC-2998	-85.28
HCT-116	-67.42
HCT-15	-55.58
HT29	-55.21
KM12	-10.98
SW-620	-92.71
CNS Cancer	
SF-268	-55.62
SF-295	-90.98
SF-539	-98.31
SNB-19	-81.25
SNB-75	13.88
U251	-65.67
Melanoma	
LOX IMVI	-95.50
MALME-3M	-95.91
M14	-84.70
MDA-MB-435	-96.51
SK-MEL-2	-76.45
SK-MEL-28	-100.00
SK-MEL-5	-100.00
UACC-257	-81.34
UACC-62	-94.82
Ovarian Cancer	
IGROV1	-78.92
OVCAR-3	-35.81
OVCAR-4	-43.53
OVCAR-5	-82.10
OVCAR-8	-21.98
NCI/ADR-RES	37.65
SK-OV-3	-58.95
Renal Cancer	
786-0	-74.83
A498	61.78
ACHN	-74.73
CAKI-1	-85.26
SN12C	-86.14
TK-10	-65.27
UO-31	-56.07
Prostate Cancer	
PC-3	2.18
DU-145	-44.46
Breast Cancer	
MCF7	-96.48
MDA-MB-231/ATCC	-75.62
HS 578T	-41.18
BT-549	-75.22
T-47D	-27.72
MDA-MB-468	-94.52
Mean	-55.11
Delta	44.89
Range	161.78

Mean Growth Percent - Growth Percent

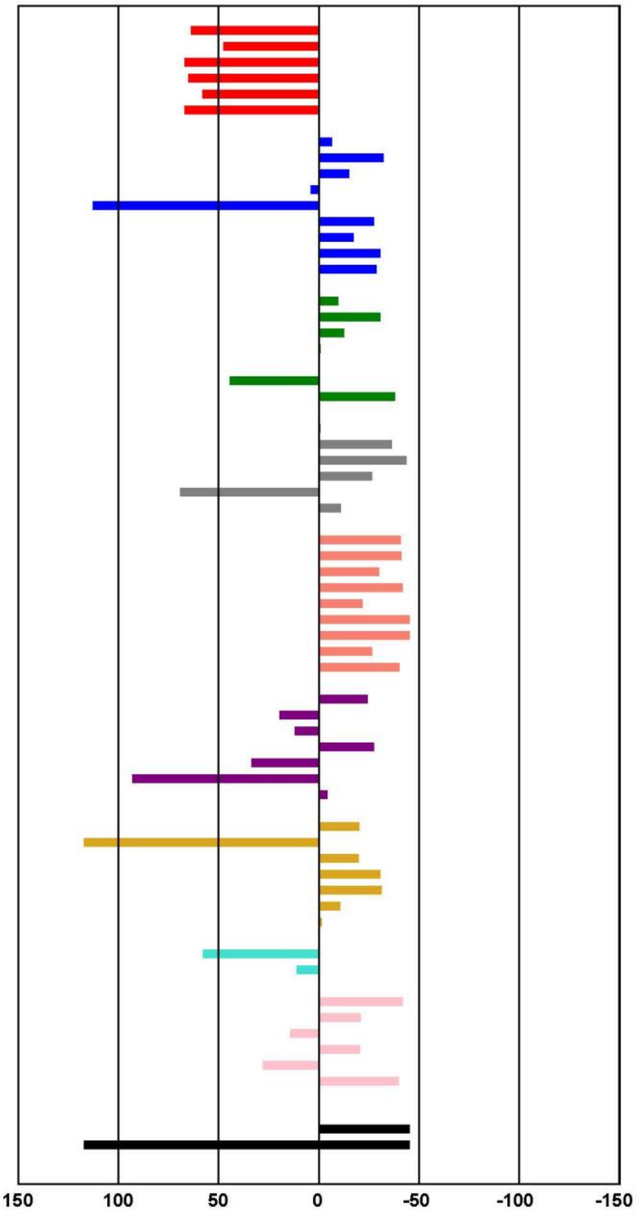

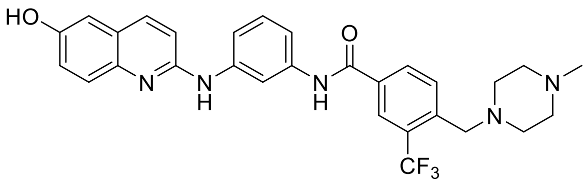
HO
N
N
H
N
H
O
N
N
CF3

HJ_2-56 (18a)

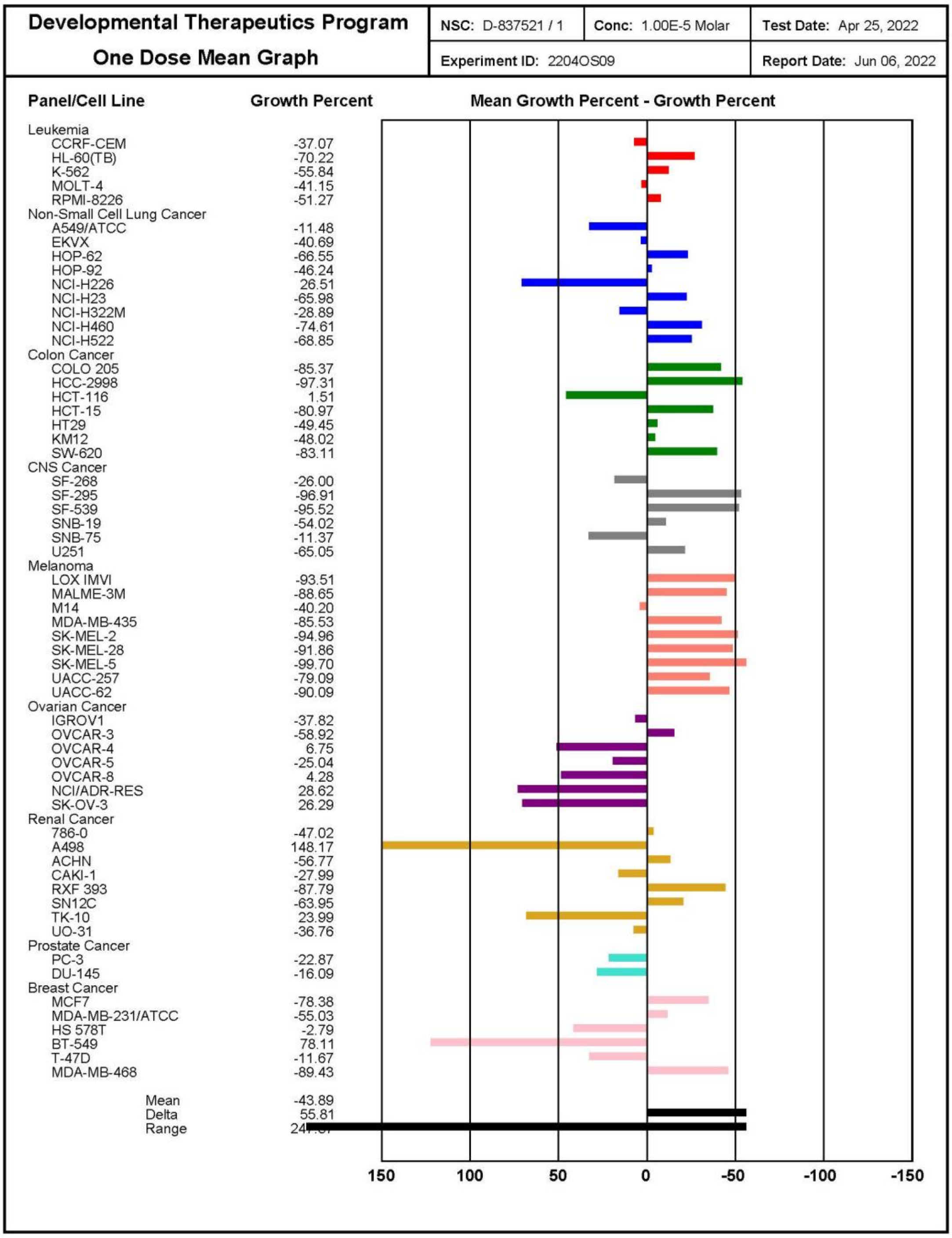
Developmental Therapeutics Program
One Dose Mean Graph
NSC: D-837521 / 1
Conc: 1.00E-5 Molar
Test Date: Apr 25, 2022
Experiment ID: 2204OS09
Report Date: Jun 06, 2022
Panel/Cell Line
Growth Percent
Mean Growth Percent - Growth Percent
Leukemia
CCRF-CEM -37.07
HL-60(TB) -70.22
K-562 -55.84
MOLT-4 -41.15
RPMI-8226 -51.27
Non-Small Cell Lung Cancer
A549/ATCC -11.48
EKVX -40.69
HOP-62 -66.55
HOP-92 -46.24
NCI-H226 26.51
NCI-H23 -65.98
NCI-H322M -28.89
NCI-H460 -74.61
NCI-H522 -68.85
Colon Cancer
COLO 205 -85.37
HCC-2998 -97.31
HCT-116 1.51
HCT-15 -80.97
HT29 -49.45
KM12 -48.02
SW-620 -83.11
CNS Cancer
SF-268 -26.00
SF-295 -96.91
SF-539 -95.52
SNB-19 -54.02
SNB-75 -11.37
U251 -65.05
Melanoma
LOX IMVI -93.51
MALME-3M -88.65
M14 -40.20
MDA-MB-435 -85.53
SK-MEL-2 -94.96
SK-MEL-28 -91.86
SK-MEL-5 -99.70
UACC-257 -79.09
UACC-62 -90.09
Ovarian Cancer
IGROV1 -37.82
OVCAR-3 -58.92
OVCAR-4 6.75
OVCAR-5 -25.04
OVCAR-8 4.28
NCI/ADR-RES 28.62
SK-OV-3 26.29
Renal Cancer
786-0 -47.02
A498 148.17
ACHN -56.77
CAKI-1 -27.99
RXF 393 -87.79
SN12C -63.95
TK-10 23.99
UO-31 -36.76
Prostate Cancer
PC-3 -22.87
DU-145 -16.09
Breast Cancer
MCF7 -78.38
MDA-MB-231/ATCC -55.03
HS 578T -2.79
BT-549 78.11
T-47D -11.67
MDA-MB-468 -89.43
Mean -43.89
Delta 55.81
Range
150
100
50
0
-50
-100
-150

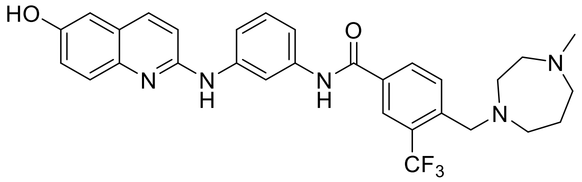

HJ_2-57 (18b)

Developmental Therapeutics Program	NSC: D-837522 / 1	Conc: 1.00E-5 Molar	Test Date: Apr 25, 2022
One Dose Mean Graph	Experiment ID: 2204OS09		Report Date: Jun 06, 2022

Panel/Cell Line	Growth Percent
Leukemia	
CCRF-CEM	-9.62
HL-60(TB)	73.73
K-562	14.82
MOLT-4	-6.61
RPMI-8226	-20.54
Non-Small Cell Lung Cancer	
A549/ATCC	64.72
EKVX	71.13
HOP-62	95.93
HOP-92	53.32
NCI-H226	102.66
NCI-H23	68.80
NCI-H322M	89.07
NCI-H460	27.68
NCI-H522	80.32
Colon Cancer	
COLO 205	63.17
HCC-2998	-15.09
HCT-116	3.55
HCT-15	14.56
HT29	27.78
KM12	25.84
SW-620	-25.83
CNS Cancer	
SF-268	62.47
SF-295	-68.50
SF-539	41.82
SNB-19	81.15
SNB-75	32.20
U251	33.74
Melanoma	
LOX IMVI	-100.00
MALME-3M	51.03
M14	47.24
MDA-MB-435	37.93
SK-MEL-2	111.43
SK-MEL-28	-67.92
SK-MEL-5	32.37
UACC-257	59.76
UACC-62	88.12
Ovarian Cancer	
IGROV1	88.62
OVCAR-3	76.87
OVCAR-4	77.21
OVCAR-5	130.93
OVCAR-8	101.45
NCI/ADR-RES	90.70
SK-OV-3	115.75
Renal Cancer	
786-0	8.41
A498	139.08
ACHN	86.36
CAKI-1	74.70
RXF 393	60.80
SN12C	86.75
TK-10	133.55
UO-31	91.68
Prostate Cancer	
PC-3	51.30
DU-145	73.71
Breast Cancer	
MCF7	-22.78
MDA-MB-231/ATCC	34.91
HS 578T	61.90
BT-549	97.98
T-47D	88.75
MDA-MB-468	5.76
Mean	50.79
Delta	150.79
Range	239.08

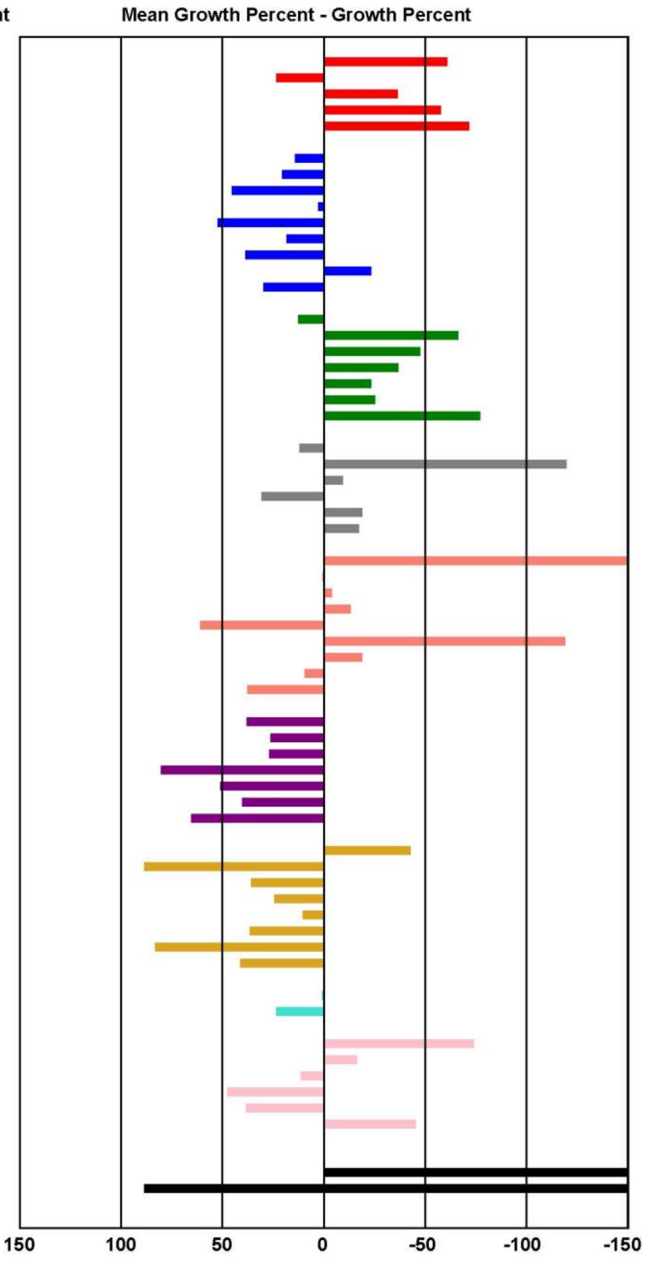

5) Molecular docking

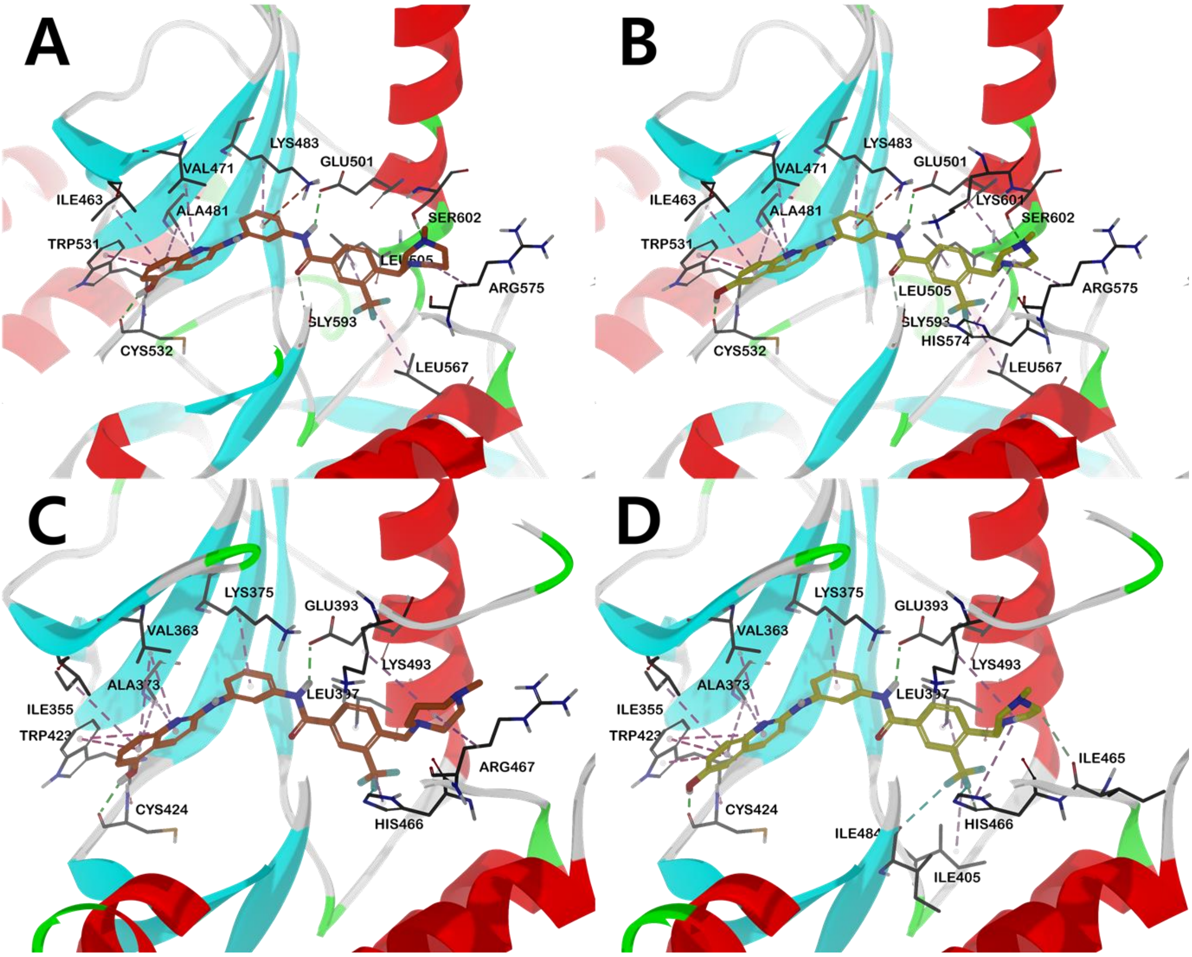

Figure S1. The binding mode of compounds (A) **17b** and (B) **18a** in the catalytic kinase domain of B-RAF^{V600E} model. The binding mode of compound (C) **17b** and (D) **18a** in the C-RAF homology model. For clarity purpose, the residues showing interactions were shown. Compounds **17b** and **18a** are shown in orange and yellow colored stick model. Binding interactions are shown in various colored dashes lines, crucial hydrogen bonding shown in green dashes lines.

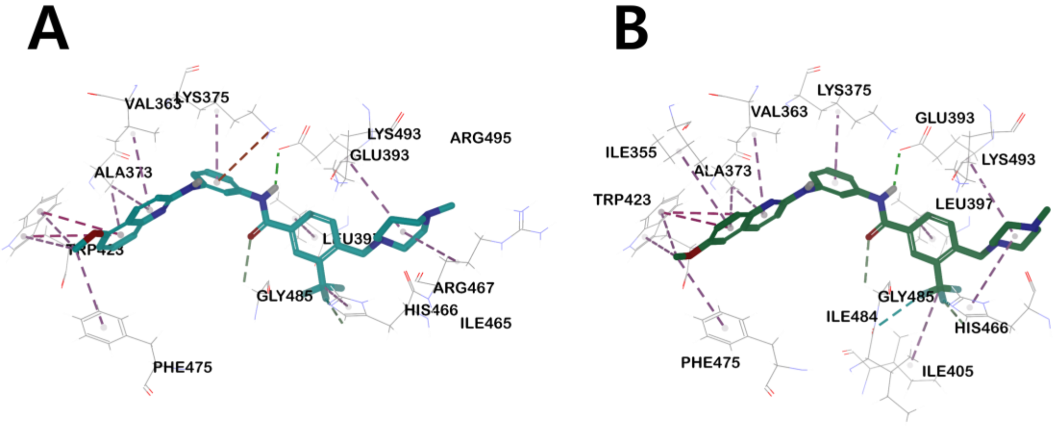

Figure S2. Binding model of compounds (A) **14b** and (B) **16a** in the C-RAF homology model. Compounds **14b** and **16a** shown by sky-blue and green colored stick model, respectively. The surrounding residues were shown as line model and various interactions shown as dashes line.

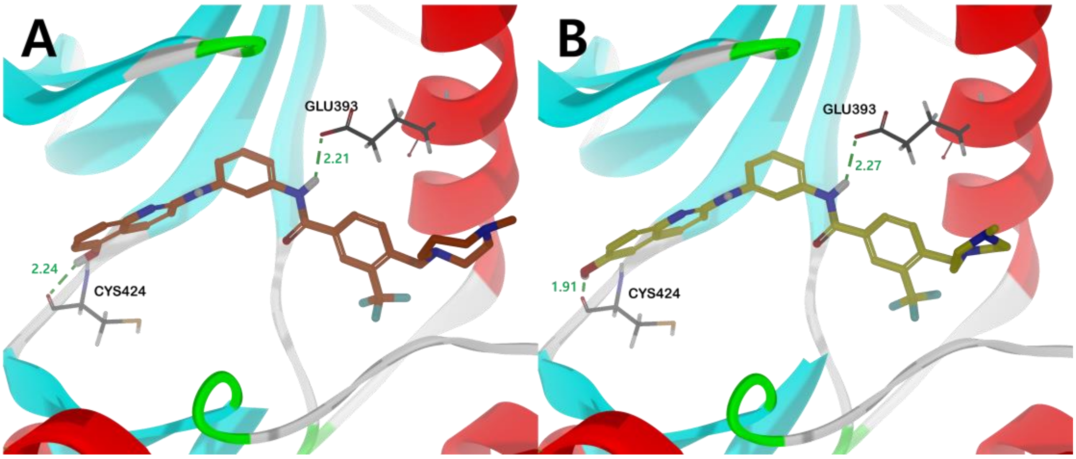

Figure S3. Binding model of compounds (A) **17b** and (B) **18a** in the C-RAF homology model. For clarity purpose, only key residues showing interactions were shown. Compounds **17b** and **18a** are shown in orange and yellow colored stick model. Key hydrogen bonding interactions are shown in green dashes. The number near the dashes indicates the bonding distance, in units of Å.

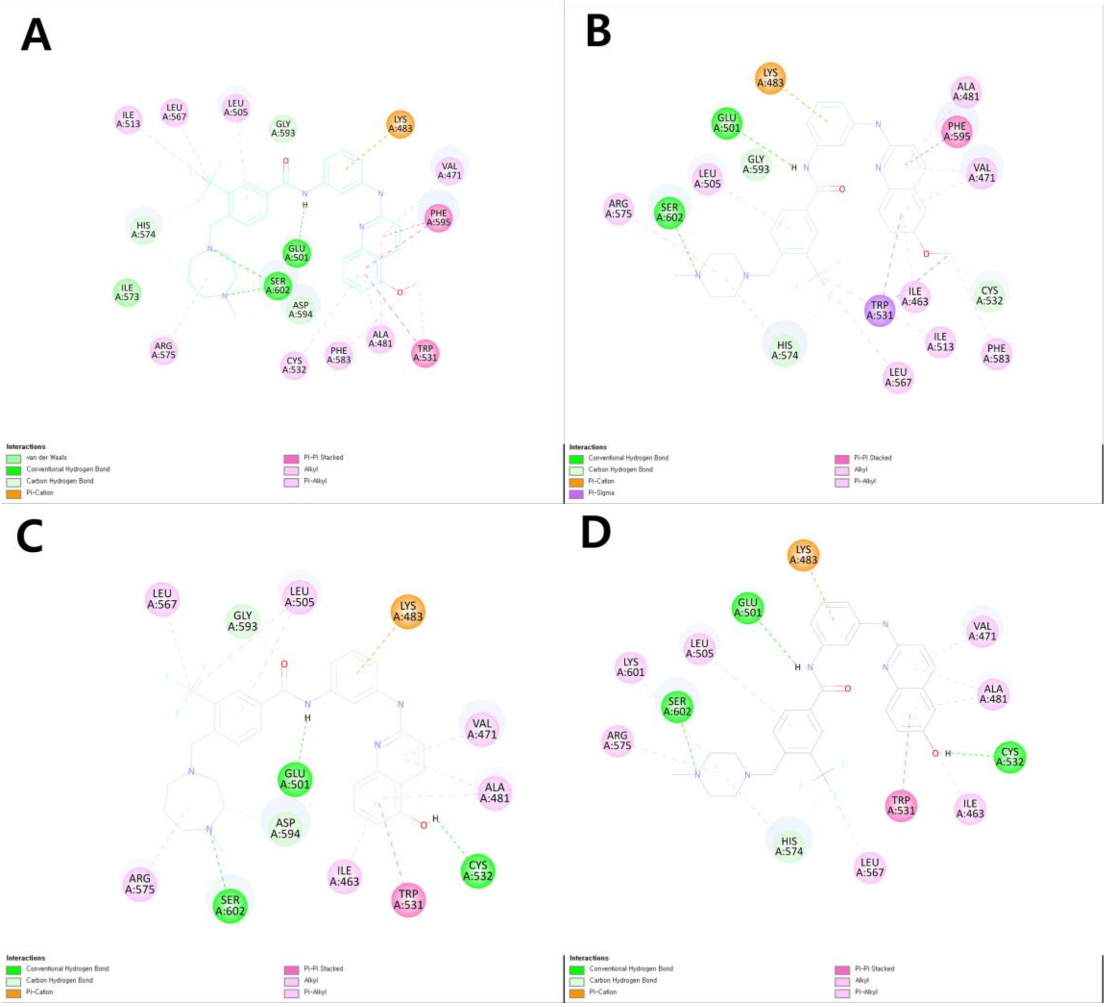

Figure S4. 2D binding mode of compound (A) **14b**, (B) **16a**, (C) **17b** and (D) **18a** with B-RAF^{V600E}. Various interactions are depicted by different color legends. The inhibitors are shown by line, interacting residues by colored sphere, and interactions by dash lines.

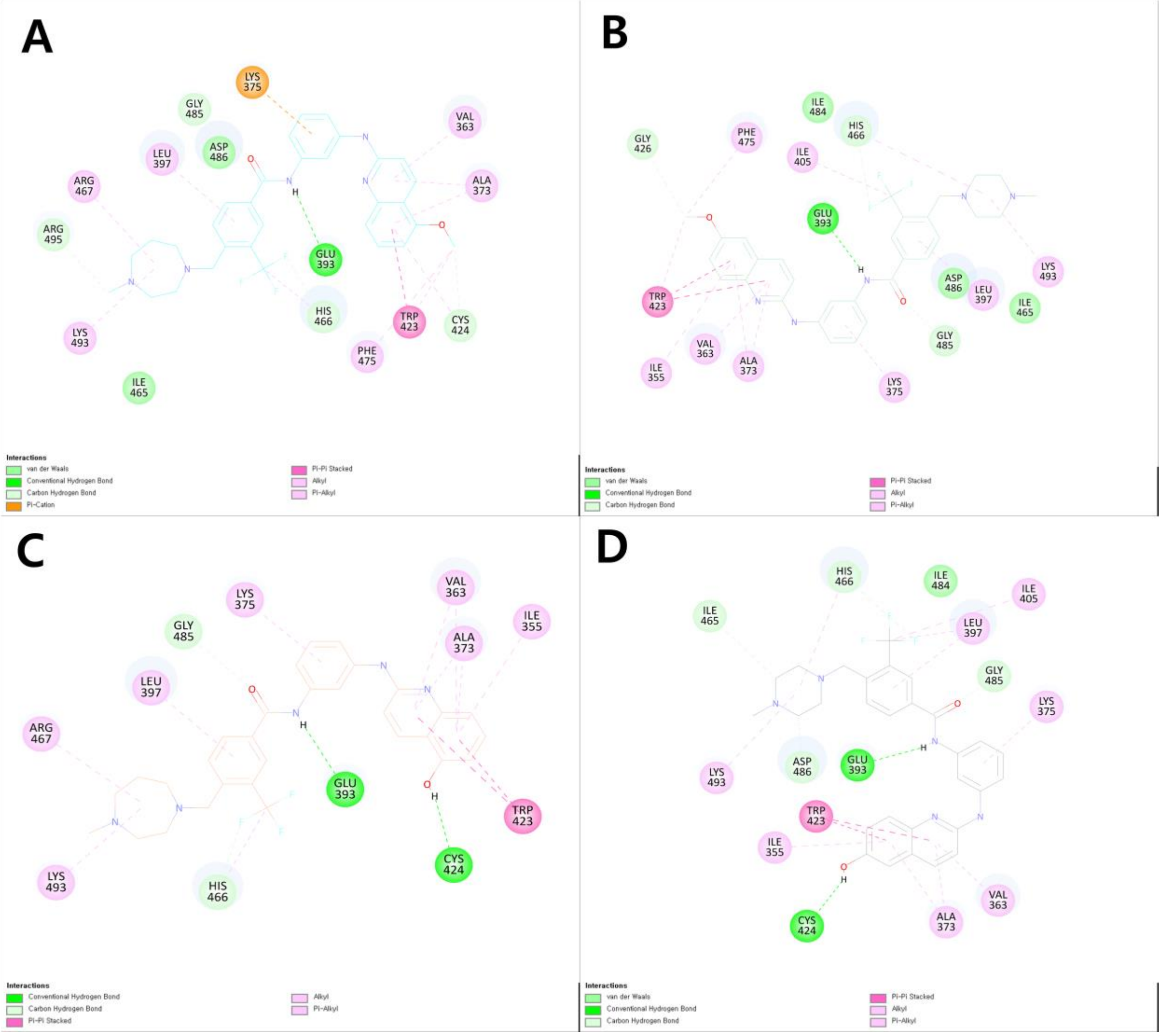

Figure S5. 2D binding mode of compounds (A) **14b**, (B) **16a**, (C) **17b** and (D) **18a** with C-RAF homology model. Various interactions are depicted by different color legends. Inhibitors are shown by line, interacting residues by colored sphere, and interactions by dash lines.

6) Molecular dynamics

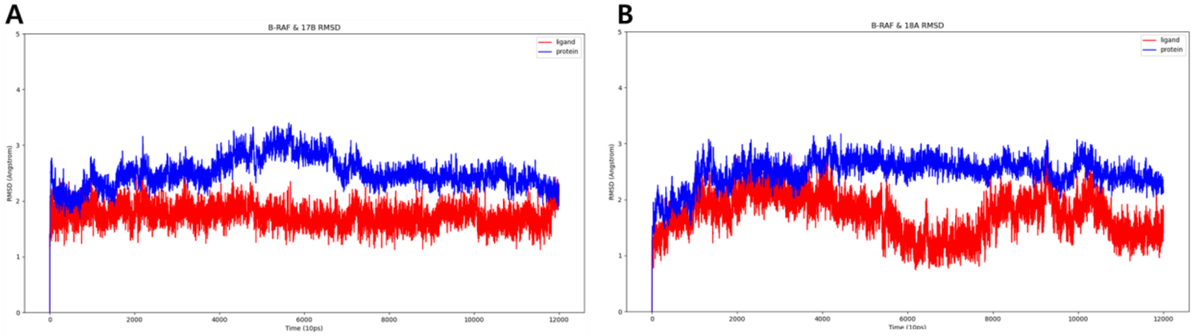

Figure S6. RMSD values of (A) **17b** and (B) **18a** compounds bounded B-RAF^{V600E} protein during 120 ns of molecular dynamics simulation.

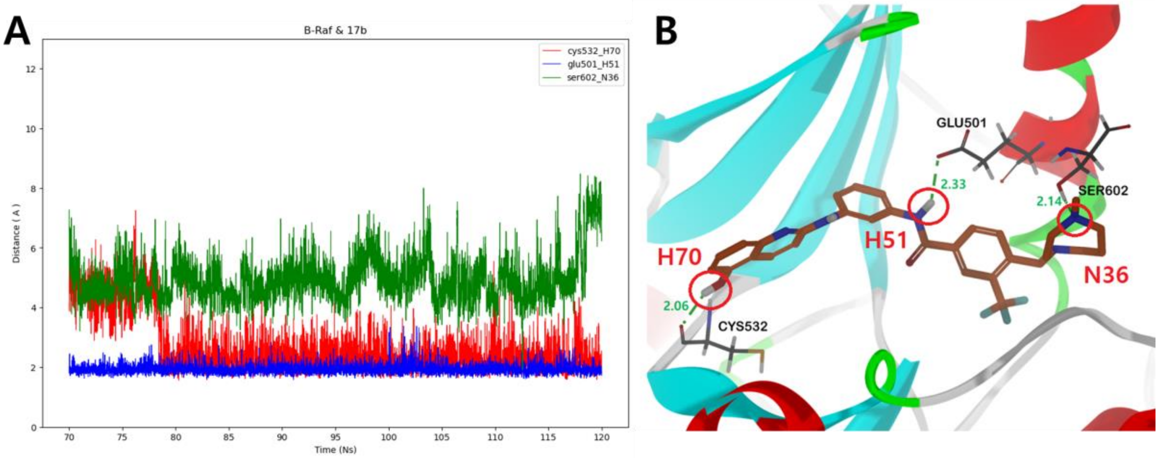

Figure S7. (A) Distance of three key hydrogen bonding interaction between B-RAF^{V600E} protein and **17b** during 70-120 ns dynamics simulation. The distances between Cys532 and H70, Glu501 and H51, and Ser602 and N36 are marked in red, blue, and green, respectively. (B) Initial binding structure of **17b** and B-RAF ^{V600E} used in dynamics simulation. Atoms participating in three hydrogen bonding bonds whose distances are monitored are marked with red circles.

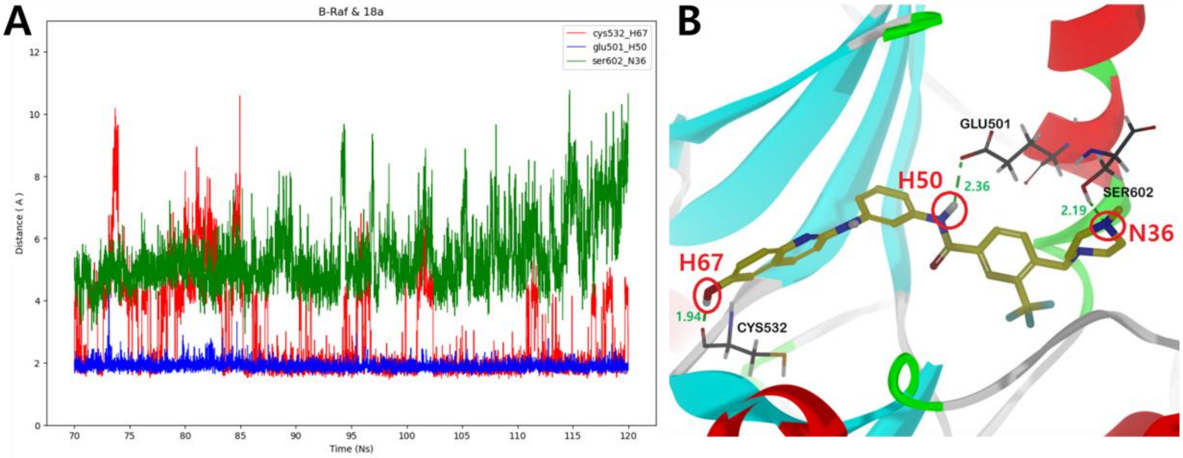

Figure S8. (A) Distance of three key hydrogen bonding interaction between B-RAF^{V600E} protein and **18a** during 70-120 ns dynamics simulation. The distances between Cys532 and H67, Glu501 and H50, and Ser602 and N36 are marked in red, blue, and green, respectively. (B) Initial binding structure of **18a** and B-RAF ^{V600E} used in dynamics simulation. Atoms participating in three hydrogen bonding bonds whose distances are monitored are marked with red circle.
